# Knowledge attributes of public health management information systems used in health emergencies: a scoping review

**DOI:** 10.3389/fpubh.2024.1458867

**Published:** 2025-03-20

**Authors:** Barbara Burmen, Elliot Brennan, Maryam Mohammed Samaila, Allan Bell, Candice Vente, Landry Ndriko Mayigane

**Affiliations:** World Health Organization, Geneva, Switzerland

**Keywords:** knowledge dimensions, knowledge management systems, health emergencies, experiential knowledge, tacit knowledge

## Abstract

**Introduction:**

Learning from public health emergencies has not always been possible due to suboptimal knowledge accrual from previous outbreaks. This study described the knowledge attributes of Health Management Information Systems (HMIS) that are currently used during health emergencies. It aims to inform the development of a “nuggets of knowledge” (NoK) platform to support agile decision-making and knowledge continuity following health emergencies.

**Methods:**

A search was conducted on the Web of Science and Google Scholar, with no date restriction for articles that conveniently selected 13 HMIS and their knowledge attributes. Proportions were used to summarize HMIS distribution by countries’ World Bank income status. Thematic content analysis was used to describe knowledge attributes of HMIS based on the knowledge attributes of Holsapple et al.

**Results:**

Seven of the 13 HMIS contained tacit knowledge; the 7 HMIS were predominantly used in higher-income settings and developed after explicit knowledge containing HMIS. More HMISs that contained tacit knowledge were currently usable, universal, programmable, user-friendly, and relied on informal information sources than HMIS that contained explicit knowledge HMIS. Tacit and explicit knowledge containing HMIS were equally practical, accessible, and domain-oriented.

**Conclusion:**

HMIS should continuously capture both tacit and explicit knowledge that is actionable and practical in HMIS, user-friendly, programmable, and accessible to persons in all geographical settings. HMIS that contain tacit knowledge have more favorable attributes than those that contain explicit knowledge, but they may not be available to all emergency responders globally, a distribution that may change as newer low-cost technologies become available. Future research should investigate the impact of the NoK platform on public health emergency management.

## 1 Introduction

Knowledge failures have been evident during past health emergencies. Since Ebola viruses were first described in 1976, more than 20 Ebola Virus Disease (EVD) outbreaks have occurred in 19 countries globally, with some countries experiencing multiple outbreaks (1). The 2014–2016 West African Ebola outbreak recorded very high morbidity and mortality rates (2). However, the mortality rates of the 2018 Ebola outbreak in DRC, which lasted more than 18 months (up to 70%), exceeded that of the 2014–2016 West Africa outbreak despite the availability of vaccines and therapeutics that were not present in the previous outbreak (3). In 2003, a coronavirus, severe acute respiratory syndrome coronavirus disease (SARS-CoV), had more than 8,000 cases and more than 700 deaths, with a case fatality rate of 9.7% in 6 countries and a few cases identified on flights to the WHO European region (4). In 2012, another coronavirus, Middle East respiratory syndrome coronavirus (MERS-CoV), resulted in cases and deaths surpassing 2000 and 800, respectively, and case fatality rates of 34% in 27 countries (5). In 2020, the coronavirus disease 2019 (COVID-19) pandemic resulted in 774 million cases and 7 million deaths globally by 14th January 2024 (6). The pandemic was a crisis of knowledge in all its elements (people, processes, and technology), as evidenced by the lack of basic and scientific knowledge about a vaccine or cure. Moreover, in the absence of a vaccine, social science-based knowledge became crucial. Nevertheless, there was limited social knowledge of the processes required to implement public health and social measures. Furthermore, there was limited information that could inform public health policy measures (7).

However, there have been instances of effective knowledge management during past emergencies. Countries have used systems that were developed in response to past emergencies to address new health threats. After the 2014–2016 West Africa EVD epidemic devastated Guinea’s health system, Guinea invested in strengthening its national health security. Thus, Guinea’s 2021 EVD outbreak had a lower case burden and mortality rate (8). During the COVID-19 pandemic, governments worldwide not only relied on existing systems and resources but also developed innovative new solutions and strategies to address the pandemic (9). During the first wave of COVID-19, South Korea, Taiwan, Singapore, and Hong Kong implemented lessons learned from SARS/MERS that had an impact on the number of infected cases and deaths reported at the end of the first 6 months of COVID-19 (10). Similarly, lessons learned from the disastrous MERS outbreak were used to develop International Health Regulations (2005) [IHR (2005)] capacities in the Republic of Korea’s preparedness system that enabled the country to successfully flatten the epidemic curve of COVID-19 (11).

Learning from public health emergencies has not always been possible due to limited access to knowledge accrued from previous outbreaks. Hence, emergency response personnel have always been forced to reinvent the wheel in subsequent emergencies (12, 13). Limited knowledge accrual could be attributed to the absence of systematic documentation of critical knowledge learned during health emergencies. During emergencies, decisions are made by emergency response personnel from different sectors, organizations, and sometimes countries who may be interacting for the first time. The majority of reports made after emergencies do not document the ‘informal’ rules (and strategies) that govern the ability to function in such emergencies. After the emergency has waned, emergency response personnel may either revert to their previous roles and routines, leave the organizations to pursue new roles, or retire. Subsequently, such organizational memory is lost forever (14). Such knowledge that includes mental models, perspectives, intuitions, experience, and know-how that has not yet been articulated is known as tacit knowledge. The knowledge that has been articulated and documented is known as explicit knowledge (15).

The World Health Organization’s knowledge management strategy recommends improving global access to health information and sharing experiential and applied knowledge by leveraging e-health (16). The World Health Organization is developing a ‘Nuggets’ of Knowledge (NoK) platform to harness the contextual knowledge of emergency response personnel gained from health emergencies to facilitate continuity and preservation of tacit knowledge. Health management information systems (HMIS) are systems that support the recording, storage, retrieval, and processing of health information to support decision-making, which is also one of the six building blocks of the health system that provide data needed for the other five components (service delivery, health workforce, finance, leadership, and access to essential medicines) (17). Knowledge attributes (18) influence the ease with which knowledge moves within and across firm boundaries, how much of it is retained, and the rate at which knowledge is reactivated and transformed and thus could guide the configuration of technological learning routines for innovation and optimal organizational performance (19). This scoping review evaluated the knowledge attributes of select public health management information systems (HMIS) used in health emergencies that contain tacit and explicit knowledge to inform the development of the NoK platform to support agile decision-making and knowledge continuity following health emergencies. We limited our review to 13 purposively selected HMIS among numerous existing HMIS for feasibility purposes.

## 2 Methods

### 2.1 Study design

**Figure 1:**
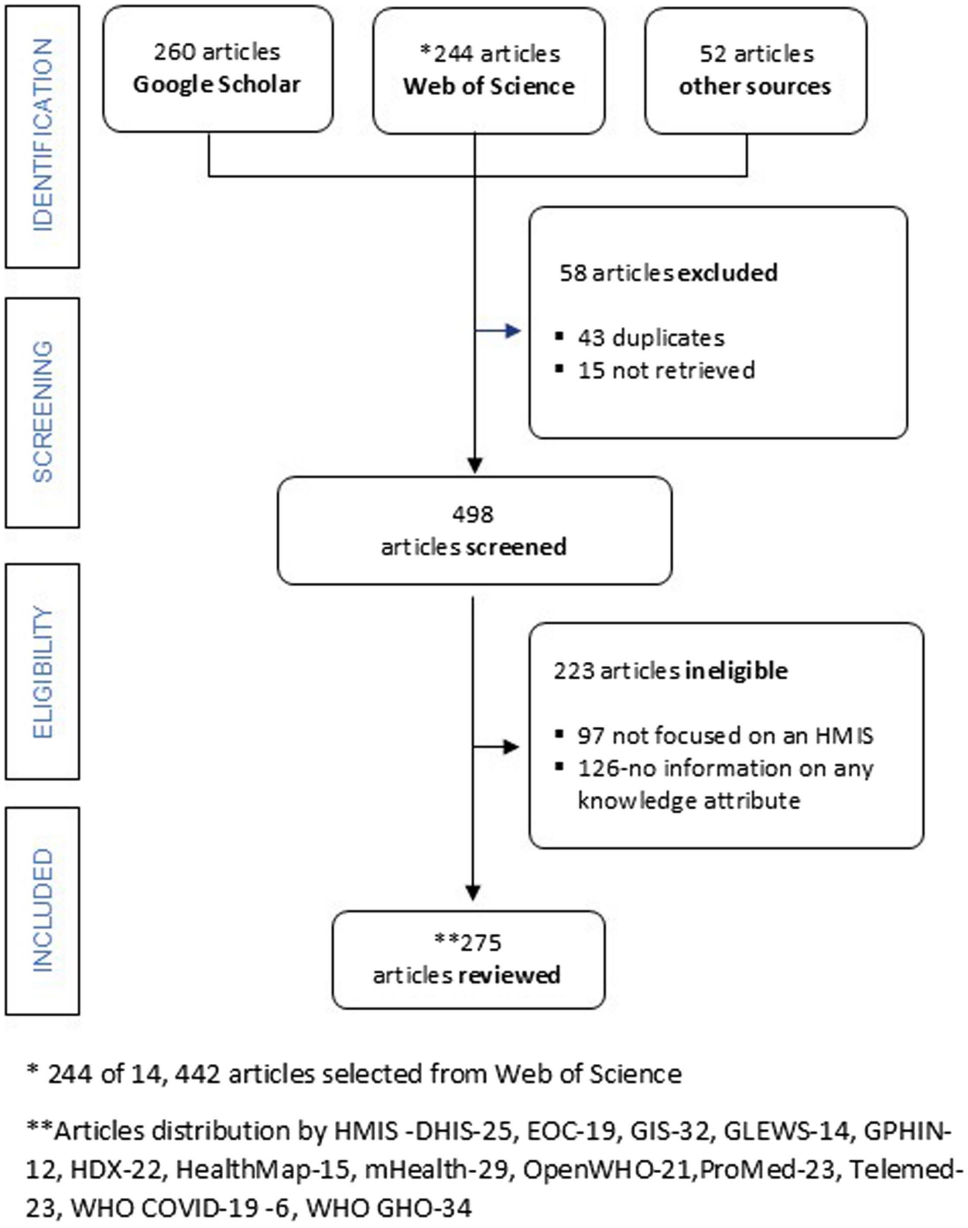
A PRISMA-ScR flow chart. DHIS, District Health Information System; EOC, Emergency Operations Center; GIS, Geographical Information System; GLEWS, Global Early Warning System; GPHIN, Global Public Health Intelligence Network; HDX, Humanitarian Data Exchange; HealthMap, an automated electronic information system for monitoring, organizing, and visualizing reports of global disease outbreaks; mHealth-Mobile health applications; OpenWHO, World Health Organization’s online learning platform; ProMED, ProMED mail: Program for Monitoring Emerging Diseases; Telemed, telemedicine platforms; WHO COVID-19, World Health Organization COVID-19 dashboard; WHO GHO, World Health Organization Global Health Observatory.

A scoping review (20) was conducted to characterize 13 conveniently selected HMIS used in health emergencies (Figure 1 and Supplementary Tables A1, A2). Arksey and O’Malley’s (21) six-stage framework, the World Health Organization’s guidance for rapid reviews (20), and a Preferred Reporting Items for Systematic Reviews and Meta-Analyses Extension for scoping reviews (PRISMA-ScR) approach (22) guided our methodology. The study protocol was not registered.

### 2.2 Research question

The study’s research question was, ‘*What are the knowledge attributes of HMIS used in health emergencies that contain tacit knowledge and explicit knowledge?’* Health management information systems (HMIS) support the recording, storage, retrieval, and processing of health information to support decision-making (17). Knowledge attributes are dimensions across which knowledge can vary. An attribute dimension can either be on a range, like knowledge age, or be categorical (Table 1) (18). Health emergencies are situations that have an immense impact on the health and lives of many people; addressing such situations requires extensive intervention by multiple sectors (23).

**Table 1:** Knowledge attributes of HMIS evaluated in this scoping review

**Knowledge attribute**	**Nature of dimension**
Mode	Tacit knowledge is knowledge that is inconvenient or difficult to formalize or communicate that has not been articulated. Explicit knowledge is conveyed in formalized systematic representations have been communicated and articulated in knowledge artifacts such as books, computers etc. (15) HMIS knowledge mode was described as either tacit, explicit or a combination of tacit and explicit knowledge.
Type	Descriptive (includes data and information) (15) vs procedural vs reasoning knowledge (15) (i.e., information upon which an emergency responder can act (71)).
Domain	Subject areas where knowledge is used (15) either animal or human health or both (157).
Applicability	Range from local (very localized in its applicability e.g., only used within a country for a specific purpose) to global (universally usable in routine and frequent circumstances e.g., can be used beyond the confines of national borders; international) (15).
Practicality	High practicality- of utility or benefit (contains relevant information that is pertinent to the problem at hand) Moderate practicality -partially contains relevant information that is pertinent to the problem at hand Low practicality - contains very little information that is pertinent to the problem at hand (193)
User-friendliness	High user-friendliness (easy to navigate). Moderate user-friendliness -slightly challenging to navigate. Low user-friendliness challenging to navigate (193)
Accessibility	Range from private (closed source-accessible to a single processer) to public (open source-accessible to any processor) (15).
Source	Knowledge originates either from informal or formal sources (150)
Immediacy	Knowledge is either potentially usable (latent) or currently usable (15).
Programmability	Degree to which knowledge is readily transferable and easy to use. Ranges from high (very easy to transfer for use), to moderate (easy to transfer) to low (untransferable) (15).

We used the Population, Exposure, and Outcome (PEO) framework instead of the PICO one suited to clinical research questions. Population referred to what the research question focused on (public health emergencies), the exposure referred to what we were interested in (HMIS), and the outcome referred to what we wanted to examine in relation to the issue they are interested in (knowledge attributes) (24).

### 2.3 Search strategy

Published articles, abstracts, technical reports, newsletters, and other literature sources were identified from two databases, Web of Science and Google Scholar, due to their coverage (25), and the complementary nature of their coverage characteristics (26). One of the earliest pieces of evidence of health information management dates back over 150 years when John Snow first described disease patterns in different geographic locations during a cholera outbreak (27). Therefore, the search was done with no date restriction. The search terms used to retrieve articles included a combination of terms used to describe an HMIS and/or health emergencies, as shown in Supplementary Tables A1, A2. We initially attempted to run one search for all HMIS. However, this did not return relevant articles to our research question. Therefore, we focused our search strategy on each HMIS. The Web of Science search results were exported into Endnote reference management software. Search results from Google Scholar were saved in the reviewer’s library in HMIS-labeled folders and then downloaded into Endnote reference management software. The references’ lists of included studies and reviews identified through the search were also hand-searched to ensure literature saturation. The search was conducted only once, and no subject matter experts were consulted.

### 2.4 Article selection

All types of articles and study designs that reported one or more knowledge attributes of an HMIS in health emergencies were eligible for inclusion. The HMIS reviews were all public and currently in use. Private HMIS or HMIS set up for one-time use were ineligible for inclusion.

An article had to include information against which we could assess one or more knowledge attributes listed in Table 1. An article that contained information about an HMIS but did not describe information that related to at least one knowledge attribute of that HMIS was ineligible for selection.

### 2.5 Data extraction

A single reviewer screened all articles, identified relevant articles (Supplementary Tables B1–B13), and extracted data from each article based on select fields recommended by Peters et al. (28) and information that could be used to characterize the knowledge attributes of each HMIS. Details regarding data extracted are found in Supplementary Tables C1–C9.

### 2.6 Data collation

First, HMIS year of inception and settings of use were summarized (Supplementary Tables D1–D13).

Second, the extracted data in section 2.5 was thematically organized to correspond to the knowledge attributes shown in Table 1 using Thomas and Harden’s (29) methods for the thematic synthesis of qualitative research in systematic reviews (18) (Supplementary Tables C1–C9).

### 2.7 Data synthesis and reporting

A PRISMA-ScR flow chart was used to summarize the article identification, screening, and selection process. A timeline was plotted to illustrate the year of inception of all HMIS. Charts were plotted to illustrate the total number of articles reviewed for all HMIS and for each HMIS by knowledge mode (tacit or explicit knowledge) and the number of articles published per HMIS in the following 5-year intervals: before 2006, 2006–2010, 2011–2015, 2016–2020, and 2021–2014.

Descriptive statistics were used to categorize the distribution of countries where each HMIS was used based on the 2022 World Bank countries’ classification of income status (30). This categorization was done because there is a positive relationship between the adoption of information and communications technology and a country’s economic growth. Therefore, HMIS distribution may differ based on countries’ income status. HMIS may have different knowledge attributes, implying that emergency management personnel have uneven access to knowledge in different parts of the world (31).

Charts were plotted to illustrate knowledge attributes for all HMIS and for HMIS that contained tacit (31) and explicit knowledge (30). The total number of mentions of each specific knowledge attribute (the frequency of citation of each attribute in each article) was computed for all HMIS and for HMIS that contained tacit and explicit knowledge. Descriptive data analysis was done using Microsoft Excel and the Statistical Package for the Social Sciences (SPSS) version 22, whereas the extracted qualitative data was manually analyzed for emerging themes.

## 3 Results

### 3.1 Literature search results

The initial literature search on WoS and Google Scholar identified 556 results; 275 were included in the final analysis after excluding 126 articles that did not contain information on knowledge attributes, 97 articles that were not on any of the HMIS selected, 43 duplicate articles, and 15 articles that could not be retrieved. Article identification, screening and eligibility, and inclusion are shown in Figure 1.

### 3.2 Knowledge modes of HMIS evaluated in this study

The majority of HMIS (7 of 13) incorporated tacit knowledge, including the Global Early Warning System (GLEWS), Global Public Health Intelligence Network (GPHIN), HealthMap (an automated electronic information system for monitoring, organizing, and visualizing reports of global disease outbreaks), mobile health applications (mHealth), OpenWHO (the World Health Organization’s online learning platform), the Program for Monitoring Emerging Diseases (ProMED mail), and telemedicine platforms. The remaining systems—District Health Information System (DHIS), Emergency Operation Center (EOC), Geographical Information System (GIS), Humanitarian Data Exchange (HDX), the World Health Organization Global Health Observatory (WHO GHO), and the World Health Organization COVID-19 dashboard (WHO COVID-19)—were characterized by knowledge (Supplementary Table C1).

### 3.3 HMIS development and information sources

**Figure 2:**
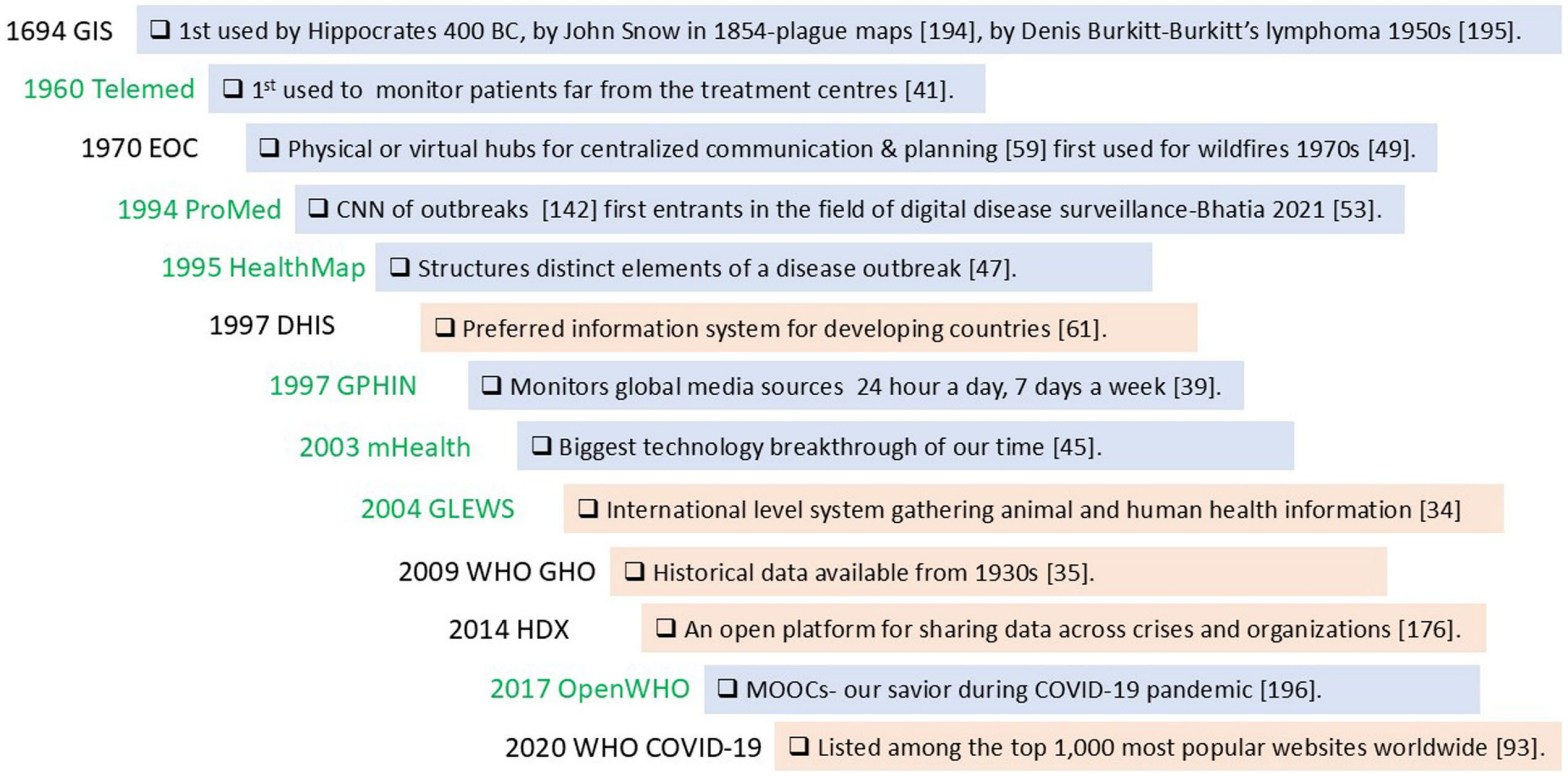
Development timeline, knowledge modes and sources of knowledge for health management information systems (HMIS) used in health emergencies. Tacit knowledge containing HMIS contain knowledge that describes an aspect of reasoning knowledge that has not been articulated (green font) and explicit knowledge containing HMIS contain knowledge that has been articulated and formalized in documents or databases (black font). HMIS that obtain information from formal sources are highlighted in orange while those that obtain information from both formal and informal sources are highlighted in blue. HMIS that contain explicit knowledge (black font) predate HMIS that contain tacit knowledge (green font). More HMIS that contain tacit knowledge obtain knowledge from both formal and informal sources (blue highlights) when compared to HMIS that contain explicit knowledge (orange highlights). DHIS, District Health Information System; EOC, Emergency Operations Center; GIS, Geographical Information System; GLEWS, Global Early Warning System; GPHIN, Global Public Health Intelligence Network; HDX, Humanitarian Data Exchange; HealthMap, an automated electronic information system for monitoring, organizing, and visualizing reports of global disease outbreaks; mHealth-Mobile health applications; OpenWHO, World Health Organization’s online learning platform; ProMED, ProMED mail: Program for Monitoring Emerging Diseases; Telemed, telemedicine platforms; WHO COVID-19, World Health Organization COVID-19 dashboard; WHO GHO, World Health Organization Global Health Observatory (34, 35, 39, 41, 45, 47, 49, 53, 59, 61, 93, 142, 176, 194, 195, 196).

The HMIS that contained explicit knowledge predated HMIS that contained tacit knowledge. More HMIS that contained tacit knowledge obtained knowledge from both formal and informal sources than HMIS that contained explicit knowledge (Figure 2 and Supplementary Table C2).

### 3.4 Number of articles reviewed for all HMIS

**Figure 3:**
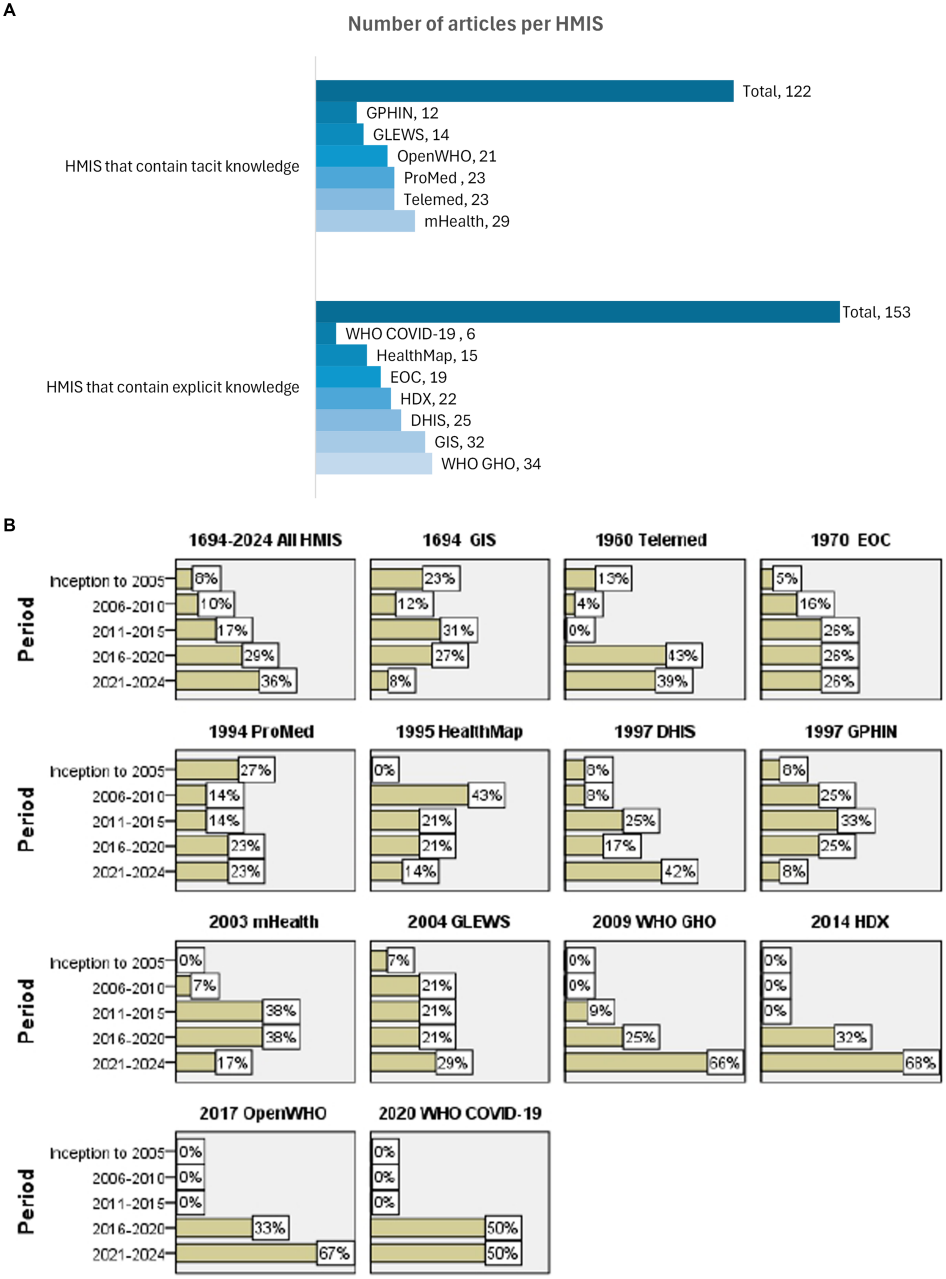
**(A)** Number of articles reviewed in this study for HMIS used in health emergencies containing tacit knowledge (upper panel) and explicit knowledge (lower panel). The figure represents the final number of articles selected for inclusion for each HMIS used in health emergencies. There was a balance between the number of articles reviewed for HMIS that contain tacit and explicit knowledge. A similar number of HMIS from each group had fewer or more articles reviewed. **(B)** HMIS year of development and number of articles published over time. The year of development and the proportion of articles published in different periods as a fraction of the total articles for each HMIS. The figure summarizes the year of inception of each HMIS and number of articles per HMIS reviewed that were published within each 5-year time-period between 2005 to 2024 as follows: before 2006, 2006-2010, 2011-2015, 2016-2020, 2021-2014. An increase in the overall number of publications over the years *possibly signifies* the development of more HMIS. GIS, EOC, ProMed, DHIS, mHealth and GLEWS had a general increase in the number of publications over the five periods. Telemedicine platforms had an initial decrease in the number of articles published in the first two periods followed by a drastic increase in the number of articles in the last two periods. WHO GHO, HDX, OpenWHO, WHO COVID-19 dashboard had articles published in the last two years only. HealthMap had a rapid increase in the number of articles published in the second period with a consistent number of articles in the following years. GPHIN had an initial increase followed by a decrease in the number of articles published over time. DHIS, District Health Information System; EOC, Emergency Operations Center; GIS, Geographical Information System; GLEWS, Global Early Warning System; GPHIN, Global Public Health Intelligence Network; HDX, Humanitarian Data Exchange; HealthMap, an automated electronic information system for monitoring, organizing, and visualizing reports of global disease outbreaks; mHealth-Mobile health applications; OpenWHO, World Health Organization’s online learning platform; ProMED, ProMED mail: Program for Monitoring Emerging Diseases; Telemed, telemedicine platforms; WHO COVID-19, World Health Organization COVID-19 dashboard; WHO GHO, World Health Organization Global Health Observatory.

About half of the articles were on HMIS, whichcontained tacit knowledge (137), while the rest (136) were on HMIS, which contained explicit knowledge (136) (Figure 3A and Supplementary Tables B1–B13).

### 3.5 Number of articles reviewed per HMIS

There was an overall increase in the number of publications for all HMIS over time; 8, 10, 17, 29, and 36% of all articles were published before 2006, during 2006–2010, 2011–2015, 2016–2020, and 2021–2014, respectively. GIS, EOC, ProMED-mail, DHIS, mHealth, and GLEWS showed a general upward trend in publications over time. Articles on WHO GHO, HDX, OpenWHO, and the WHO COVID-19 dashboard were published exclusively from 2016 to 2024. Telemedicine experienced an initial decline in the number of articles published between 2005 and 2015, followed by a drastic increase in the number of articles published between 2016 and 2020 and 2021 and 2024. HealthMap saw a rapid rise in the number of articles published between 2006 and 2010, with consistent publication numbers in subsequent years. GPHIN showed growth in publications from its inception through 2005, peaking in 2011–2012 before experiencing a decline (Figure 3B and Supplementary Tables B1–B13).

### 3.6 Distribution of HMIS used in public health emergency preparedness

The use of all HMIS (those that contained tacit and explicit knowledge) was initially limited to higher-income settings but, with time, spread out to LIC and LMIC. Moreover, fewer HMIS that contained tacit content were available in lower-income settings. The proportion of articles in HMIS that contained tacit knowledge that were developed in 1960, 1994, 1995, 1997, 2003, 2004, and 2017, and used in LIC and LMIC, were 2, 11, 14, 31, 16, 29, and 14%, and 16, 23, 30, 46, 33, 29, and 37%, respectively.

**Figure 4:**
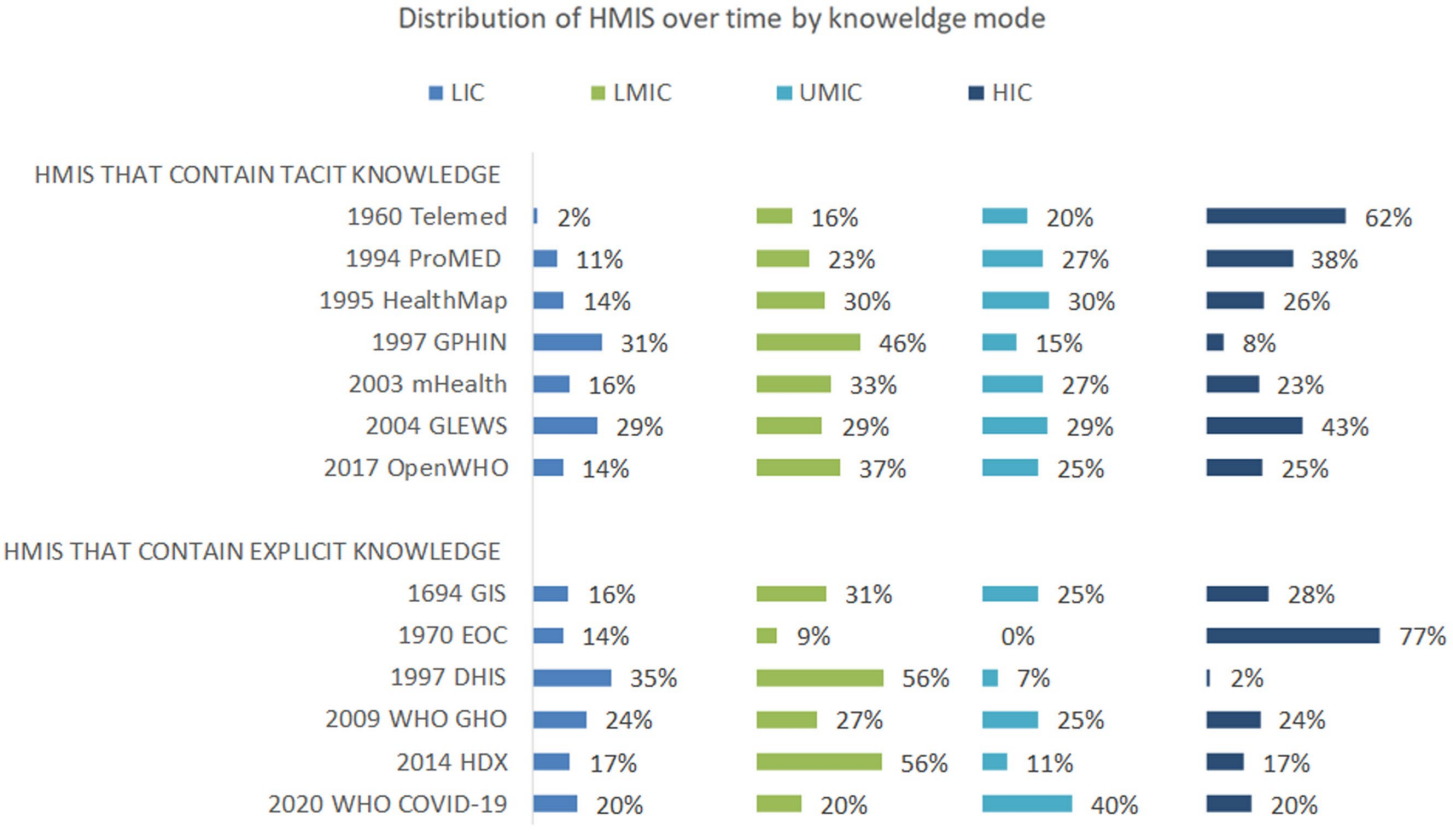
Distribution, year of development and World Bank Income classification status of countries where HMIS containing tacit knowledge (upper panel) and HMIS containing explicit knowledge (lower pane) were used in the reviewed articles. HMIS were categorized by knowledge mode into two: HMIS that contain tacit knowledge, i.e. those that describe an aspect of reasoning knowledge that has not been articulated (upper panel) and HMIS that contain explicit knowledge, i.e., those that represent knowledge that has been articulated and formalized in documents or databases (lower panel). Countries where HMIS were used was obtained from each article and categorized based on 2022 World Bank income classification status. The distribution of HMIS containing tacit knowledge (upper panel) and HMIS containing explicit knowledge (lower panel) was initially limited to higher income settings but with time spread out to include all countries regardless of income status. DHIS, District Health Information System; EOC, Emergency Operations Center; GIS, Geographical Information System; GLEWS, Global Early Warning System; GPHIN, Global Public Health Intelligence Network; HDX, Humanitarian Data Exchange; HealthMap, an automated electronic information system for monitoring, organizing, and visualizing reports of global disease outbreaks; mHealth-Mobile health applications; OpenWHO, World Health Organization’s online learning platform; ProMED, ProMED mail: Program for Monitoring Emerging Diseases; Telemed, telemedicine platforms; WHO COVID-19, World Health Organization COVID-19 dashboard; WHO GHO, World Health Organization Global Health Observatory. LIC-low-income countries, LMIC- low middle income countries, UMIC- upper middle-income countries, HIC- high middle-income countries as per World Bank Classification.

The proportion of articles on HMIS that contained explicit knowledge that were developed in 1960, 1994, 1995, 1997, 2003, 2004, and 2017, and used in LIC and LMIC, were 16, 14, 35, 24, 17, and 20%, and 31, 9, 56, 27, 56, and 20%, respectively (Figure 4 and Supplementary Tables D1–D13).

### 3.7 Knowledge attributes of all HMIS evaluated in this study

**Figure 5:**
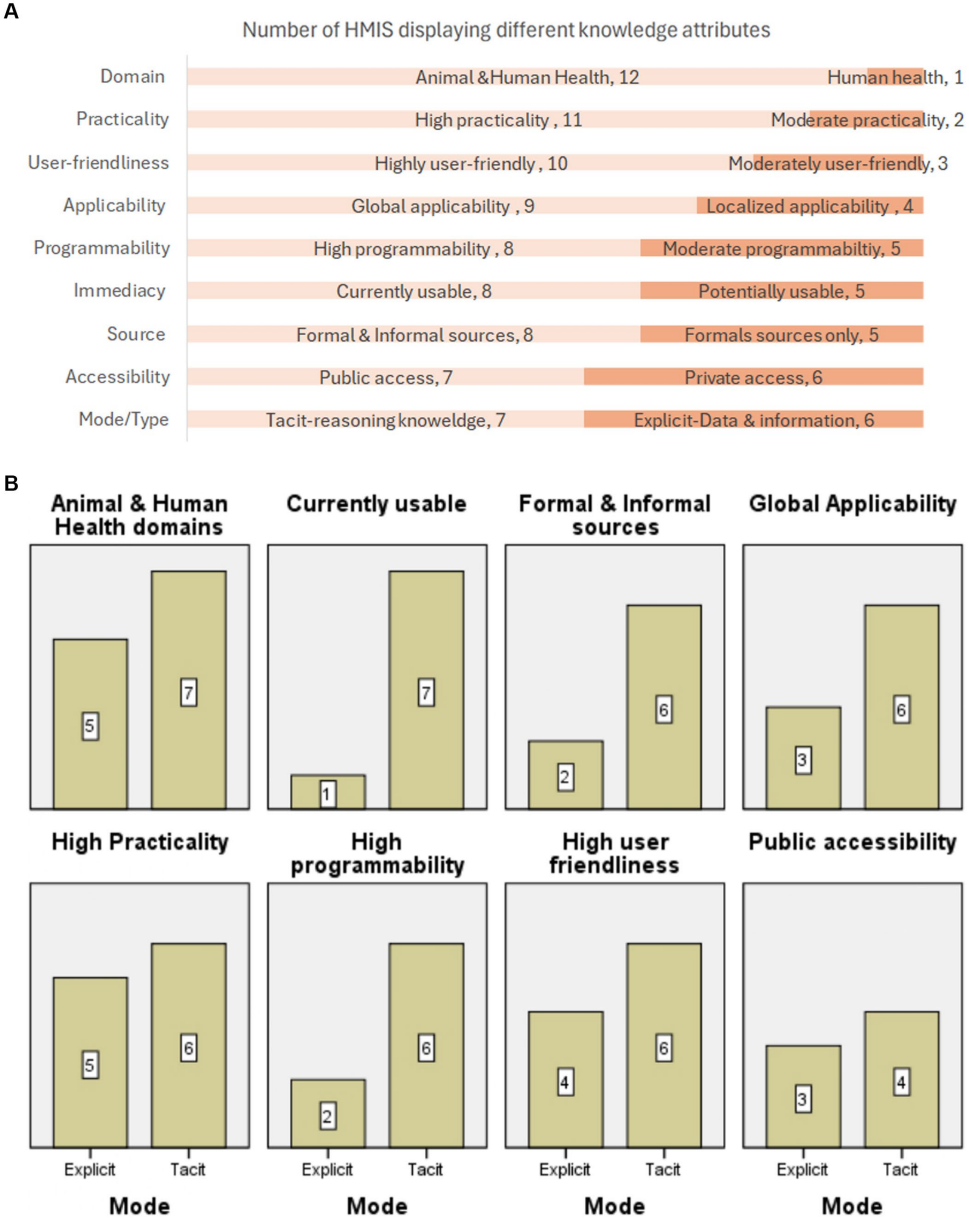
(A) Number of HMIS used in health emergencies exhibiting different knowledge attributes. HMIS were characterised using the following attributes: (1) Domain: Animal health or human health or both. (2) Practicality: High (contains relevant information that is pertinent to the problem at hand) or moderate (partially contains relevant information that is pertinent to the problem at hand), low (contains very little relevant information that is pertinent to the problem). (3) User-friendliness: High (easy to navigate), moderate (slightly challenging to navigate), low (very challenging to navigate). (4) Applicability: Localized (only used within a country or countries for a specific purpose) or global (universally usable in routine and frequent circumstances). (5) Programmability: High (very easy to transfer for use), moderate (slightly challenging to transfer) to low (very challenging to transfer). (6) Immediacy: Potentially usable (latent) or currently usable. (7) Source: Formal (established sources) or informal (expert opinion, media, public etc) or both formal and informal sources. (8) Accessibility: Private (closed source-accessible to specific processors) & public (open source-accessible to any processor). (9) Mode/Type. Mode: Tacit (knowledge that has not been articulated and is contained in the heads of experts) & explicit (knowledge that has been articulated and formalized in documents or databases. Type: Data and information (descriptive) & reasoning knowledge (information that can be acted upon). Almost all HMIS reviewed covered both animal and human health domains (12/13) and were highly practical (11/13). More than three quarters were user-friendly (10/13), close to two thirds were global (9/13), highly programmable (8/13), currently actionable (8/13) and obtained information from both formal and informal sources (8/13). Slightly more than half of the HMIS evaluated were open to the public (7/13) and contained tacit knowledge (7/13). **(B)** Knowledge attributes of HMIS that contain tacit and explicit knowledge. Number of HMIS containing tacit knowledge and HMIS containing explicit knowledge used in health emergencies demonstrating select knowledge attributes. HMIS containing tacit knowledge were compared to HMIS containing explicit knowledge using the following knowledge attributes, domain, immediacy, source, applicability, practicality, programmability, user-friendliness and accessibility. Compared to HMIS that contain explicit knowledge, more HMIS containing tacit knowledge covered both animal and human domains, were currently usable, obtained information from formal and informal sources, were globally applicable, highly programmable, highly user-friendly and publicly accessible. But, both HMIS that contain tacit knowledge and HMIS that contain explicit knowledge used in emergencies were equally practical and accessible.

The majority of HMIS reviewed covered both animal and human health domains (12/13) and were highly practical (11/13). More than three quarters were user-friendly (10/13), close to two-thirds were global (9/13), highly programmable (8/13), currently actionable (8/13), and obtained information from both formal and informal sources (8/13). Over half of the HMIS evaluated were open to the public (7/13) and contained tacit knowledge (7/13) (Figure 5A and Supplementary Tables C1–C9).

### 3.8 Knowledge attributes of tacit and explicit knowledge containing HMIS

Compared to HMIS that contained explicit knowledge, more HMIS containing tacit knowledge covered both animal and human domains (7 vs. 5), were currently usable (7 vs. 1), obtained information from formal and informal sources (6 vs. 2), was universally applicable (6 vs. 3), highly programmable (6 vs. 2), and user-friendly (6 vs. 4). Nonetheless, both HMIS that contained tacit knowledge and HMIS that contained explicit knowledge were equally highly practical (6 and 5, respectively) and accessible (4 vs. 3). Only half of all HMIS reviewed were open to the public (Figure 5B and Supplementary Tables C1–C9).

### 3.9 Number of mentions for each knowledge attribute in all the articles reviewed

**Figure 6:**
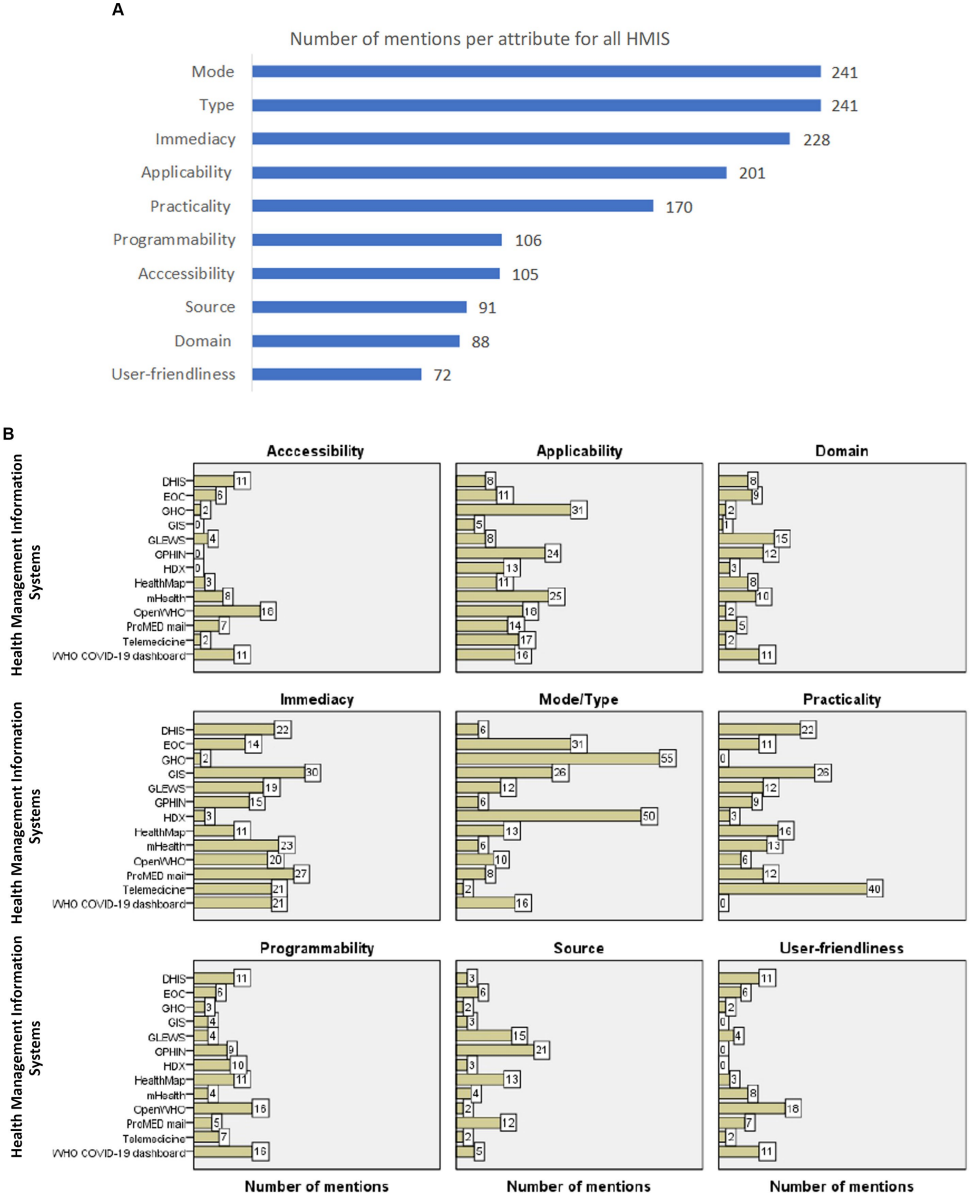
(A) The number of mentions per knowledge attribute for all HMIS evaluated in the study. The figure lists the number of mentions for each knowledge attribute in all the articles reviewed for all HMIS. Excluding mode and type, the highest number of mentions were for immediacy, applicability, and practicality. **(B)** The proportion of mentions of each knowledge attribute per HMIS. The proportion of mentions per knowledge attribute for each HMIS evaluated in the study. The proportion of mentions per attribute for each HMIS was computed as a fraction of the total number of mentions for all attributes for that specific HMIS. The top 5 HMIS with the highest mentions for accessibility were on OpenWHO (*n* = 18), WHO-COVID 19 dashboard (*n*= 11),DHIS (*n* = 11), mHealth (*n* = 8), and ProMed mail (*n* = 7); for applicability were on WHO GHO (31), mHealth (25), GPHIN (*n* = 24), OpenWHO (*n* = 18) and Telemedicine (*n* = 17); for domain were GLEWS (*n* = 15), GPHIN (*n* = 12), WHO COVID-19 dashboard (*n* = 11) mHealth (*n* = 10) and EOC (*n* = 9); and for immediacy were on GIS (*n* = 30), ProMed mail (*n* = 27), mHealth (*n* = 23), DHIS (*n* = 22) and Telemedicine platforms (*n* = 21) and WHO COVID-19 dashboard (*n* = 21). The top 5 HMIS with the highest mentions for mode/type were on GHO (*n* = 55), HDX (*n* = 50), EOC (*n* = 31), GIS (*n* = 26) and WHO-COVID-19 dashboard (*n* = 16); for practicality were on Telemedicine platforms (*n* = 40), GIS (*n* = 26), DHIS (*n* = 22), HealthMap (16) and mHealth (*n* = 13); for programmability were on WHO COVID-19 dashboard (n=16), OpenWHO (*n* = 16), HealthMap (*n* = 11), DHIS (*n* = 11) and HDX (*n* = 10); for source were on GPHIN (*n* = 21), GLEWS (*n* = 15), HealthMap (*n* = 13), ProMed mail (*n* = 12), and EOC (*n* = 6), and for user-friendliness were on OpenWHO (*n* = 18), DHIS (*n* = 11), WHO COVID-19 dashboard (*n* = 11), mHealth (*n* = 8) and ProMed mail (*n* = 7). DHIS, District Health Information System; EOC, Emergency Operations Center; GIS, Geographical Information System; GLEWS, Global Early Warning System; GPHIN, Global Public Health Intelligence Network; HDX, Humanitarian Data Exchange; HealthMap, an automated electronic information system for monitoring, organizing, and visualizing reports of global disease outbreaks; mHealth-Mobile health applications; OpenWHO, World Health Organization’s online learning platform; ProMED, ProMED mail: Program for Monitoring Emerging Diseases; Telemed, telemedicine platforms; WHO COVID-19, World Health Organization COVID-19 dashboard; WHO GHO, World Health Organization Global Health Observatory. programmability were on the WHO COVID-19 dashboard (*n* = 16), OpenWHO (*n* = 16), HealthMap (*n* = 11), DHIS (*n* = 11), and HDX (*n* = 10); for accessibility were on HealthMap (16), HDX (*n* = 13), GLEWS (*n* = 12), OpenWHO (*n* = 10), and ProMed mail (*n* = 10); for source were on GPHIN (*n* = 21), GLEWS (*n* = 15), HealthMap (*n* = 13), ProMed mail (*n* = 12), and EOC (*n* = 6); for domain were GLEWS (*n* = 15), GPHIN (*n* = 12), WHO COVID-19 dashboard (*n* = 11) mHealht (*n* = 10) and EOC (*n* = 9); and for user-friendliness was on OpenWHO (*n* = 12), DHIS (*n* = 11), WHO COVID-19 dashboard (*n* = 11), mHealth (*n* = 8), and ProMed mail (*n* = 7).

The majority of mentioned attributes for all HMIS reviewed were mode (*n* = 241), type (*n* = 241), immediacy (*n* = 228), applicability (*n* = 201), practicality (*n* = 170), programmability (*n* = 106), accessibility (*n* = 105), source (*n* = 91), domain (*n* = 88), and user-friendliness (*n* = 72) (Figure 6A and Supplementary Tables C1–C9I).

### 3.10 Number of mentions for each knowledge attribute by HMIS in all the articles reviewed

The top 5 HMIS with the highest mentions for each knowledge attribute are shown below (Figure 6B and Supplementary Tables C1–C9).

- Mode/type: WHO GHO (*n* = 55), HDX (*n* = 50), EOC (*n* = 31), GIS (*n* = 26), and WHO-COVID-19 (*n* = 16).
- Immediacy: GIS (*n* = 30), ProMED mail (*n* = 27), mHealth (*n* = 23), DHIS (*n* = 22), telemedicine (*n* = 21), and WHO COVID-19 (*n* = 21).
- Applicability: WHO GHO (31), mHealth (25), GPHIN (*n* = 24), OpenWHO (*n* = 18), and Telemedicine (*n* = 17).
- Practicality: Telemedicine (*n* = 40), GIS (*n* = 26), DHIS (*n* = 22), HealthMap (16), and mHealth (*n* = 13).
- Programmability: WHO COVID-19 (*n* = 16), OpenWHO (*n* = 16), HealthMap (*n* = 11), DHIS (*n* = 11), and HDX (*n* = 10).
- Accessibility: OpenWHO (*n* = 18), WHO-COVID 19 dashboard (*n* = 11), DHIS (*n* = 11), mHealth (*n* = 8), ProMed mail (*n* = 7) and EOC (*n* = 6).
- Source: GPHIN (*n* = 21), GLEWS (*n* = 15), HealthMap (*n* = 13), ProMED mail (*n* = 12), and EOC (*n* = 6).
- Domain: GLEWS (*n* = 15), GPHIN (*n* = 12), WHO COVID-19 (*n* = 11), mHealth (*n* = 10), and EOC (*n* = 9).
- User-friendliness: OpenWHO (*n* = 18), DHIS (*n* = 11), WHO COVID-19 (*n* = 11), mHealth (*n* = 8), and ProMED mail (*n* = 7).

## 4 Discussion

This review described the knowledge attributes of HMIS used in health emergencies to inform the development of a NoK platform for knowledge continuity during and following health emergencies. Study findings referencing different sections of the results, are placed at the beginning of each paragraph or section, and recommendations stemming from each study finding are highlighted at the end of each paragraph in italics and summarized in Table 2.

**Table 2:** Optimum characteristics of a HMIS for public health emergency preparedness

Knowledge management process (18)	*Description/Attribute*	*Lessons*
Codification or development	*Creating accessible and usable knowledge sources.*	*Lesson 1: IMS should strive to continuously capture both articulated and unarticulated modes of knowledge to accommodate for the volatile nature of knowledge.* *Lesson 2: IMS should address the current needs of emergency response personnel at the time of development while complementing existing systems.*
Diffusion	*Knowledge spread over time*	*Lesson 3: IMS should employ the use of low-cost low band-width ubiquitous interoperable technologies to facilitate equitable universal access to tacit and explicit knowledge.*
Knowledge attributes’ selection	*Knowledge characteristics.*	*IMS should consider commendable characteristics of existing IMS in their development categorized by knowledge attributes in lessons 4 to 13*
	Highly cited attributes	*Lesson 4: Emergency health personnel are interested in established knowledge gained from experience that has led to favourable outcomes in other health emergencies that could be put to immediate use*.
	Mode/Type	*Lesson51: IMS should advance beyond availing of descriptive information to provide universal information upon which a user can initiate an action*
	Immediacy	*Lesson 6: IMS should avail only the necessary information to public health officials to support relevant action in either an on-going emergency or to plan for a future emergency*
	Applicability	*Lesson 7: IMS should endeavor to make the best use of locally held information with potentially global applicability.*
	Practicality	*Lesson 8: IMS should capture both tacit and explicit knowledge for use during a protracted emergency and reuse during future emergencies for knowledge continuity.*
	Source	*Lesson 9: IMS should obtain knowledge from both formal established sources and from informal sources such as the ‘know-how’ preserved in in the heads of their emergency responders.*
	Domain	*Lesson 10: Protecting both animal and human populations requires the concurrent use of both animal and human health systems*
	Accessibility	*Lesson 11: IMS should facilitate cross-sectoral, cross-functional learning that transcends national borders during and following public health emergencies by availing only the relevant aspects of privately held information to all emergency responders to support emergency response*
	Programmability	*Lesson 12: IMS product owners should strive to meet user’s programmability needs by ensuring the IMS can be used on different devices and in offline or online formats or customized to need*
	User friendliness	*Lesson 13: IMS owners should involve users from the development process, during the piloting of an IMS and routinely review users experience to ensure that the IMS is continuously updated to address users changing needs.*

Source of lessons: Authors.

### 4.1 Results in context

#### 4.1.1 Development of HMIS

Health Management Information Systems have been in use for a very long time, and generally, HMIS that contain explicit knowledge were developed before HMIS that contain tacit knowledge (section 3.3). The use of information in health fields dates back to 400 BC, implying that human beings have always evaluated and organized information about themselves and their environment and found ways to preserve and use knowledge (27). HMIS containing tacit knowledge may have been developed later to capture knowledge yet to be articulated (15). *Lesson 1: HMIS should strive to continuously capture both articulated and unarticulated knowledge to accommodate the volatile nature of knowledge* (32).

More HMIS were developed over time, as reflected by the number of articles published over time (sections 3.5 and 3.6), possibly to address different knowledge needs and in response to the availability of new technologies.

The development of HMIS to cater to different needs has been highlighted in several publications. Many articles followed the inception and expanding coverage of EOC, which rapidly share outbreak reports of emerging infectious diseases (33). The increased use of GLEWS reflects the need to integrate alert and response mechanisms from three institutions to manage zoonotic diseases (34). Many publications emerged after the inauguration of the WHO GHO, which was developed to share data on WHO priority health topics (35, 36). Similarly, HDX was developed to facilitate data sharing across crises and organizations (37), OpenWHO was designed for online learning (38), and the WHO COVID-19 dashboard was developed to present COVID-19 data on cases, deaths, and vaccinations (6). GPHIN, originally conceived to capture informal sources of information (39), experienced a decline in activity since 2010 due to a shift in priorities (40). Telemedicine, first used in the 1960s to provide remote health services for isolated populations (41), experienced an initial decrease in use due to costs followed by a surge in use during the COVID-19 pandemic as a risk-reduction strategy, a trend mirrored by the significant increase in related publications from 2020 onward (42).

An increase in the development of HMIS could also be linked to the availability of new technologies. An increase in the number of articles on GIS, developed to illustrate the spatial distribution of disease, could be explained by the increasing availability of device-agnostic, affordable GIS technologies (43). The scale-up of DHIS could be attributed to its continuous modification to include open-source contextualized features (44), and the increase in the use of mobile applications could be attributed to the widespread availability of mobile technologies (45). HealthMap, which was developed to structure outbreak information by geography, time, and infectious disease agent (46), had several articles published following the availability of a free website in 2006, HealthMap.org (47). Similarly, several publications followed the inception and increasing coverage of ProMED mail, which was developed to address the need to conduct digital disease surveillance (48–53). *Lesson 2: HMIS should address the current needs of emergency response personnel by employing the majority of recent technologies at the time of development while complementing existing systems*.

#### 4.1.2 Distribution of HMIS

All HMIS, regardless of knowledge mode, were more common in HIC and UMIC but later spread to LIC and LMIC (section 3.6) (54). HMIS distribution could be attributed to the need for stable internet and electricity for telemedicine platforms (55). Recently developed HMIS containing tacit knowledge, including mobile technology (56) and the OpenWHO online learning platform, had an equivalent distribution across income settings (57, 58), possibly due to the spread of low-cost, interoperable, open-source, low-bandwidth technologies over time (27). More HMIS that contain explicit knowledge that require fewer technological requirements,were found in lower-income settings than HMIS that contain tacit knowledge (section 3.6). The availability of GIS technology in lower-income settings could be attributed to the wide range of equipment and skills required to use GPS/GIS technology in emergencies, from basic to state-of-the-art equipment and training, respectively (27). Similarly, EOCs, which are mainly physical or sometimes virtual coordination centers set up to manage emergencies, may not require sophisticated technologies (59). EOCs were first used in the US in 1970 (49); WHO established the use of EOCs in 2012; the first EOC in Africa was established in Uganda in 2013. Other EOCs were set up in Senegal in 2018 and in Ethiopia in 2020 (48, 50, 51, 60). DHIS is the preferred information system for developing countries (61) due to its open-source contextualized features (44). However, the limited number of HMIS that contain tacit knowledge in LIC and LMIC does not imply that less tacit knowledge is found in LMIC and LIC. There may be more tacit knowledge in the heads of emergency responders in lower-income settings who have had more experience managing health emergencies with limited resources yet to be harnessed, which is one of the primary purposes of developing the NoK platform (62, 63). *Lesson 3: HMIS should employ the use of low-cost, bandwidth, ubiquitous, interoperable technologies to collate and equitably distribute all forms of knowledge* (15).

#### 4.1.3 Knowledge attributes of HMIS used in health emergencies

##### 4.1.3.1 Highly mentioned knowledge attributes

Excluding the mode and type that characterize an HMIS, popular knowledge attributes were immediacy, applicability, and practicality (section 3.9). The immediacy of knowledge is linked to its actionability, i.e., the power to know what to do with data and information. Emergency responders can scrutinize information and data to make decisions, but moving from data to decision-making is time-consuming (15), and it may or may not lead to favorable outcomes during crises when time is of the essence. The practicality of knowledge is linked to the speed of its use, its accuracy, and a user’s satisfaction with its outcomes (32). *Lesson 4: Emergency health personnel are in need of experiential knowledge that has led to favorable outcomes in previous and ongoing health emergencies that could be put to immediate use* (32).

##### 4.1.3.2 Knowledge mode of HMIS used in health emergencies

As regards the knowledge mode/type, 7 of the 13 HMIS contained tacit knowledge (sections 3.2 and 3.7). HMIS with the highest mentions for mode/type included data-based platforms, including WHO GHO (36), HDX (64), GIS (65), WHO-COVID-19 (66), and the information-dependent EOCs (67, 68). All 5 HMIS that highly cited their knowledge mode/type were HMIS that contained explicit knowledge (section 3.10). Tacit knowledge may have not received many mentions as it intuitive, hard-to-define and experience-based knowledge (69). HMIS containing tacit knowledge encompasses reasoning knowledge that can be acted upon (15) and would be life-changing, especially in health emergencies that are prone to lower-income settings (70). Without tacit knowledge, emergency responders have limited access to actionable information (13, 15). *Lesson 5: HMIS should advance beyond availing descriptive information to providing universal information upon which a user can initiate an action* (71).

##### 4.1.3.3 Knowledge attributes of HMIS that contain tacit knowledge and HMIS that contain explicit knowledge

Eight of 13 HMIS were currently usable (an attribute of immediacy); there were more HMIS that contained tacit knowledge that was currently usable than HMIS that contained explicit knowledge (7 vs. 1) (sections 3.7 and 3.8). HMIS that had more mentions for immediacy were either currently usable [i.e., GIS (65, 72, 73), ProMED mail (74–81), telemedicine platforms (82, 83), and mHealth (84–86)] or potentially usable [DHIS (87–91) and WHO COVID-19 dashboard (66, 92–94)]. Three of the 4 HMIS that were currently usable HMIS that contained tacit knowledge, i.e., ProMED mail, telemedicine platforms, and mHealth (section 3.10). During an emergency response, public health officials are hard-pressed to make accurate and timely decisions, sometimes in an environment with too much or too little information (95). Yet the right amount of key information is imperative to make timely decisions. At such times, they need systems that can easily retrieve befitting knowledge of the current emergency (including information gained from past emergencies) (13) in a suitable format to facilitate decision-making (96). Data collection should extend beyond being a reporting requirement to support local decision-making (97). *Lesson 6: HMIS should only provide the necessary information to public health officials to support relevant action in either an ongoing emergency or to plan for a future emergency* (95).

Nine of the 13 HMIS reviewed were globally applicable. Notably, more HMIS with tacit knowledge were globally applicable than those with explicit knowledge (6 vs. 3) (Sections 3.7 and 3.8). The top five HMIS for global applicability included WHO GHO, frequently used in global disease analyses (98–102); mHealth, owing to the widespread use of mobile phones (45, 103); OpenWHO, utilized in diverse settings (104–106); GPHIN, which provides coverage for the majority of countries globally (107, 108), and telemedicine, which is increasingly available in the majority of settings via mobile apps (84, 109). All five highly cited HMIS with universal applicability were also globally applicable, four of them—mHealth, OpenWHO, GPHIN, and telemedicine—contained tacit knowledge (Section 3.10). The global coverage of the majority of HMIS denotes the ability of multiple users to access information beyond the confines of national borders (15). Knowledge generated from one health emergency, for instance, is likely to be useful in subsequent emergencies in the same or other settings (13, 14). However, some globally applicable HMIS were not publicly accessible (section 3.7). Nonetheless, locally applicable HMIS may have universal applicability. DHIS (89, 110–113), which is used in over 100 countries with a total population of 3.2 billion, could be transformed from a local private HMIS to a global publicly accessible platform through standardized processes (114). Moreover, EOCs (50, 115) can be networked (116). HMIS ‘owners’ should identify known and unknown latent (potentially usable) knowledge, evaluate its potential applicability or usability, and find out how to minimize costs or efforts of making it currently usable, e.g., by linking sources of knowledge that were previously unlinked (15). *Lesson 7: HMIS should endeavor to make the best use of locally held information with potentially global applicability*.

Eleven of 13 HMIS reviewed in this study were highly practical; both HMIS that contained tacit and HMIS that contained explicit knowledge were equally highly practical (6 vs. 5) (section 3.7). Four of the five HMIS ranked among the top five that highly cited practicality contained information that can be used at an operational, managerial, or strategic level, i.e., they were highly practical (15), they included telemedicine (55, 117–124), mHealth (125–129), HealthMap (46), and GIS (27, 43, 130–134). DHIS had a limited focus on examining findings and taking action-based decisions (110, 111, 135–137). Three of the 4 HMIS that were highly practical were HMIS that contained tacit knowledge, i.e., telemedicine, mHealth, and HealthMap (section 3.10). Advocating for more HMIS that contain tacit knowledge does not obviate the need for HMIS that contain explicit knowledge since cross-organizational and multi-agency data, information, and knowledge are used to support critical decision-making during public health events (138) by several emergency responders who converge, sometimes for the first time, during a public health emergency (138). *Lesson 8: HMIS should capture both tacit and explicit knowledge for use during a protracted emergency and reuse during future emergencies for knowledge continuity*.

Eight of 13 HMIS obtained information from formal and informal sources; there were more HMIS that contained tacit knowledge that obtained information from both formal and informal sources than HMIS that contained explicit knowledge (6 vs. 2) (section 3.3, 3.7, and 3.8). The highest mentions for sources were GPHIN, which gathers information from news media (39), GLEWS, which relies on institutional and non-official information (139, 140), EOC, which obtains information from varied sources (14), HealthMap, which combines both news media sources and government information (46, 141), and ProMED mail, which relies on user submissions (81, 142). All 5 HMIS with the highest mentions for source obtained information from both formal and informal sources; four were HMIS containing tacit knowledge, i.e., GLEWS, GPHIN, HealthMap, and ProMED mail (section 3.10). Like HMIS, which contains explicit knowledge, HMIS, which contains tacit knowledge, obtains information from established sources and complements such information with information from other sources like new reports, expert opinions, and individual submissions to expand the scope of the content. Therefore, tacit and explicit knowledge should be seen as a spectrum rather than definite points, with all forms of knowledge being a little of a mix of both modes (69, 143, 144). It may even be difficult to distinguish the two modes in some settings (145). Formal knowledge sources advance innovation’s techno-economic aspects, which are decontextualized and vertically disseminated. However, such innovations may not readily adapt to all contexts, leading to know-do gaps (146, 147). Moreover, only information-based interventions, i.e., those that rely on ‘rational’ decision-making, often overlook the complexities of choice (or inaction) during emergencies (148, 149). Informal sources of knowledge, which contain mainly tacit knowledge, are the most valuable source of knowledge that results in innovations (69), and advanced social innovations that are relevant to bridging the know-how gap (147). Both technological and social innovations are essential to protecting the health of populations during public health emergencies, as they are both relevant to innovation performance (150). *Lesson 9: HMIS should obtain knowledge from both formally established sources and informal sources, such as the ‘know-how’ preserved in the heads of their emergency responders*.

Twelve of the 13 HMIS reviewed covered both animal and human health domains, with more HMIS containing tacit knowledge covering both animal and human domains than HMIS that contain explicit knowledge (7 vs. 5) (sections 3.7 and 3.8). HMIS, which had the highest mentions for the domain, were GPHIN (107, 108) and EOC (33), which have a broad scope. GLEWS focuses on one health (151), mHealth, that has been used in several health systems’ programs (109, 152–155), and WHO COVID-19 exists solely to share data related to COVID-19 burden and vaccinations (66). Four of the five HMIS that had the highest mentions for the domain were on both animal and human health; three of them were HMIS that contained tacit knowledge, i.e., GPHIN, GLEWS, and mHealth (section 3.10). The subject domain of most HMIS acknowledges the interlinkages and interdependencies of human, animal, plant, and environmental health (156). An encouraging trait since the majority of emerging diseases occur at the human-animal interface. A one-health platform improves the effectiveness of surveillance and control of infectious diseases. Data in such platforms is not necessarily merged but is collaboratively used to address complex health challenges in both sectors (157, 158). *Lesson 10: Protecting both animal and human populations requires the concurrent use of both animal and human health systems*.

About half (7 of 13) of the HMIS evaluated were public; both HMIS that contained tacit knowledge and HMIS that contained explicit knowledge were equally accessible (4 vs. 3) (section 3.7 and 3.8). The top 5 mentions for accessibility were for HMIS that were open source [i.e., OpenWHO (38, 58, 166, 167), WHO-COVID 19 dashboard (6), and ProMEd mail (76, 77, 80)] and closed source [i.e., DHIS (61, 113, 114) and mHealth (85, 113, 154)]. Three of the five HMIS with the highest mentions for accessibility were HMIS that contained tacit knowledge, i.e., OpenWHO, mHealth and ProMed mail (section 3.10). With only half of the HMIS being open source, emergency responders may not be privy to information that is relevant to their day-to-day activities. Nevertheless, information should be easily accessed as needed (168). There may be challenges regarding data security and privacy when sharing data (169). Because outbreaks transcend national borders, coordinated multicounty efforts are required to address them. A country can share only lessons learned and best practices from a health emergency with other countries to limit the possibility of sharing confidential information (170). *Lesson 11: HMIS should facilitate cross-sectoral, cross-functional learning that transcends national borders during and following public health emergencies by availing only the relevant aspects of privately held information to all emergency responders to support emergency responses*.

Eight of 13 HMIS were highly programmable/transferable; more HMIS contained tacit knowledge that was highly programmable than HMIS that contained explicit knowledge (6 vs. 2) (sections 3.7 and 3.8). The most common mentions for programmability were on HMIS, which were highly programmable [i.e., WHO COVID-19, which is downloadable and usable on phones (66, 92, 171), and OpenWHO, which can be used with low-bandwidth technologies and has both offline and online formats (58, 166, 172, 173); HealthMap that can be used on mobile phones (174); DHIS that is customizable (175)], or moderately programmable [i.e., HDX for its limited transferability (176, 177)]. Three of the 4 HMIS that were highly programmable were HMIS that contained tacit knowledge, including OpenWHO, HealthMap, and HealthMap (section 3.10). The transferability of knowledge is related to its ease of use and accessibility. It is also related to the shareability of knowledge. However, highly programmable HMIS may or may not have been open source. Users should be able to access data on different devices in offline or online formats (36) in areas with limited electricity supply or internet access (55) or to modify or customize the HMIS to their changing needs (175). *Lesson 12: HMIS product owners should strive to meet users’ programmability needs by ensuring that the HMIS can be used on different devices and in offline or online formats*.

Ten of the 13 HMIS reviewed in this study were user-friendly; there were more HMIS that contained tacit knowledge that was highly user-friendly than HMIS that contained explicit knowledge (6 vs. 4) (sections 3.7 and 3.8). HMIS that were highly cited for user-friendliness were all noted for their high user-friendliness, including OpenWHO, which has self-paced courses in various languages (104–106, 173), DHIS (61, 113), and mHealth applications (84, 125, 178) for their ease of use; the WHO COVID-19 dashboard that was ranked among 2020’s top 1,000 most popular websites worldwide (93, 171), or moderately user-friendly, i.e., ProMEd mail for its information overload (81, 179, 180). Three of the 4 HMIS that were noted for their high user-friendliness were HMIS that contained tacit knowledge, including OpenWHO, mHealth, and ProMED mail (section 3.10). An ideal HMIS should strike the right balance between too little and too much information (181). HMIS should facilitate easy access and retrieval of meaningful information for immediate, relevant action (182, 183). HMIS weaknesses were mainly linked to the inability to provide meaningful information and rarely their ease of use. Fewer reports of suboptimal user-unfriendliness could be attributed to the majority of software being primarily developed for a user’s convenience (184). HMIS users and their needs are likely to change throughout the development process of an HMIS and beyond. HMIS ‘owners’ should involve users during the inception and development of a HMIS to ensure an HMIS aligns with users’ needs and provides value beyond convenience (185) and throughout the life of the HMIS to ensure that the HMIS remains both highly usable and utile (186). *Lesson 13: HMIS owners should involve users in the development process, during the piloting of an HMIS and routinely review users’ experience to ensure that the HMIS is continuously updated to address users’ changing needs*.

### 4.2 Strengths of the evidence

A balanced distribution of HMIS contained tacit and explicit knowledge, and the number of articles selected for HMIS of both knowledge modes permitted a balanced comparison (sections 3.2 and 3.4). Additionally, articles were retrieved from two complementary databases that have a high recall ability (187) (section 2.3). Moreover, standardized definitions were used to evaluate all HMIS (15) (section 2.4).

### 4.3 Limitations

#### 4.3.1 Limitations of the evidence

First, only 13 HMIS were evaluated in this review (section 3.2), including more HMIS, e.g., those in Supplementary Table E and those used in the WHO European region (188), may have enriched the study findings. However, we selected HMIS that could be representative of other HMIS that were not evaluated in this study. For instance, mHealth applications could represent communication platforms like WhatsApp, Telegram, or custom-built communication platforms for health workers; DHIS could represent electronic health records, and OpenWHO could represent other online training platforms. Second, only nine knowledge attributes were used to characterize HMIS (section 3.7). However, only select knowledge attributes are required to inform the development of a knowledge management system (32). Third, few articles were selected per HMIS, limiting the extraction of information related to knowledge attributes (section 3.1). Other information related to HMIS used in health emergency preparedness can be found only in gray literature or obtained by evaluating user experience. Nonetheless, some of this information was documented in some of the articles reviewed in this study. Fourth, the review did not focus on HMIS which were no longer in use at the time of the study (section 2.4). Given the broad parameters already set, this is outside the scope of this study but may be the subject of future complementary research.

#### 4.3.2 Limitations of the review processes

Our approach, a scoping review, that provides the breadth rather than the depth of information on a particular topic, cannot be equated to level of evidence generated from a comprehensive systematic literature review (187, 189). A single reviewer screened and reviewed all articles from two databases (section 2.4). But our methodology aligns with the WHO recommendations for rapid appraisals (20). The search was conducted once (section 2.3), and articles or book chapters that were not available online were not analyzed; neither were corresponding authors of articles or experts consulted (section 3.1). Although we only used two databases (section 2.3), our scoping review meets the WHO criteria for rapid reviews to feasibly obtain information in a timely fashion (190). Nevertheless, the evidence generated demonstrates the strengths and weaknesses of existing HMIS in health emergencies, information that can inform the development of a NoK Platform for health emergency preparedness.

### 4.4 Implications for practice, policy, and future research

The HMIS that contain tacit knowledge have more favorable attributes than those that contain explicit knowledge, but they may not be available to all emergency responders globally, a distribution that may change as newer low-cost technologies are available. An ideal HMIS should continuously capture a broad range of untapped experiential knowledge that is relevant to emergency response personnel in a HMIS that is user-friendly and accessible to people in all geographical settings. The HMIS, which should address the current needs of emergency response personnel, must be actionable, practical, and easily transferrable. Such á HMIS can guarantee that informal knowledge is articulated, actionable knowledge is publicized, mutually beneficial connections between human and animal health domains are made, and localized knowledge transcends national borders. Global strategies should facilitate the development of national policies that facilitate knowledge sharing akin to that recommended by the WHO Benchmarks for IHR (191) and the WHO guidance for COVID-19 after-action reviews that recommend multicounty AARs (170) and EOC networks (192). These implications, summarized in Table 2, will inform the development of the NoK platform to facilitate the preservation and continuity of tacit knowledge from public health emergencies in line with WHO strategies. Future research should investigate the impact of such a platform on public health emergency management.

## Data Availability

The original contributions presented in the study are included in the article/Supplementary material, further inquiries can be directed to the corresponding author.

## Supplementary material

SUPPLEMENTARY DATA SHEET 1 Supplementary Tables A1 and A2.

SUPPLEMENTARY DATA SHEET 2 Supplementary Tables B1 to B13.

SUPPLEMENTARY DATA SHEET 3 Supplementary Tables C1 to C9.

SUPPLEMENTARY DATA SHEET 4 Supplementary Tables D1 to D13.

SUPPLEMENTARY DATA SHEET 5 Supplementary Table E.
