# Supplementary material for: Knowledge attributes of public health management information systems used in health emergencies: a scoping review
Source: Front Public Health. 2025 Mar 20;12:1458867. doi: 10.3389/fpubh.2024.1458867 (PMC11969037; doi:10.3389/fpubh.2024.1458867)
Supplement: SUPPLEMENTARY DATA SHEET 1 — Supplementary Tables A1 and A2. [file Data_Sheet_1.zip › SupplementaryTables_A1_A2_LiterarySourcesPerDatabase/SupplementaryTable_A1_W_Science.docx]

**Supplementary table A1: Web of Science Search Results**

| **HMIS** | **Date** | **Search terms** | **Number reviewed** | **No. Screened** | **Not retrieved** | **No. excluded** | **Duplicates** | **Not focused on IMS** | **No information on knowledge attributes** | **No. retained** |
| --- | --- | --- | --- | --- | --- | --- | --- | --- | --- | --- |
| DHIS2 | 22/02/2024 | District Health Information Software 2 and Public Health Emergencies  Keywords: District Health Information Software 2 | 4366 | 20 | 1 | 14 | 2 | 8 | 4 | 5 |
| Open WHO | 22/02/2024 | Topics: OpenWHO and Health emergencies  Keywords: OpenWHO | 5533 | 20 | 0 | 15 | 11 | 0 | 4 | 5 |
| EOC | 22/02/2024 | Topics: emergency operation centers  Keywords: emergency operation centers | 3170 | 20 | 0 | 14 | 2 | 10 | 2 | 6 |
| GPHIN | 06/03/2024 | Topics: Global Public Health Intelligence Network (GPHIN)  Keywords: Global Public Health Intelligence Network (GPHIN) | 24 | 24 | 1 | 16 | 4 | 2 | 10 | 7 |
| mHealth | 02/03/2024 | Topics: mHealth and Public Health Emergencies  Keywords: mHealth | 234 | 21 | 1 | 10 | 6 | 0 | 4 | 10 |
| HealthMap | 02/03/2024 | Topics: HealthMap and Public Health Emergencies  Keywords: HealthMap | 125 | 21 | 0 | 15 | 4 | 3 | 8 | 6 |
| ProMed mail | 02/03/2024 | Topics: ProMed mail and Public Health Emergencies  Keywords: ProMed mail | 107 | 21 | 0 | 14 | 5 | 5 | 4 | 7 |
| Telemed | 02/03/2024 | Topics: Telemedicine/Telehealth and Public Health Emergencies  Keywords: Telemedicine/Telehealth | 144 | 20 | 3 | 12 | 5 | 0 | 5 | 7 |
| WHO COVID-19 | 01/04/2024 | Topics: World Health Organization COVID-19 dashboard  Keywords: World Health Organization COVID-19 dashboard | 85 | 20 | 0 | 20 | 2 | 15 | 3 | 0 |
| GLEWS | 01/04/2024 | Topics: Global Early Warning System  Keywords: Global Early Warning System | 4 | 4 | 1 | 0 | 0 | 0 | 0 | 3 |
| HDX | 01/04/2024 | Topic: Humanitarian Data Exchange  Keywords: Humanitarian Data Exchange | 226 | 20 | 0 | 6 | 0 | 0 | 6 | 14 |
| WHO GHO | 01/04/2024 | Topics: World Health Organization Global Health Observatory and Public Health Emergencies  Keywords: WHO Global Health Observatory | 95 | 38 | 2 | 4 | 1 | 1 | 2 | 32 |
| GIS | 01/04/2024 | Topics: World Health Organization Global Health Observatory and Public Health Emergencies  Keywords: WHO Global Health Observatory | 348 | 20 | 1 | 2 | 0 | 0 | 2 | 17 |
| **All IMS** |  |  | 14461 | 269 | 10 | 142 | 42 | 44 | 56 | 119 |

GIS-Geographical Information Systems, Telemedicine-Telemedicine platforms, EOC-Emergency Operations Centers, ProMed- ProMed mail: Program for Monitoring Emerging Diseases, DHIS-District Health Information System, GPHIN-Global Program, Global Public Health Intelligence Network, mHealth-Mobile health applications, GLEWS-Global Early Warning System, WHO GHO- World Health Organization Global Health Observatory, HDX- Humanitarian Data Exchange, WHO COVID-19- World Health O
