# Supplementary material for: Knowledge attributes of public health management information systems used in health emergencies: a scoping review
Source: Front Public Health. 2025 Mar 20;12:1458867. doi: 10.3389/fpubh.2024.1458867 (PMC11969037; doi:10.3389/fpubh.2024.1458867)
Supplement: SUPPLEMENTARY DATA SHEET 1 — Supplementary Tables A1 and A2. [file Data_Sheet_1.zip › SupplementaryTables_A1_A2_LiterarySourcesPerDatabase/SupplementaryTable_A2_G_Scholar.docx]

**Supplementary table A2: Search results from Google scholar**

| **Google scholar search terms** | **Number reviewed** | **Duplicates** | **Not retrieved** | **No. excluded** | **Not focused on IMS** | **No information on knowledge attributes** | **Number retained** | **Hand-search/other sources** | **Total** |
| --- | --- | --- | --- | --- | --- | --- | --- | --- | --- |
| district health information system 2 | 20 | 0 | 0 | 6 | 0 | 6 | 14 | 6 | 20 |
| Emergency Operations Center (EOC) Network Systems | 20 | 0 | 0 | 9 | 2 | 7 | 11 | 2 | 13 |
| GIS (Geographical Information Systems) health emergencies | 20 | 0 | 5 | 4 | 1 | 3 | 11 | 4 | 15 |
| Global Early Warning System (GLEWS) | 20 | 0 | 0 | 12 | 5 | 7 | 8 | 3 | 11 |
| Global Public Health Intelligence Network | 20 | 0 | 0 | 15 | 12 | 3 | 5 | 0 | 5 |
| HealthMap | 20 | 0 | 0 | 11 | 11 | 0 | 9 | 0 | 9 |
| Humanitarian Data Exchange (HDX) | 20 | 0 | 0 | 12 | 1 | 11 | 8 | 0 | 8 |
| Mobile Health (mHealth) Applications | 20 | 0 | 0 | 6 | 0 | 6 | 14 | 5 | 19 |
| ProMED-mail | 20 | 1 | 0 | 3 | 0 | 3 | 16 | 0 | 16 |
| Telemedicine Platforms in public health emergencies | 20 | 0 | 0 | 8 | 0 | 8 | 12 | 4 | 16 |
| WHO Global Health Observatory | 20 | 0 | 0 | 18 | 7 | 11 | 2 | 0 | 2 |
| world health organization COVID-19 dashboard | 20 | 0 | 0 | 15 | 12 | 3 | 5 | 1 | 6 |
| Open WHO in health emergencies | 20 | 0 | 0 | 6 | 2 | 4 | 14 | 2 | 16 |
| **All HMIS** | 260 | 1 | 5 | 125 | 53 | 72 | 129 | 27 | 156 |
