# Supplementary material for: Knowledge attributes of public health management information systems used in health emergencies: a scoping review
Source: Front Public Health. 2025 Mar 20;12:1458867. doi: 10.3389/fpubh.2024.1458867 (PMC11969037; doi:10.3389/fpubh.2024.1458867)
Supplement: SUPPLEMENTARY DATA SHEET 2 — Supplementary Tables B1 to B13. [file Data_Sheet_2.zip › SupplementaryTables_B1_B13_ArtcilesPerHMIS/SupplementaryTable_B1_Articles_DHIS.docx]

**Supplementary Table B1: List of articles included in the review on DHIS (25 articles)- 15 research articles, 2 review articles**

| **Author** | **Year of publication** | **Type of article** | **Purpose** |
| --- | --- | --- | --- |
| Abajebel et al (1) | 2011 | Research article | Use of DHIS in Ethiopia |
| Asaduzzaman (2) | 2024 | Research article | DHIS2 use in Ethiopia |
| Braa & Sahay (3) | 2017 | Book section | Evolution of DHIS |
| Bulage et al (4) | 2022 | Case study | Delayed outbreak detection in Uganda |
| Dehnavieh et al (5) | 2018 | Literature review & meta-synthesis | DHIS experience in 11 countries in Africa |
| DHIS2 (6) | nd | Website | General information about DHIS |
| Eggers et al (7) | 2022 | Practice article | DHIS2 use in Guinea |
| Farnham et al (8) | 2020 | Perspective article | Impact of DHIS data on African research output |
| Garrib et al (9) | 2008 | Research article | DHIS2 use in South Africa |
| Joseph et al (10) | 2022 | Research article | Malaria surveillance in Tanzania |
| Kadia et al (11) | 2023 | Research article | DHIS2 use in Southwest Cameroon |
| Kiberu et al (12) | 2014 | Research article | DHIS strengthening in Uganda |
| Kinkade (13) | 2022 | Review article | DHIS use in Sierra Leone, Sri Lanka, and Uganda |
| Lungo (14) | 2008 | Research article | DHIS use in Tanzania |
| Manoj et al (15) | 2013 | Leading article | DHIS use in Sri Lanka |
| Manya et al (16) | 2012 | Conference paper | DHIS use in Kenya |
| Ndlovu et al (17) | 2022 | Research article | DHIS use in Botswana |
| Odei-Lartey et al (18) | 2020 | Research article | DHIS use in Ghana |
| Odhiambo-Otieno (18) | 2005a | Case study | DHIS use in Kenya |
| Odhiambo-Otieno (19) | 2005b | Research article | DHIS use in Kenya |
| Oreh (20) | 2022 | Research article | DHIS use in Nigeria |
| Raeisi et al (21) | 2013 | Research article | DHIS use in Iran |
| Simba (22) | 2022 | Journal article | DHIS use in Tanzania |
| Tchoualeu et al(23) | 2021 | Research article | DHIS use in Nigeria |
| Thangasamy et al (24) | 2016 | Research article | DHIS use in Ethiopia |
| Wangdi et al (25) | 2020 | Research article | DHIS use in Solomon Islands |

**References**

1. Abajebel S, Jira C, Beyene W. Utilization of health information system at district level in jimma zone oromia regional state, South west ethiopia. Ethiop J Health Sci. 2011;21(Suppl 1):65-76.

2. Asaduzzaman M, Mekonnen Z, Rodland EK, Sahay S, Winkler AS, Gradmann C. District health information system (DHIS2) as integrated antimicrobial resistance surveillance platform: An exploratory qualitative investigation of the one health stakeholders' viewpoints in Ethiopia. INTERNATIONAL JOURNAL OF MEDICAL INFORMATICS. 2024;181.

3. Braa J, Sahay S. The DHIS2 Open Source Software Platform: Evolution Over Time and Space. 2017.

4. Bulage L, Kadobera D, Kwesiga B, Kabwama SN, Ario AR, Harris JR. Delayed outbreak detection: a wake-up call to evaluate a surveillance system. Pan Afr Med J. 2022;41(Suppl 1):1.

5. Dehnavieh R, Haghdoost A, Khosravi A, Hoseinabadi F, Rahimi H, Poursheikhali A, et al. The District Health Information System (DHIS2): A literature review and meta-synthesis of its strengths and operational challenges based on the experiences of 11 countries. Health Information Management Journal. 2018;48(2):62-75.

6. DHIS2. About DHISnd. Available from: <https://dhis2.org/about/>.

7. Eggers C, Martel L, Dismer A, Kallay R, Sayre D, Choi M, et al. Implementing a DHIS2 Ebola virus disease module during the 2021 Guinea Ebola outbreak. BMJ Glob Health. 2022;7(5).

8. Farnham A, Utzinger J, Kulinkina AV, Winkler MS. Using district health information to monitor sustainable development. Bull World Health Organ. 2020;98(1):69-71.

9. Garrib A, Stoops N, McKenzie A, Dlamini L, Govender T, Rohde J, et al. An evaluation of the District Health Information System in rural South Africa. S Afr Med J. 2008;98(7):549-52.

10. Joseph JJ, Mkali HR, Reaves EJ, Mwaipape OS, Mohamed A, Lazaro SN, et al. Improvements in malaria surveillance through the electronic Integrated Disease Surveillance and Response (eIDSR) system in mainland Tanzania, 2013–2021. Malaria Journal. 2022;21(1):321.

11. Kadia RSM, Kadia BM, Dimala CA, Collins AE. Usefulness of disease surveillance data in enhanced early warning of the cholera outbreak in Southwest Cameroon, 2018. Conflict and Health. 2023;17(1):6.

12. Kiberu VM, Matovu JKB, Makumbi F, Kyozira C, Mukooyo E, Wanyenze RK. Strengthening district-based health reporting through the district health management information software system: the Ugandan experience. BMC Medical Informatics and Decision Making. 2014;14(1):40.

13. Kinkade C, Russpatrick S, Potter R, Saebo J, Sloan M, Odongo G, et al. Extending and Strengthening Routine DHIS2 Surveillance Systems for COVID-19 Responses in Sierra Leone, Sri Lanka, and Uganda. EMERGING INFECTIOUS DISEASES. 2022;28:S42-S8.

14. Lungo JH. The reliability and usability of district health information software: case studies from Tanzania. Tanzania journal of health research. 2008;10(1):39-45.

15. Manoj S, Wijekoon A, Dharmawardhana M, Wijesooriya D, Rodrigo S, Hewapathirana R, et al. Implementation of District Health Information Software 2 (DHIS2) in Sri Lanka. Sri Lanka Journal of Bio-Medical Informatics. 2013;Vol 3:109-14.

16. Manya A, Braa J, Øverland L, Titlestad O, Mumo J, Nzioka C. National Roll out of District Health Information Software (DHIS 2) in Kenya, 2011 – Central Server and Cloud based Infrastructure2012.

17. Ndlovu K, Mauco KL, Keetile M, Kadimo K, Senyatso RY, Ntebela D, et al. Acceptance of the District Health Information System Version 2 Platform for Malaria Case-Based Surveillance By Health Care Workers in Botswana: Web-Based Survey. JMIR FORMATIVE RESEARCH. 2022;6(3).

18. Odhiambo-Otieno GW. Evaluation of existing district health management information systems a case study of the district health systems in Kenya. Int J Med Inform. 2005;74(9):733-44.

19. Odhiambo-Otieno GW. Evaluation criteria for district health management information systems: lessons from the Ministry of Health, Kenya. International Journal of Medical Informatics. 2005;74(1):31-8.

20. Oreh A, Bozegha T, Ihimekpen A, Biyama F, Irechukwu C, Aliu S, et al. Effect of the COVID-19 pandemic on blood donations and transfusions in Nigeria - A multi-facility study of 34 tertiary hospitals. NIGERIAN JOURNAL OF CLINICAL PRACTICE. 2022;25(6):786-93.

21. Raeisi AR, Saghaeiannejad S, Karimi S, Ehteshami A, Kasaei M. District health information system assessment: a case study in iran. Acta Inform Med. 2013;21(1):30-5.

22. Simba D, Sukums F, Kumalija C, Asiimwe SE, Pothepragada SK, Githendu PW. Perceived Usefulness, Competency, and Associated Factors in Using District Health Information System Data Among District Health Managers in Tanzania: Cross-sectional Study. JMIR FORMATIVE RESEARCH. 2022;6(5).

23. Tchoualeu DD, Elmousaad HE, Osadebe LU, Adegoke OJ, Nnadi C, Haladu SA, et al. Use of a district health information system 2 routine immunization dashboard for immunization program monitoring and decision making, Kano State, Nigeria. The Pan African Medical Journal. 2021;40(Suppl 1).

24. Thangasamy P, Gebremichael M, Kebede M, Sileshi M, Elias N, Tesfaye B. A pilot study on district health information software 2: challenges and lessons learned in a developing country: an experience from Ethiopia. Int Res J Eng Technol. 2016;3(5):1646-51.

25. Wangdi K, Sarma H, Leaburi J, McBryde E, Clements ACA. Evaluation of the malaria reporting system supported by the District Health Information System 2 in Solomon Islands. Malaria Journal. 2020;19(1):372.
