# Supplementary material for: Knowledge attributes of public health management information systems used in health emergencies: a scoping review
Source: Front Public Health. 2025 Mar 20;12:1458867. doi: 10.3389/fpubh.2024.1458867 (PMC11969037; doi:10.3389/fpubh.2024.1458867)
Supplement: SUPPLEMENTARY DATA SHEET 2 — Supplementary Tables B1 to B13. [file Data_Sheet_2.zip › SupplementaryTables_B1_B13_ArtcilesPerHMIS/SupplementaryTable_B10_Articles_ProMED.docx]

**Supplementary Table B10: List of articles included in the review on ProMED ( 23 articles)- 4 review articles, 4 research articles, 4 original papers/articles**

| **Author** | **Year of publication** | **Type of article** | **Purpose** |
| --- | --- | --- | --- |
| Al-Tawfiq,(1) | 2014 | Series | Surveillance for emerging respiratory viruses |
| Aslanov et al(2) | 2017 | Research article | internet-based surveillance system for emerging infectious diseases |
| Babalobi & Cowen (3) | 2005 | Case study | PROMED–mail: an electronic mail disease-reporting case study |
| Bijkerk et al (4) | 2017 | Report | useful information sources for Netherlands Early Warning Committee |
| Breit (5) | 2016 | Abstract | Evaluation of ProMED-mail global surveillance capability |
| Burki (6) | 2023 | World report | how ProMED-mail keeps the world alert |
| Carrion & Madoff (7) | 2017 | Review | 22 years of digital surveillance of emerging infectious diseases |
| Chang (8) | 2022 | Original Paper | Dengue Case Summary Generation |
| Chuang et al (9) | 2022 | Research article | Generating Dengue Case Summaries |
| Cowen et al (10) | 2006 | Research article | early warning system for emerging animal diseases |
| Hugh-Jones (11) | 2001 | Article | Global awareness of disease outbreaks |
| Madoff & Woodall(12) | 2005 | Review article | Lessons from the First 10 Years of ProMED-mail |
| Mitchell (13) | 1997 | Policy & people | outbreak intelligence or rash reporting? |
| Pollack et al (14) | 2013 | Highlights from ProMed | Latest outbreak news from ProMED-mail |
| Rolland et al (15) | 2020 | Journal article | Early Detection of PHEICs |
| Stewart & Denecke (16) | 2010 | Book section | Cross-Domain Pattern Analysis in Epidemic Intelligence |
| Woodall & Callisher (17) | 2001 | Conference proceedings | ProMED-mail: background and purpose |
| Woodall (18) | 2001 | Article | Global surveillance of emerging diseases |
| Yan (19) | 2017 | Review | Utility and potential |
| Yuill (20) | 2013 | Review | Latest outbreak news from ProMED-mail. |
| You et al (21) | 2021 | Journal article | text mining to track outbreak trends |
| Yu & Madoff(22) | 2024 | Journal article | an early warning system for emerging diseases |
| Zeldenrust et al (23) | 2008 | Research articles | value for the Early Warning Committee in the Netherlands |

**References**

1. Al-Tawfiq JA, Zumla A, Gautret P, Gray GC, Hui DS, Al-Rabeeah AA, et al. Emerging respiratory tract infections 1 Surveillance for emerging respiratory viruses. LANCET INFECTIOUS DISEASES. 2014;14(10):992-1000.

2. Aslanov B, Pshenichnaya N, Melnik V, Rakhmanova N. Promed-mail: internet-based surveillance system for emerging infectious diseases. Профилактическая и клиническая медицина. 2017(2):54-9.

3. Babalobi O, Cowen P. PROMED–mail: an electronic mail disease-reporting: a case study. 2005.

4. Bijkerk P, Monnier AA, Fanoy EB, Kardamanidis K, Friesema IH, Knol MJ. ECDC Round Table Report and ProMed-mail most useful international information sources for the Netherlands Early Warning Committee. Eurosurveillance. 2017;22(14):30502.

5. Breit NA, Allen T, Arnold B, Huff A, Madoff L, Pollack M. Evaluation of ProMED-mail global surveillance capability. INTERNATIONAL JOURNAL OF INFECTIOUS DISEASES. 2016;53:140-.

6. Burki T. "Unfettered flow": how ProMED-mail keeps the world alert. LANCET. 2023;401(10373):259-60.

7. Carrion M, Madoff LC. ProMED-mail: 22 years of digital surveillance of emerging infectious diseases. International Health. 2017;9(3):177-83.

8. Chang YC, Chiu YW, Chuang TW. Linguistic Pattern-Infused Dual-Channel Bidirectional Long Short-term Memory With Attention for Dengue Case Summary Generation From the Program for Monitoring Emerging Diseases-Mail Database: Algorithm Development Study. JMIR PUBLIC HEALTH AND SURVEILLANCE. 2022;8(7).

9. Chuang T, Chiu Y, Chang Y. Linguistic Pattern-infused Dual-channel BiLSTM with Attention to Generate Dengue Case Summaries from ProMED-mail database. International Journal of Infectious Diseases. 2022;116:S98-S9.

10. Cowen P, Garland T, Hugh-Jones ME, Shimshony A, Handysides S, Kaye D, et al. Evaluation of ProMED-mail as an electronic early warning system for emerging animal diseases: 1996 to 2004. Journal of the American Veterinary Medical Association. 2006;229(7):1090-9.

11. Hugh-Jones M. Global awareness of disease outbreaks: the experience of ProMED-mail. Public Health Reports. 2001;116(Suppl 2):27.

12. Madoff LC, Woodall JP. The Internet and the Global Monitoring of Emerging Diseases: Lessons from the First 10 Years of ProMED-mail. Archives of Medical Research. 2005;36(6):724-30.

13. Mitchell P. ProMED-mail: outbreak intelligence or rash reporting? LANCET. 1997;350(9091):1610-.

14. Pollack MP, Pringle C, Madoff LC, Memish ZA. Latest outbreak news from ProMED-mail: novel coronavirus–Middle East. International Journal of Infectious Diseases. 2013;17(2):e143-e4.

15. Rolland C, Lazarus C, Giese C, Monate B, Travert AS, Salomon J. Early Detection of Public Health Emergencies of International Concern through Undiagnosed Disease Reports in ProMED-Mail. Emerg Infect Dis. 2020;26(2):336-9.

16. Stewart A, Denecke K. Using ProMED-Mail and MedWorm Blogs for Cross-Domain Pattern Analysis in Epidemic Intelligence. MEDINFO 2010: IOS Press; 2010. p. 437-41.

17. Woodall J, Calisher CH. ProMED-mail: background and purpose. Emerging infectious diseases. 2001;7(3 Suppl):563.

18. Woodall JP. Global surveillance of emerging diseases: the ProMED-mail perspective. Cadernos de saude publica. 2001;17:S147-S54.

19. Yan SJ, Chughtai AA, Macintyre CR. Utility and potential of rapid epidemic intelligence from internet-based sources. INTERNATIONAL JOURNAL OF INFECTIOUS DISEASES. 2017;63:77-87.

20. Yuill TM, Woodall JP, Baekeland S. Latest outbreak news from ProMED-mail. Yellow fever outbreak-Darfur Sudan and Chad. INTERNATIONAL JOURNAL OF INFECTIOUS DISEASES. 2013;17(7):E476-E8.

21. You J, Expert P, Costelloe C. Using text mining to track outbreak trends in global surveillance of emerging diseases: ProMED-mail. Journal of the Royal Statistical Society Series A: Statistics in Society. 2021;184(4):1245-59.

22. Yu VL, Madoff LC. ProMED-mail: an early warning system for emerging diseases. Clinical infectious diseases. 2004;39(2):227-32.

23. Zeldenrust M, Rahamat-Langendoen J, Postma M, Van Vliet J. The value of ProMED-mail for the Early Warning Committee in the Netherlands: more specific approach recommended. Eurosurveillance. 2008;13(6):7-8.
