# Supplementary material for: Knowledge attributes of public health management information systems used in health emergencies: a scoping review
Source: Front Public Health. 2025 Mar 20;12:1458867. doi: 10.3389/fpubh.2024.1458867 (PMC11969037; doi:10.3389/fpubh.2024.1458867)
Supplement: SUPPLEMENTARY DATA SHEET 2 — Supplementary Tables B1 to B13. [file Data_Sheet_2.zip › SupplementaryTables_B1_B13_ArtcilesPerHMIS/SupplementaryTable_B12_Articles_WHO_GHO.docx]

**Supplementary Table B12: List of articles included in the review on WHO Global Health Observatory (34 articles)- 24 research artilces, 3 original articles, 3 Figshares, 0 reviews**

| **Author** | **Year of publication** | **Type of article** | **Purpose** |
| --- | --- | --- | --- |
| Ampofo (1) | 2020 | Research article | Modelling obesity and diabetes prevalence |
| Anderson (2) | 2016 | Research article | Tobacco Control Progress- LMIC vs HIC |
| Aggarwal (3) | 2021 | Research article | Sanitation, Obesity, and Low BMI and for Bacterial Skin Infections |
| Cui (4) | 2021 | Research article | Socioeconomic Predictors of Colon and Rectal Cancer Mortality |
| Forsea (5) | 2014 | Research article | Melanoma prognosis in Europe |
| Gomez (6) | 2021 | Original article | politics of junk food industry regulation in Latin America |
| Huang (7) | 2024 | Research article | Incidence, risk factors, and temporal trends of penile cancer: |
| Huang (8) | 2022 | Research article | Kidney Cancer & Smoking, Alcohol use, and Metabolic Syndrome |
| Huang (9) | 2021 | Research article | Burden of, Risk Factors for, and Trends in Pancreatic Cance |
| Huang (10) | 2024 | Research article | Prostate Cancer by Age, GDP, HDI, Smoking, and Alcohol |
| Jiang (11) | 2023 | Research article | Global Burden of Cataract Over the Past 30 Years |
| Juul (12) | 2015 | Figshare | consumption of ultra-processed foods and obesity in Sweden |
| Krishnamoorthy (13) | 2021 | Research article | South East Asian Region countries -interim End TB strategy targets |
| Li (14) | 2023 | Research article | homicide rates and suicide rates in 183 countries, between 2000 and 2019 |
| Li (15) | 2021 | Research article | Liver-Related Mortality From 1985 to 2015 in 36 Developed Countries |
| Liu (16) | 2017 | Research article | Global Health Workforce Labor Market Projections |
| Lu (17) | 2021 | Research article | Colorectal cancer-status, trends & risk factors in 60 countries in 2000-2019 |
| Mabaso (18) | 2018 | Research article | HDI and MDG 6 indicators SSA 2000-2014 |
| Maia (19) | 2023 | Figshare | cumulative incidence of autochthonous human cutaneous leishmaniasis |
| Maia (20) | 2023 | Figshare | cumulative incidence of autochthonous human visceral leishmaniasis |
| Nazir (21) | 2019 | Research article | Tobacco Use in Adolescents |
| Onagbiye (22) | 2023 | Research article | Sedentariness and overweight in relation to mortality in sub-Saharan Africa. |
| Patel (23) | 2015 | Conference abstract | Years of Life Lost Due to Heart Failure in the United States (US) |
| Rodi (24) | 2022 | Figshare | DAH, number of deaths, mortality rates, and health expenditures |
| Shah (25) | 2017 | Conference abstract | Cold weather, sunlight hours and alcohol-related cirrhosis |
| Stefko (26) | 2017 | Original article | Management and funding of the health care system |
| Swarnamali (27) | 2022 | Research article | per capita fat supply and prevalence of overweight and obesity |
| Tidman (28) | 2023 | Research article | 4 neglected foodborne trematodes targeted for control by WHO |
| Vardell (29) | 2020 | Online updates | Global health observatory data repository |
| van Rensburg (30) | 2022 | Original article | current psychiatrist workforce in South Africa |
| Wang (31) | 2024 | Research article | diabetes attributable to second-hand smoke and smoke-free policies |
| Wu (32) | 2023 | Research article | Trachoma: The remaining gap in care & handwashing facilities |
| Yesilaydin (33) | 2022 | Research article | life expectancy and mental health systems in European countries |
| Zaveri (34) | 2013 | Research article | Publishing and interlinking the Global Health Observatory dataset |

**References**

1. Ampofo AG, Boateng EB. Beyond 2020: Modelling obesity and diabetes prevalence. DIABETES RESEARCH AND CLINICAL PRACTICE. 2020;167.

2. Anderson CL, Becher H, Winkler V. Tobacco Control Progress in Low and Middle Income Countries in Comparison to High Income Countries. INTERNATIONAL JOURNAL OF ENVIRONMENTAL RESEARCH AND PUBLIC HEALTH. 2016;13(10).

3. Aggarwal P, Muddasani S, Fleischer AB, Jr. Sanitation, Obesity, and Low Body Mass Index as Risk Factors for Bacterial Skin Infections. JOURNAL OF CUTANEOUS MEDICINE AND SURGERY. 2021;25(3):293-7.

4. Cui CL, Dornisch AM, Umlauf AE, Cuomo RE, Murphy JD, Lopez NE. International Socioeconomic Predictors of Colon and Rectal Cancer Mortality: Is Colorectal Cancer a First World Problem? JCO GLOBAL ONCOLOGY. 2021;7:1659-67.

5. Forsea AM, del Marmol V, Stratigos A, Geller AC. Melanoma prognosis in Europe: far from equal. BRITISH JOURNAL OF DERMATOLOGY. 2014;171(1):179-82.

6. Gomez EJ. Getting to the root of the problem: the international and domestic politics of junk food industry regulation in Latin America. HEALTH POLICY AND PLANNING. 2021;36(10):1521-33.

7. Huang J, Chan SC, Pang WS, Liu X, Zhang L, Lucero-Prisno Iii DE, et al. Incidence, risk factors, and temporal trends of penile cancer: a global population-based study. BJU INTERNATIONAL. 2024;133(3):314-23.

8. Huang J, Leung DK-W, Chan EO-T, Lok V, Leung S, Wong I, et al. A Global Trend Analysis of Kidney Cancer Incidence and Mortality and Their Associations with Smoking, Alcohol Consumption, and Metabolic Syndrome. EUROPEAN UROLOGY FOCUS. 2022;8(1):200-9.

9. Huang J, Lok V, Ngai CH, Zhang L, Yuan J, Lao XQ, et al. Worldwide Burden of, Risk Factors for, and Trends in Pancreatic Cancer. GASTROENTEROLOGY. 2021;160(3):744-54.

10. Huang J, Chan EO-T, Liu X, Lok V, Ngai CH, Zhang L, et al. Global Trends of Prostate Cancer by Age, and Their Associations With Gross Domestic Product (GDP), Human Development Index (HDI), Smoking, and Alcohol Drinking. CLINICAL GENITOURINARY CANCER. 2023;21(4):E261-+.

11. Jiang B, Wu T, Liu W, Liu G, Lu P. Changing Trends in the Global Burden of Cataract Over the Past 30 Years: Retrospective Data Analysis of the Global Burden of Disease Study 2019. JMIR PUBLIC HEALTH AND SURVEILLANCE. 2023;9.

12. Juul F, Hemmingsson E. Trends in consumption of ultra-processed foods and obesity in Sweden between 1960 and 2010. PUBLIC HEALTH NUTRITION. 2015;18(17):3096-107.

13. Krishnamoorthy Y, Nagarajan R, Rajaa S, Majella MG, Murali S, Jayaseelan V. Progress of South East Asian Region countries towards achieving interim End TB strategy targets for TB incidence and mortality: a modelling study. PUBLIC HEALTH. 2021;198:9-16.

14. Li Z, Cai Z, Yip PSF. One stream, two channels? A parallel-process latent class growth model of homicide rates and suicide rates in 183 countries, between 2000 and 2019. SSM-POPULATION HEALTH. 2023;22.

15. Li W, Kemos P, Salciccioli JD, Marshall DC, Shalhoub J, Alazawi W. Socioeconomic Factors Associated With Liver-Related Mortality From 1985 to 2015 in 36 Developed Countries. CLINICAL GASTROENTEROLOGY AND HEPATOLOGY. 2021;19(8):1698-+.

16. Liu JX, Goryakin Y, Maeda A, Bruckner T, Scheffler R. Global Health Workforce Labor Market Projections for 2030. HUMAN RESOURCES FOR HEALTH. 2017;15.

17. Lu B, Li N, Luo C-Y, Cai J, Lu M, Zhang Y-H, et al. Colorectal cancer incidence and mortality: the current status, temporal trends and their attributable risk factors in 60 countries in 2000-2019. CHINESE MEDICAL JOURNAL. 2021;134(16):1941-51.

18. Mabaso MLH, Zama TP, Mlangeni L, Mbiza S, Mkhize-Kwitshana ZL. Association between the Human Development Index and Millennium Development Goals 6 Indicators in Sub-Saharan Africa from 2000 to 2014: Implications for the New Sustainable Development Goals. JOURNAL OF EPIDEMIOLOGY AND GLOBAL HEALTH. 2018;8(1-2):77-81.

19. Maia C, Conceicao C, Pereira A, Rocha R, Ortuno M, Munoz C, et al. Annual cumulative incidence per 100,000 population of autochthonous human cutaneous leishmaniasis in European countries between 2005 and 2020. Figshare2023.

20. Maia C, Conceicao C, Pereira A, Rocha R, Ortuno M, Munoz C, et al. Annual cumulative incidence per 100,000 population of human autochthonous visceral leishmaniasis in European countries between 2005 and 2020. Figshare2023.

21. Nazir MA, Al-Ansari A, Abbasi N, Almas K. Global Prevalence of Tobacco Use in Adolescents and Its Adverse Oral Health Consequences. Open access Macedonian journal of medical sciences. 2019;7(21):3659-66.

22. Onagbiye S, Ricci H, Bester P, Ricci C. Sedentariness and overweight in relation to mortality in sub-Saharan Africa. A mediation analysis based on the World Health Organization-Global Health Observatory data repository. JOURNAL OF PUBLIC HEALTH IN AFRICA. 2023;14(4).

23. Patel H, Kielhorn A, Yurgin N, Hernandez AF. Years of Life Lost Due to Heart Failure in the United States (US). CIRCULATION. 2015;132.

24. Rodi P, Obermeyer W, Pablos-Mendez A, Gori A, Raviglione MC. Overview of DAH, number of deaths, mortality rates, and health expenditures. Figshare2022.

25. Shah ND, Cruz-Lemini M, Stein E, Abraldes J, Altamirano J, Bataller R. COLDER WEATHER AND FEWER SUNLIGHT HOURS INCREASE THE WEIGHT OF ALCOHOL AS A CAUSE OF CIRRHOSIS WORLDWIDE. GASTROENTEROLOGY. 2017;152(5):S942-S3.

26. Stefko R, Jencova S, Litavcova E, Vasanicova P. MANAGEMENT AND FUNDING OF THE HEALTHCARE SYSTEM. POLISH JOURNAL OF MANAGEMENT STUDIES. 2017;16(2):266-77.

27. Swarnamali H, Jayawardena R, Chourdakis M, Ranasinghe P. Is the proportion of per capita fat supply associated with the prevalence of overweight and obesity? an ecological analysis. BMC NUTRITION. 2022;8(1).

28. Tidman R, Kanankege KST, Bangert M, Abela-Ridder B. Global prevalence of 4 neglected foodborne trematodes targeted for control by WHO: A scoping review to highlight the gaps. PLOS NEGLECTED TROPICAL DISEASES. 2023;17(3).

29. Vardell E. Global health observatory data repository. Medical reference services quarterly. 2020;39(1):67-74.

30. van Rensburg BJ, Kotze C, Moxley K, Subramaney U, Zingela Z, Seedat S. Profile of the current psychiatrist workforce in South Africa: establishing a baseline for human resource planning and strategy. HEALTH POLICY AND PLANNING. 2022;37(4):492-504.

31. Wang M, Maimaitiming M, Zhao Y, Jin Y, Zheng ZJ. Global trends in deaths and disability-adjusted life years of diabetes attributable to second-hand smoke and the association with smoke-free policies. PUBLIC HEALTH. 2024;228:18-27.

32. Wu TJ, Reynolds MM. Trachoma, the world's leading infectious cause of blindness: The remaining gap in care and access to basic handwashing facilities. EUROPEAN JOURNAL OF OPHTHALMOLOGY. 2023;33(4):1576-82.

33. Yesilaydin G, Uslu E. The relationship between life expectancy and mental health systems in European countries. JOURNAL OF PSYCHIATRIC NURSING. 2022;13(3):199-204.

34. Zaveri A, Lehmann J, Auer S, Hassan MM, Sherif MA, Martin M. Publishing and interlinking the global health observatory dataset. Semantic web. 2013;4(3):315-22.
