# Supplementary material for: Knowledge attributes of public health management information systems used in health emergencies: a scoping review
Source: Front Public Health. 2025 Mar 20;12:1458867. doi: 10.3389/fpubh.2024.1458867 (PMC11969037; doi:10.3389/fpubh.2024.1458867)
Supplement: SUPPLEMENTARY DATA SHEET 2 — Supplementary Tables B1 to B13. [file Data_Sheet_2.zip › SupplementaryTables_B1_B13_ArtcilesPerHMIS/SupplementaryTable_B13_Articles_COVID19.docx]

**Supplementary Table B13: List of articles included in the review on WHO COVID-19 dashboard- 1 research article, 1 review article**

| **Author** | **Year of publication** | **Type of article** | **Purpose** |
| --- | --- | --- | --- |
| Ahmed et al (1) | 2020 | Review article | Data summarization & visualization tool for the WHO AFRO region |
| Maya et al (2) | 2022 | Methodology article | A description of the WHO COVID-19 surveillance database |
| Iwansyah et al(3) | 2020 | Preprint | Monitoring COVID-19/SARS-CoV-2 Pandemic using GIS |
| Ivanković et al (4) | 2021 | Research article | Features of 158 actionable public COVID-19 dashboards |
| Sulaiman et al (5) | 2020 | Conference proceedings | Geospatial dashboards to map & track the novel coronavirus pandemic |
| WHO (6) | 2023 | Website | The COVID-19 dashboard |

**References**

1. Ahmed K, Bukhari MA, Mlanda T, Kimenyi JP, Wallace P, Lukoya CO, et al. Novel approach to support rapid data collection, management, and visualization during the COVID-19 outbreak response in the world health organization African region: development of a data summarization and visualization tool. JMIR Public Health and Surveillance. 2020;6(4):e20355.

2. Allan M, Lièvre M, Laurenson-Schafer H, de Barros S, Jinnai Y, Andrews S, et al. The World Health Organization COVID-19 surveillance database. International journal for equity in health. 2022;21(Suppl 3):167.

3. Irwansyah E, Budiharto W, Widhyatmoko D, Istamar A, Panghurian FP. Monitoring Coronavirus COVID-19/SARS-CoV-2 Pandemic using GIS Dashboard: International and Indonesia Context. Preprints 2020.

4. Ivanković D, Barbazza E, Bos V, Brito Fernandes Ó, Jamieson Gilmore K, Jansen T, et al. Features constituting actionable COVID-19 dashboards: descriptive assessment and expert appraisal of 158 public web-based COVID-19 dashboards. Journal of medical Internet research. 2021;23(2):e25682.

5. Sulaiman N, Abid SK, Chan SW, Nazir U, Mahmud NPN, Latib S, et al., editors. Geospatial dashboards for mapping and tracking of novel coronavirus pandemic. Proc Int Conf Ind Eng Oper Manag; 2020.

6. World Health Organization. WHO COVID-19 dashboard2023, . Available from: <https://data.who.int/dashboards/covid19/about?n=c>.
