# Supplementary material for: Knowledge attributes of public health management information systems used in health emergencies: a scoping review
Source: Front Public Health. 2025 Mar 20;12:1458867. doi: 10.3389/fpubh.2024.1458867 (PMC11969037; doi:10.3389/fpubh.2024.1458867)
Supplement: SUPPLEMENTARY DATA SHEET 2 — Supplementary Tables B1 to B13. [file Data_Sheet_2.zip › SupplementaryTables_B1_B13_ArtcilesPerHMIS/SupplementaryTable_B2_Articles_EOC.docx]

**Supplementary Table B2: List of articles included in the review on EOCs (19 articles)- 3 research articles, 3 review article s**

| **1^st^ Author (s)** | **Year of publication** | **Type of article** | **Purpose** |
| --- | --- | --- | --- |
| Allen & Spencer (1) | 2023 | Scoping review | Barriers and Enablers to using EOCs |
| Becerra-Fernández et al (2) | 2008 | Conference proceedings | Design and development of a virtual EOCs |
| Bousso, A. (3) | 2019 | Original article | EOC implementation challenges in Africa |
| Chan et al (4) | 2016 | Book chapter | Envisioning the EOC of the future |
| Callan, T (5) | 2020 | Topical collection | EOCs: models and core principles |
| Challa (6) | 2023 | Brief report | Lesson From Louse-Borne Relapsing Fever Outbreak in Ethiopia |
| Chipman & Wuerfel (7) | 2008 | Conference proceedings | Network based information sharing between EOCs |
| Clark et al (8) | 2011 | Feature | EOCs or command post: When, where, and how |
| Davis (9) | 2002 | Magazine | Virtual EOCs |
| Harris (10) | 2009 | Thesis | use of networks to connect local emergency operations centers |
| Hood (11) | 2022 | Thesis | EOC Organization and Operations in a FEMA Region |
| Kayiwa (12) | 2022 | Original article | EOC establishment in an Outbreak-Prone Country- Uganda |
| Ma et al (13) | 2017 | Review article | EOC use for pandemic response: opportunities and challenges |
| Ryan (14) | 2013 | Research article | Planning in the EOC |
| Su et al (15) | 2017 | Special Feature | EOC use in Taiwan |
| Shojaei et al (16) | 2023 | Research article | Need for EOC by emergency responders in Iran |
| WHO (17) | 2014 | Meeting notes | EOC-NET consultation meeting |
| WHO (18) | 2014 | Systematic review | A global analysis of 291 studies on EOCs |
| Xu & Li (19) | 2015 | Research article | Analysis of good practice of EOCs |

**References**

1. Allen T, Spencer R. Barriers and Enablers to Using an Emergency Operations Center in Public Health Emergency Management: A Scoping Review. Disaster Medicine and Public Health Preparedness. 2023;17:e407.

2. Becerra-Fernández I, Madey G, Prietula M, Rodríguez D, Valerdi R, Wright T, editors. Design and development of a virtual emergency operations center for disaster management research, training, and discovery. Proceedings of the 41st Annual Hawaii International Conference on System Sciences (HICSS 2008); 2008: IEEE.

3. Bousso A. Health emergency operation centers implementation challenges in Africa. PAN AFRICAN MEDICAL JOURNAL. 2019;33.

4. Chan E, Anslow C, Seyed T, Maurer F. Envisioning the emergency operations centre of the future. Collaboration Meets Interactive Spaces. 2016:349-72.

5. Callan T. Emergency operations centres: models and core principles. REVUE SCIENTIFIQUE ET TECHNIQUE-OFFICE INTERNATIONAL DES EPIZOOTIES. 2020;39(2):399-405.

6. Chala TK, Abera EG, Tukeni KN, Didu GH, Abbagidi FA, Yesuf EA, et al. The Need to Establish and Sustain Public Health Emergency Operation Centers for Managing Infectious Disease Outbreaks: Lesson From Response to Louse-Borne Relapsing Fever Outbreak in Jimma, Ethiopia. DISASTER MEDICINE AND PUBLIC HEALTH PREPAREDNESS. 2023;17.

7. Chipman R, Wuerfel R, editors. Network based information sharing between emergency operations center. 2008 IEEE Conference on Technologies for Homeland Security; 2008: IEEE.

8. Clark A, Hooper B, Gibbs J. Emergency operation centers or hardened command post: When, where, and how? Journal of Chemical Health & Safety. 2011;18(3):10-4.

9. Davis SC. Virtual emergency operations centers. Risk Management. 2002;49(7):46.

10. Harris EA. The use of networks to connect local emergency operations centers: Oklahoma State University; 2009.

11. Hood SN. Understanding Emergency Operations Center Organization and Operations in a FEMA Region: Capella University; 2022.

12. Kayiwa J, Homsy J, Nelson LJ, Ocom F, Kasule JN, Wetaka MM, et al. Establishing a Public Health Emergency Operations Center in an Outbreak-Prone Country: Lessons Learned in Uganda, January 2014 to December 2021. HEALTH SECURITY. 2022;20(5):394-407.

13. Ma J, Huang Y, Zheng Z-J. Leveraging the Public Health Emergency Operation Center (PHEOC) for pandemic response: opportunities and challenges. Global health journal (Amsterdam, Netherlands). 2020;4(4):118-20.

14. Ryan M. Planning in the emergency operations center. Technological forecasting and social change. 2013;80(9):1725-31.

15. Su Y-F, Wu C-H, Lee T-F. PUBLIC HEALTH EMERGENCY RESPONSE IN TAIWAN. HEALTH SECURITY. 2017;15(2):137-43.

16. Shojaei F, Qaraeian P, Firoozbakht A, Chhabra D, Jahangiri K. The necessity for an integrated Emergency Operations Center (EOC) among first responders: Lesson learned from two Iranian railway accidents. Heliyon. 2023;9(5).

17. World Health Organization. Public health emergency operations centre network (EOC-NET): consultation meeting, 19-20 November 2012, Geneva, Switzerland2013. Available from: <https://apps.who.int/iris/bitstream/handle/10665/85378/WHO_HSE_?sequence=1>.

18. World Health Organization. A systematic review of public health emergency operations centres (EOC): December 20132014. Available from: <https://www.who.int/publications/i/item/WHO-HSE-GCR-2014.1>.

19. Xu M, Li S-X. Analysis of good practice of public health Emergency Operations Centers. Asian Pacific journal of tropical medicine. 2015;8(8):677-82.
