# Supplementary material for: Knowledge attributes of public health management information systems used in health emergencies: a scoping review
Source: Front Public Health. 2025 Mar 20;12:1458867. doi: 10.3389/fpubh.2024.1458867 (PMC11969037; doi:10.3389/fpubh.2024.1458867)
Supplement: SUPPLEMENTARY DATA SHEET 2 — Supplementary Tables B1 to B13. [file Data_Sheet_2.zip › SupplementaryTables_B1_B13_ArtcilesPerHMIS/SupplementaryTable_B4_Articles_GLEWS.docx]

**Supplementary Table B4: List of articles included in the review on GLEWS-14 articles. – 3 review articles , 2 research articles**

| **Author** | **Year of publication** | **Type of article** | **Purpose** |
| --- | --- | --- | --- |
| Al-Hemoud et al (1) | 2021 | Research article | Early Detection System of Infectious Diseases & Animal Health |
| Arnoldi et al (2) | 2004 | Conference proceedings | USAHA/AAVLD Committee on International Standards |
| Caceres (3) | 2016 | Abstract | improving OIE's monitoring and early warning systems |
| OIE, FAO, WHO (4) | 2006 | Institutional document | Overview of the GLEWS initiative |
| Jebara (5) | 2004 | Review article | Surveillance, detection and response at international level |
| Kisman et al (6) | 2010 | Topical collection | International Standards and Strategies for the Surveillance |
| Kshirsagar et al (7) | 2013 | Review article | Disease alerts and forecasting of zoonotic diseases: an overview |
| Lin et al (8) | 2023 | Research article | Analysing WAHIS Animal Health Immediate Notification |
| Marvin et al (9) | 2009 | Review article | Early identification systems for emerging foodborne hazards |
| Savelli (10) | 2013 | Article | response to animal diseases transmissable to humans via food |
| Thakur (11) | 2022 | Book section | Early Warning Sys, Disease Management, Biosecurity in Disasters |
| Tekola(12) | 2017 | Topical collection | International health threats and global early warning and response |
| Tounkara et al (13) | 2019 | Book section | Transboundary Animal Diseases (TADs) Surveillance and Control |
| Vandersmissen & Welburn | 2014 | Article | Current initiatives in One Health |
| World Organization for Animal health (14) | 2023 | Website | WAHIS: World Animal Health Information System |
| World Organization for Animal health (15) | nd | Website | About GLEWS and GLEWS+. |

**References**

1. Al-Hemoud A, AlSaraf M, Malak M, Al-Shatti M, Al-Jarba M, Othman A, et al. Analytical and Early Detection System of Infectious Diseases and Animal Health Status in Kuwait. Frontiers in Veterinary Science. 2021;8:676661.

2. Arnoldi JM, David MJ, Fernandez PJ, Fischer JR, Frost B, Lautner EA, et al., editors. Report of the USAHA/AAVLD Committee on International Standards. PROCEEDINGS OF THE ANNUAL MEETING-UNITED STATES ANIMAL HEALTH ASSOCIATION; 2004: United States Health Association; 1998.

3. Caceres P. Tracking activity to improve the sensitivity of the OIE's monitoring and early warning systems for human and animal diseases. International Journal of Infectious Diseases. 2016;53:11.

4. FAO, OIE, WHO. Global Early Warning and

Response System for Major Animal Diseases, including Zoonoses (GLEWS)2006. Available from: <https://www.woah.org/app/uploads/2021/03/glews-tripartite-finalversion010206.pdf>.

5. Jebara KB. Surveillance, detection and response: managing emerging diseases at national and international levels. Rev Sci Tech. 2004;23(2):709-15.

6. Kisman M, Donev D, Kisman A. International Standards and Strategies for the Surveillance. Prevention and Control of Brucellosis Maced J Med Sci. 2010;3(3):273-7.

7. Kshirsagar D, Savalia C, Kalyani I, Kumar R, Nayak D. Disease alerts and forecasting of zoonotic diseases: an overview. Veterinary World. 2013;6(11):889.

8. Lin S-Y, Beltran-Alcrudo D, Awada L, Hamilton-West C, Lavarello Schettini A, Cáceres P, et al. Analysing WAHIS Animal Health Immediate Notifications to Understand Global Reporting Trends and Measure Early Warning Capacities (2005–2021). Transboundary and Emerging Diseases. 2023;2023:1-10.

9. Marvin H, Kleter G, Prandini A, Dekkers S, Bolton D. Early identification systems for emerging foodborne hazards. Food and Chemical Toxicology. 2009;47(5):915-26.

10. Savelli CJ, Abela-Ridder B, Miyagishima K. Planning for rapid response to outbreaks of animal diseases transmissable to humans via food. REVUE SCIENTIFIQUE ET TECHNIQUE-OFFICE INTERNATIONAL DES EPIZOOTIES. 2013;32(2):469-77.

11. Thakur SD. Early Warning Systems, Disease Management, and Biosecurity in Disasters. Management of Animals in Disasters: Springer; 2022. p. 25-37.

12. Tekola B, Myers L, Lubroth J, Plee L, Calistri P, Pinto J. International health threats and global early warning and response mechanisms. REVUE SCIENTIFIQUE ET TECHNIQUE-OFFICE INTERNATIONAL DES EPIZOOTIES. 2017;36(2):657-70.

13. Tounkara K, Couacy-Hymann E, Diall O. Transboundary Animal Diseases (TADs) Surveillance and Control (Including National Veterinary Services, Regional Approach, Regional and International Organisations, GF-TAD). In: Kardjadj M, Diallo A, Lancelot R, editors. Transboundary Animal Diseases in Sahelian Africa and Connected Regions. Cham: Springer International Publishing; 2019. p. 53-68.

14. World Organisation for Animal Health. WAHIS: World Animal Health Information System2023. Available from: <https://wahis.woah.org/#/home>.

15. World Organisation for Animal Health. About GLEWS and GLEWS+.nd. Available from: <http://www.glews.net/?page_id=1059>.
