# Supplementary material for: Knowledge attributes of public health management information systems used in health emergencies: a scoping review
Source: Front Public Health. 2025 Mar 20;12:1458867. doi: 10.3389/fpubh.2024.1458867 (PMC11969037; doi:10.3389/fpubh.2024.1458867)
Supplement: SUPPLEMENTARY DATA SHEET 2 — Supplementary Tables B1 to B13. [file Data_Sheet_2.zip › SupplementaryTables_B1_B13_ArtcilesPerHMIS/SupplementaryTable_B7_Articles_HDX.docx]

**Supplementary Table B7: List of articles included in the review on HDX- 22 articles- 14 maps, no research articles, no review articles**

| **Author** | **Year of publication** | **Type of article** | **Purpose** |
| --- | --- | --- | --- |
| Abuoda et al (1) | 2021 | Conference proceedings | Automatic Tag Recommendation |
| Balsari et al (2) | 2022 | White paper | Human Mobility Data in Public Health Emergencies |
| Berens et al (3) | 2016 | Institutional publication | The humanitarian data ecosystem |
| Charniga et al (4) | 2021 | Map | Maps of ZVD incidence in Colombia |
| Charniga et al (5) | 2021 | Map | Maps of ZIKV-associated neurological complications by department |
| Gao et al (6) | 2022 | Map | COVID-19 infected healthcare workers |
| Gibbs et tal (7) | 2022 | Map | Estimates of Rt in individual districts. |
| Hierink et al (8) | 2022 | Map | 17 administrative regions of the Philippines accessibility analysis |
| Kelly(9) | 2020 | Monthly series | Humanitarian evidence summary No. 7 |
| MacPherson et al (10) | 2023 | Map | Location of households sampled for interview in Malawi |
| Metwally et al (11) | 2023 | Map | Map of the 27 Egyptians governorates in 4 geographical regions |
| Moulds et al (12) | 2022a | Map | Local Indicators of Spatial Association clusters for Ghana |
| Moulds et al (12) | 2022b | Map | households drinking sachet water in Ghanas 170 districts |
| Moulds et al (13) | 2022c | Map | source of drinking water in Ghanas 170 districts |
| Nyabinwa et al (14) | 2020 | Map | Farmers’ perception on endometritis prevention & control measures |
| Nyakarahuka et al (15) | 2023 | Map | seroprevalence of Crimean-Congo hemorrhagic fever virus IgG |
| Paulus et al (16) | 2016 | Conference paper | Turning data into action |
| Ross(17) | 2018 | Report | Research Tools In The Phase Zero Digital Toolbox. |
| Ross et al (18) | 2022 | Map | Map of Bangladesh survey sites |
| Telford (19) | 2020 | Case study | HDX: Critical Decisions, Key Results and The Road Ahead |
| Weaver et al (20) | 2019 | Book section | Development assistance and humanitarian action |
| Yan et al (21) | 2022 | Map | Map of census blocks sampled in Haiti |

**References**

1. Abuoda G, Hendrix C, Campo S, editors. Automatic Tag Recommendation for the UN Humanitarian Data Exchange. BIRDS+ WEPIR@ CHIIR; 2021.

2. Balsari S, Buckee C, Chan J, Schroeder A. The Use of Human Mobility Data in Public Health Emergencies. 2022.

3. Berens J, Raymond N, Shimshon G, Verhulst S, Bernholz L. The humanitarian data ecosystem: The case for collective responsibility. Stanford Center on Philanthropy and Civil Society. <https://pacscenter> …; 2016.

4. Charniga K, Cucunuba ZM, Walteros DM, Mercado M, Prieto F, Ospina M, et al. Maps of ZVD incidence in Colombia. Figshare2021.

5. Charniga K, Cucunuba ZM, Walteros DM, Mercado M, Prieto F, Ospina M, et al. Maps of ZIKV-associated neurological complications by department in Colombia. Figshare2021.

6. Gao W, Sanna M, Tsai MK, Wen CP. Percentage of COVID-19 infected healthcare workers in severe conditions in different geographic locations and over three ten-day periods, based on symptom onset dates. Figshare2020.

7. Gibbs H, Liu Y, Abbott S, Baffoe-Nyarko I, Laryea DO, Akyereko E, et al. Estimates of Rt in individual districts. Figshare2022.

8. Hierink F, Margutti J, Van Den Homberg M, Ray N. Overview of all results. Figshare2022.

9. Kelly L. Humanitarian evidence summary No. 7. 2020.

10. MacPherson EE, Mankhomwa J, Dixon J, Pongolani R, Phiri M, Feasey N, et al. Location of households sampled for interview. Figshare2023.

11. Metwally AM, Nassar MS, El-Din EM, Abdallah AM, Khadr Z, Abouelnaga MW, et al. Map of the 27 Egyptians governorates distributed within the four geographic regions (adapted using data from the Humanitarian Data Exchange under the CC BY-IGO license [18]. Figshare2023.

12. Moulds S, Chan ACH, Tetteh JD, Bixby H, Owusu G, Agyei-Mensah S, et al. Percentage of households drinking sachet water in Ghanas 170 districts. Figshare2022.

13. Moulds S, Chan ACH, Tetteh JD, Bixby H, Owusu G, Agyei-Mensah S, et al. Most common source of drinking water in Ghanas 170 districts. Figshare2022.

14. Nyabinwa P, Kashongwe OB, Hirwa CD, Bebe BO. Additional file 1 of Perception of farmers about endometritis prevention and control measures for zero-grazed dairy cows on smallholder farms in Rwanda. Figshare2020.

15. Nyakarahuka L, Kyondo J, Telford C, Whitesell A, Tumusiime A, Mulei S, et al. Sampled districts and their corresponding seroprevalence of Crimean-Congo hemorrhagic fever virus IgG antibodies in cattle, sheep and goats (Open-source shapefiles for Uganda district boundaries were downloaded from the Humanitarian Data Exchange (Humanitarian Data Exchange, 2020) and water bodies files from the World Bank website (The World Bank, 2022)). Figshare2023.

16. Paulus D, Meesters K, Van de Walle BA, editors. Turning data into action: supporting humanitarian field workers with open data. Iscram; 2018.

17. Ross DW. Research Tools In The Phase Zero Digital Toolbox2018. Available from: <https://www.jstor.org/stable/pdf/resrep24319.4.pdf>.

18. Ross YB, Hoque M, Blanton JD, Kennedy ED, Rana MS, Tahmina S, et al. Map of Bangladesh survey sites and the distribution of household surveys by survey site. Figshare2022.

19. Telford S. Case Study-The Humanitarian Data Exchange: Critical Decisions, Key Results and The Road Ahead2020. Available from: <https://centre.humdata.org/wp-content/uploads/2020/09/hdxcasestudy.pdf>.

20. Weaver C, Powell J, Leson H. Development assistance and humanitarian action. 2019. In: The State of Open Data [Internet]. [77].

21. Yan LD, McNairy ML, Devieux JG, Pierre JL, Dade E, Sufra R, et al. Map of census blocks sampled in Haiti CVD cohort. Figshare2022.
