# Supplementary material for: Knowledge attributes of public health management information systems used in health emergencies: a scoping review
Source: Front Public Health. 2025 Mar 20;12:1458867. doi: 10.3389/fpubh.2024.1458867 (PMC11969037; doi:10.3389/fpubh.2024.1458867)
Supplement: SUPPLEMENTARY DATA SHEET 2 — Supplementary Tables B1 to B13. [file Data_Sheet_2.zip › SupplementaryTables_B1_B13_ArtcilesPerHMIS/SupplementaryTable_B9_Articles_OpenWHO.docx]

**Supplementary Table B9: List of articles included in the review on OpenWHO (21 articles)- 7 conference proceedings, 7 research articles**

| **Author** | **Year of publication** | **Type of article** | **Purpose** |
| --- | --- | --- | --- |
| Attias et al (1) | 2022 | Conference proceedings | Open-Access Learning as a Pathway to Equity |
| Bonkoungou, B., et al(2) | 2023 | Research article | Online capacity building for the health workforce |
| George et al(3) | 2020 | Conference proceedings | Uptake of OpenWHO's Online Learning Resources for COVID-19 |
| George et al (4) | 2022 | Commentary | Ensuring equity in access to online courses |
| Goldin et al (5) | 2021 | Research article | Learning From a Online COVID-19 Vaccination Training Experience |
| Rohloff et al (6) | 2018 | Conference proceedings | Online Knowledge Transfer into Health Emergency Response |
| Samo et al (7) | 2020 | Article | Learning Content in Italian for COVID-19 Frontline Responders |
| Utunen, Christen et al(8) | 2018 | Conference proceedings | Knowledge transfer for Ebola outbreak–production |
| Utunen, Attias, et al., (9) | 2020 | Conference proceedings | Access to OpenWHO's Online Learning Resources for COVID-19 |
| Utunen, George et al(10) | 2020 | Research article | Responding to Global Learning Needs during a Pandemi |
| Utunen, Ndiaye et al(11) | 2020 | Research article | Global reach of an online COVID-19 course in multiple languages |
| Utunen, Ndiaye, et al., (12) | 2021 | Research article | Users Trends Before and During the COVID-19 Pandemic |
| Utunen, H.(13) | 2021 | Conference proceedings | Transferring real-time knowledge free of charge |
| Utunen, Van Kerkhove et al(14) | 2021 | Viewpoint | One year of pandemic learning response |
| Utunen, George et al(15) | 2021 | Book section | Delivering WHO’s life-saving information during a pandemic |
| Utunen, Attias et al(16) | 2022 | Article | Learning multiplier effect of OpenWHO |
| Utunen, Mattar et al (17) | 2022 | Research article | Superusers of self-paced online learning on OpenWHO |
| Utunen, Ndiaye et al(18) | 2022 | conference proceedings | Multilingual Approach to COVID-19 Online Learning Response |
| Utunen ,Appuhamy et al (19) | 2023 | Research article | Observations from three years of online pandemic learning response o |
| Utunen, Staubitz et al (20) | 2023 | Book section | Scale Up Multilingualism in Health Emergency Learning |
| Utunen, Tokar et al (21) | 2023 | comment | Online learning for WHO priority diseases with pandemic potential |

**References**

1. Attias M, Utunen H, Ndiaye N, Mattar L, editors. Open-Access Learning as a Pathway to Equity During Health Emergencies. The Learning Ideas Conference; 2022: Springer.

2. Bonkoungou B, Utunen H, Talisuna AO, O'Connell G, Koua E, Chamla DD, et al. Online capacity building for the health workforce: the case of the Integrated Disease Surveillance and Response for the African region. JOURNAL OF PUBLIC HEALTH IN AFRICA. 2023;14(12).

3. George R, Utunen H, Attias M, Sy A, Ndiaye N, Piroux C, et al. An Analysis of the Growth in Uptake of OpenWHO's Online Learning Resources for COVID-19. IMPORTANCE OF HEALTH INFORMATICS IN PUBLIC HEALTH DURING A PANDEMIC; 20202020. p. 284-7.

4. George R, Utunen H, Ndiaye N, Tokar A, Mattar L, Piroux C, et al. Ensuring equity in access to online courses: Perspectives from the WHO health emergency learning response. World Medical & Health Policy. 2022;14(2):413-27.

5. Goldin S, Kong SYJ, Tokar A, Utunen H, Ndiaye N, Bahl J, et al. Learning From a Massive Open Online COVID-19 Vaccination Training Experience: Survey Study. JMIR PUBLIC HEALTH AND SURVEILLANCE. 2021;7(12).

6. Rohloff T, Utunen H, Renz J, Zhao Y, Gamhewage G, Meinel C, editors. OpenWHO: Integrating Online Knowledge Transfer into Health Emergency Response. EC-TEL (Practitioner Proceedings); 2018.

7. Samo G, Zhao UY, Gamhewage G. Syntactic Complexity of Learning Content in Italian for COVID-19 Frontline Responders: A Study on WHO’s Emergency Learning Platform. Verbum. 2020;11.

8. Utunen H, Christen P, Gamhewage GM, Zhao U, Attias M, editors. Knowledge transfer for Ebola outbreak–production and use of OpenWHO. org online learning resources. 2018 14th International Conference on Wireless and Mobile Computing, Networking and Communications (WiMob); 2018: IEEE.

9. Utunen H, Attias M, George R, Ndiaye N, Piroux C, Farzi MR, et al. Global Access to OpenWHO's Online Learning Resources for COVID-19. IMPORTANCE OF HEALTH INFORMATICS IN PUBLIC HEALTH DURING A PANDEMIC; 20202020. p. 304-5.

10. Utunen H, George R, Ndiaye N, Attias M, Piroux C, Gamhewage G. Responding to global learning needs during a pandemic: an analysis of the trends in platform use and incidence of COVID-19. Education Sciences. 2020;10(11):345.

11. Utunen H, Ndiaye N, Piroux C, George R, Attias M, Gamhewage G. Global reach of an online COVID-19 course in multiple languages on OpenWHO in the first quarter of 2020: analysis of platform use data. Journal of Medical Internet Research. 2020;22(4):e19076.

12. Utunen H, Ndiaye N, Mattar L, Christen P, Stucke O, Gamhewage G. Changes in Users Trends Before and During the COVID-19 Pandemic on WHO's Online Learning Platform. Studies in health technology and informatics. 2021;287:163-4.

13. Utunen H. Transferring real-time knowledge free of charge through WHO’s online learning platform OpenWHO. org. QScience Proceedings. 2021;2022(1):5.

14. Utunen H, Van Kerkhove MD, Tokar A, O'Connell G, Gamhewage GM, Fall IS. One year of pandemic learning response: benefits of massive online delivery of the World Health Organization’s technical guidance. JMIR Public Health and Surveillance. 2021;7(4):e28945.

15. Utunen H, George R, Ndiaye N, Tokar A, Attias M, Gamhewage G. Delivering WHO’s life-saving information in real-time during a pandemic through an online learning platform: evidence from global use. Public Health and Informatics: IOS Press; 2021. p. 969-73.

16. Utunen H, Attias M, George R, O'Connell G, Tokar A. Learning multiplier effect of OpenWHO. org: use of online learning materials beyond the platform/Effet multiplicateur d'apprentissage de OpenWHO. org: utilisation de materiels d'apprentissage en ligne audela de la plateforme. Weekly Epidemiological Record. 2022;97(1-2):1-8.

17. Utunen H, Mattar L, Piroux C, Ndiaye N, Christen P, Attias M. Superusers of self-paced online learning on OpenWHO. 2022.

18. Utunen H, Ndiaye N, Attias M, Mattar L, Tokar A, Gamhewage G. Multilingual Approach to COVID-19 Online Learning Response on OpenWHO. org. Informatics and Technology in Clinical Care and Public Health. 2022;289:192.

19. Utunen H, Appuhamy R, Attias M, Ndiaye N, George R, Arabi E, et al. Observations from three years of online pandemic learning response on OpenWHO. The International Journal of Information and Learning Technology. 2023;40(5):527-40.

20. Utunen H, Staubitz T, George R, Zhao YU, Serth S, Tokar A. Scale Up Multilingualism in Health Emergency Learning: Developing an Automated Transcription and Translation Tool. Caring is Sharing–Exploiting the Value in Data for Health and Innovation: IOS Press; 2023. p. 408-12.

21. Utunen H, Tokar A, Dancante M, Piroux C. Online learning for WHO priority diseases with pandemic potential: evidence from existing courses and preparing for Disease X. Archives of Public Health. 2023;81(1):61.
