# Supplementary material for: Knowledge attributes of public health management information systems used in health emergencies: a scoping review
Source: Front Public Health. 2025 Mar 20;12:1458867. doi: 10.3389/fpubh.2024.1458867 (PMC11969037; doi:10.3389/fpubh.2024.1458867)
Supplement: SUPPLEMENTARY DATA SHEET 3 — Supplementary Tables C1 to C9. [file Data_Sheet_3.zip › SupplementaryTables_C1_C9_KnowledgeAttributesPerHMIS/SupplementaryTable_C1_Mode_Type.docx]

**Supplementary table C1: Literary sources for knowledge attributes of HMIS reviewed in the study – mode/type.**

|  | **IMS** | **Tacit-reasoning knowledge** | **Explicit- data and information (descriptive knowledge)** |
| --- | --- | --- | --- |
|  | TACIT Knowledge containing IMS | | |
|  | GPHIN | (Dion et al., 2015; Mykhalovskiy & Weir, 2006) |  |
|  | GLEWS | (Kshirsagar et al., 2013; Tekola et al., 2017; Thakur, 2022) |  |
|  | HealthMap | (Ahmed et al., 2015; Brownstein & Freifeld, 2007; Brownstein et al., 2008; Chen et al., 2010; Nelson R, 2008) (Sonricker et al., 2010) |  |
|  | OpenWHO | (George et al., 2022; Rohloff et al., 2018; Utunen et al., 2022; Utunen, Staubitz, et al., 2023; Utunen, Tokar, et al., 2023) |  |
|  | ProMED | (Carrion & Madoff, 2017; Chang et al., 2022; Yan et al., 2017) |  |
|  | Telemedicine | (Wang et al., 2020) (Ye, 2020) |  |
|  | mHealth | (Varshney, 2014)  (Dehling et al., 2015; Vahidi et al., 2021) |  |
|  | EXPLICIT Knowledge containing IMS | | |
|  | COVID-19 |  | (Ahmed et al., 2020; Allan et al., 2022; Sulaiman et al., 2020) |
|  | EOC |  | (Davis, 2002) (World Health Organization, 2014)  (Clark et al., 2011; Hood, 2022) (Bousso, 2019; Xu & Li, 2015) (Callan, 2020; Ma et al., 2020; Su et al., 2017) |
|  | HDX |  | (Abuoda et al., 2021; Charniga et al., 2021a, 2021b; Gao et al., 2020; Gibbs et al., 2022; Hierink et al., 2022; MacPherson et al., 2023; Metwally et al., 2023; Moulds et al., 2022a, 2022b, 2022c; Nyabinwa et al., 2020; Nyakarahuka et al., 2023; Ross et al., 2022; Yan et al., 2022) |
|  | DHIS |  | (Dehnavieh et al., 2018; DHIS2, nd) |
|  | GIS |  | (Davenhall & Kinabrew, 2012; Fradelos et al., 2014; Gülden et al., 2004; Jin et al.; Kisiala et al., 2022; Liberg, 2018; Liu & Guo; Maier & Eisner, 2017; McGregor et al., 2005; Pundt et al., 2010; Rocha et al., 2013; Schuler et al., 2022; Tao & Wu; Tsai et al., 2012; Wang et al.; Yu & Liu; Zhen et al.) |
|  | GHO |  | (Aggarwal et al., 2021; Ampofo & Boateng, 2020; Anderson et al., 2016; Cui et al., 2021; Forsea et al., 2014; Gomez, 2021; Huang et al., 2023; Huang et al., 2024; Huang et al., 2022; Huang et al., 2021; Jiang et al., 2023; Juul & Hemmingsson, 2015; Krishnamoorthy et al., 2021; Liu et al., 2017; Lu et al., 2021; Mabaso et al., 2018; Maia et al., 2023a, 2023b; Nazir et al., 2019; Onagbiye et al., 2023; Patel et al., 2015; Rodi et al., 2022; Shah et al., 2017; Stefko et al., 2017; Swarnamali et al., 2022; Tidman et al., 2023; van Rensburg et al., 2022; Vardell, 2020; Wang et al., 2024; Wu & Reynolds, 2023; Yesilaydin & Uslu, 2022) |

**References**

Abuoda, G., Hendrix, C., & Campo, S. (2021). Automatic Tag Recommendation for the UN Humanitarian Data Exchange. BIRDS+ WEPIR@ CHIIR,

Aggarwal, P., Muddasani, S., & Fleischer, A. B., Jr. (2021). Sanitation, Obesity, and Low Body Mass Index as Risk Factors for Bacterial Skin Infections [Article]. *JOURNAL OF CUTANEOUS MEDICINE AND SURGERY*, *25*(3), 293-297, Article 1203475420988857. <https://doi.org/10.1177/1203475420988857>

Ahmed, K., Bukhari, M. A., Mlanda, T., Kimenyi, J. P., Wallace, P., Lukoya, C. O., Hamblion, E. L., & Impouma, B. (2020). Novel approach to support rapid data collection, management, and visualization during the COVID-19 outbreak response in the world health organization African region: development of a data summarization and visualization tool. *JMIR Public Health and Surveillance*, *6*(4), e20355.

Ahmed, S. S., Oviedo-Orta, E., Mekaru, S. R., Freifeld, C. C., Tougas, G., & Brownstein, J. S. (2015). Surveillance for <i>Neisseria meningitidis</i> Disease Activity and Transmission Using Information Technology [Article]. *PLOS ONE*, *10*(5), Article e0127406. <https://doi.org/10.1371/journal.pone.0127406>

Allan, M., Lièvre, M., Laurenson-Schafer, H., de Barros, S., Jinnai, Y., Andrews, S., Stricker, T., Formigo, J. P., Schultz, C., & Perrocheau, A. (2022). The World Health Organization COVID-19 surveillance database. *International journal for equity in health*, *21*(Suppl 3), 167.

Ampofo, A. G., & Boateng, E. B. (2020). Beyond 2020: Modelling obesity and diabetes prevalence [Article]. *DIABETES RESEARCH AND CLINICAL PRACTICE*, *167*, Article 108362. <https://doi.org/10.1016/j.diabres.2020.108362>

Anderson, C. L., Becher, H., & Winkler, V. (2016). Tobacco Control Progress in Low and Middle Income Countries in Comparison to High Income Countries [Article]. *INTERNATIONAL JOURNAL OF ENVIRONMENTAL RESEARCH AND PUBLIC HEALTH*, *13*(10), Article 1039. <https://doi.org/10.3390/ijerph13101039>

Bousso, A. (2019). Health emergency operation centers implementation challenges in Africa [Article]. *PAN AFRICAN MEDICAL JOURNAL*, *33*, Article 171. <https://doi.org/10.11604/pamj.2019.33.171.17890>

Brownstein, J. S., & Freifeld, C. (2007). HealthMap: the development of automated real-time internet surveillance for epidemic intelligence. *Weekly releases (1997–2007)*, *12*(48), 3322.

Brownstein, J. S., Freifeld, C. C., Reis, B. Y., & Mandl, K. D. (2008). Surveillance Sans Frontières: Internet-Based Emerging Infectious Disease Intelligence and the HealthMap Project. *PLOS Medicine*, *5*(7), e151. <https://doi.org/10.1371/journal.pmed.0050151>

Callan, T. (2020). Emergency operations centres: models and core principles [Article]. *REVUE SCIENTIFIQUE ET TECHNIQUE-OFFICE INTERNATIONAL DES EPIZOOTIES*, *39*(2), 399-405. <https://doi.org/10.20506/rst.39.2.3091>

Carrion, M., & Madoff, L. C. (2017). ProMED-mail: 22 years of digital surveillance of emerging infectious diseases. *International Health*, *9*(3), 177-183. <https://doi.org/10.1093/inthealth/ihx014>

Chang, Y. C., Chiu, Y. W., & Chuang, T. W. (2022). Linguistic Pattern-Infused Dual-Channel Bidirectional Long Short-term Memory With Attention for Dengue Case Summary Generation From the Program for Monitoring Emerging Diseases-Mail Database: Algorithm Development Study [Article]. *JMIR PUBLIC HEALTH AND SURVEILLANCE*, *8*(7), Article e34583. <https://doi.org/10.2196/34583>

Charniga, K., Cucunuba, Z. M., Walteros, D. M., Mercado, M., Prieto, F., Ospina, M., Nouvellet, P., & Donnelly, C. A. (2021a). *Maps of ZIKV-associated neurological complications by department in Colombia*. <https://doi.org/10.1371/journal.pone.0252236.g006>

Charniga, K., Cucunuba, Z. M., Walteros, D. M., Mercado, M., Prieto, F., Ospina, M., Nouvellet, P., & Donnelly, C. A. (2021b). *Maps of ZVD incidence in Colombia*. <https://doi.org/10.1371/journal.pone.0252236.g002>

Chen, H., Zeng, D., Yan, P., Chen, H., Zeng, D., & Yan, P. (2010). HealthMap. *Infectious Disease Informatics: Syndromic Surveillance for Public Health and BioDefense*, 183-186.

Clark, A., Hooper, B., & Gibbs, J. (2011). Emergency operation centers or hardened command post: When, where, and how? *Journal of Chemical Health & Safety*, *18*(3), 10-14.

Cui, C. L., Dornisch, A. M., Umlauf, A. E., Cuomo, R. E., Murphy, J. D., & Lopez, N. E. (2021). International Socioeconomic Predictors of Colon and Rectal Cancer Mortality: Is Colorectal Cancer a First World Problem? [Article]. *JCO GLOBAL ONCOLOGY*, *7*, 1659-1667. <https://doi.org/10.1200/GO.21.00307>

Davenhall, W. F., & Kinabrew, C. (2012). GIS in health and human services. *Springer handbook of geographic information*, 557-578.

Davis, S. C. (2002). Virtual emergency operations centers. *Risk Management*, *49*(7), 46.

Dehling, T., Gao, F., Schneider, S., & Sunyaev, A. (2015). Exploring the far side of mobile health: information security and privacy of mobile health apps on iOS and Android. *JMIR mHealth and uHealth*, *3*(1), e3672.

Dehnavieh, R., Haghdoost, A., Khosravi, A., Hoseinabadi, F., Rahimi, H., Poursheikhali, A., Khajehpour, N., Khajeh, Z., Mirshekari, N., Hasani, M., Radmerikhi, S., Haghighi, H., Mehrolhassani, M. H., Kazemi, E., & Aghamohamadi, S. (2018). The District Health Information System (DHIS2): A literature review and meta-synthesis of its strengths and operational challenges based on the experiences of 11 countries. *Health Information Management Journal*, *48*(2), 62-75. <https://doi.org/10.1177/1833358318777713>

DHIS2. (nd). About DHIS. <https://dhis2.org/about/>

Dion, M., AbdelMalik, P., & Mawudeku, A. (2015). Big Data and the Global Public Health Intelligence Network (GPHIN). *Can Commun Dis Rep*, *41*(9), 209-214. <https://doi.org/10.14745/ccdr.v41i09a02>

Forsea, A. M., del Marmol, V., Stratigos, A., & Geller, A. C. (2014). Melanoma prognosis in Europe: far from equal [Article]. *BRITISH JOURNAL OF DERMATOLOGY*, *171*(1), 179-182. <https://doi.org/10.1111/bjd.12923>

Fradelos, E. C., Papathanasiou, I. V., Mitsi, D., Tsaras, K., Kleisiaris, C. F., & Kourkouta, L. (2014). Health based geographic information systems (GIS) and their applications. *Acta Informatica Medica*, *22*(6), 402.

Gao, W., Sanna, M., Tsai, M. K., & Wen, C. P. (2020). *Percentage of COVID-19 infected healthcare workers in severe conditions in different geographic locations and over three ten-day periods, based on symptom onset dates*. <https://doi.org/10.1371/journal.pone.0233255.g002>

George, R., Utunen, H., Ndiaye, N., Tokar, A., Mattar, L., Piroux, C., & Gamhewage, G. (2022). Ensuring equity in access to online courses: Perspectives from the WHO health emergency learning response. *World Medical & Health Policy*, *14*(2), 413-427.

Gibbs, H., Liu, Y., Abbott, S., Baffoe-Nyarko, I., Laryea, D. O., Akyereko, E., Kuma-Aboagye, P., Asante, I. A., Mitja, O., Ampofo, W., Asiedu-Bekoe, F., Marks, M., & Eggo, R. M. (2022). *Estimates of Rt in individual districts*. <https://doi.org/10.1371/journal.pgph.0000502.g003>

Gomez, E. J. (2021). Getting to the root of the problem: the international and domestic politics of junk food industry regulation in Latin America [Article]. *HEALTH POLICY AND PLANNING*, *36*(10), 1521-1533. <https://doi.org/10.1093/heapol/czab100>

Gülden, B., Mumcuoglu, E., & Baykal, N. (2004, 2004). *A GIS system for ambulatory transportation* [Proceedings Paper]. Proceedings of the Second IASTED International Conference on Biomedical Engineering,

Hierink, F., Margutti, J., Van Den Homberg, M., & Ray, N. (2022). *Overview of all results*. <https://doi.org/10.1371/journal.pntd.0009262.g001>

Hood, S. N. (2022). *Understanding Emergency Operations Center Organization and Operations in a FEMA Region* Capella University].

Huang, J., Chan, E. O.-T., Liu, X., Lok, V., Ngai, C. H., Zhang, L., Xu, W., Zheng, Z.-J., Chiu, P. K.-F., Vasdev, N., Enikeev, D., Shariat, S. F., Ng, C.-F., Teoh, J. Y.-C., & Wong, M. C. S. (2023). Global Trends of Prostate Cancer by Age, and Their Associations With Gross Domestic Product (GDP), Human Development Index (HDI), Smoking, and Alcohol Drinking [Article]. *CLINICAL GENITOURINARY CANCER*, *21*(4), E261-+. <https://doi.org/10.1016/j.clgc.2023.02.003>

Huang, J., Chan, S. C., Pang, W. S., Liu, X., Zhang, L., Lucero-Prisno Iii, D. E., Xu, W., Zheng, Z.-J., Ng, A. C.-F., Necchi, A., Spiess, P. E., Teoh, J. Y.-C., Wong, M. C. S., & Global Soc Rare Genitourinary, T. (2024). Incidence, risk factors, and temporal trends of penile cancer: a global population-based study [Article]. *BJU INTERNATIONAL*, *133*(3), 314-323. <https://doi.org/10.1111/bju.16224>

Huang, J., Leung, D. K.-W., Chan, E. O.-T., Lok, V., Leung, S., Wong, I., Lao, X.-Q., Zheng, Z.-J., Chiu, P. K.-F., Ng, C.-F., Wong, J. H.-M., Volpe, A., Merseburger, A. S., Powles, T., Teoh, J. Y.-C., & Wong, M. C. S. (2022). A Global Trend Analysis of Kidney Cancer Incidence and Mortality and Their Associations with Smoking, Alcohol Consumption, and Metabolic Syndrome [Article]. *EUROPEAN UROLOGY FOCUS*, *8*(1), 200-209. <https://doi.org/10.1016/j.euf.2020.12.020>

Huang, J., Lok, V., Ngai, C. H., Zhang, L., Yuan, J., Lao, X. Q., Ng, K., Chong, C., Zheng, Z.-J., & Wong, M. C. S. (2021). Worldwide Burden of, Risk Factors for, and Trends in Pancreatic Cancer [Article]. *GASTROENTEROLOGY*, *160*(3), 744-754. <https://doi.org/10.1053/j.gastro.2020.10.007>

Jiang, B., Wu, T., Liu, W., Liu, G., & Lu, P. (2023). Changing Trends in the Global Burden of Cataract Over the Past 30 Years: Retrospective Data Analysis of the Global Burden of Disease Study 2019 [Article]. *JMIR PUBLIC HEALTH AND SURVEILLANCE*, *9*, Article e47349. <https://doi.org/10.2196/47349>

Jin, L., Li, Q., & Niu, Y. *Emergent public health event processing and displaying method, involves determining public sanitary event corresponding to burst response level, and determining burst public health event response level by electronic map display* CN110084730-A).

Juul, F., & Hemmingsson, E. (2015). Trends in consumption of ultra-processed foods and obesity in Sweden between 1960 and 2010 [Article]. *PUBLIC HEALTH NUTRITION*, *18*(17), 3096-3107. <https://doi.org/10.1017/S1368980015000506>

Kisiala, W., Racka, I., & Suszynska, K. (2022). Population Access to Hospital Emergency Departments: The Spatial Analysis in Public Health Research [Article]. *INTERNATIONAL JOURNAL OF ENVIRONMENTAL RESEARCH AND PUBLIC HEALTH*, *19*(3), Article 1437. <https://doi.org/10.3390/ijerph19031437>

Krishnamoorthy, Y., Nagarajan, R., Rajaa, S., Majella, M. G., Murali, S., & Jayaseelan, V. (2021). Progress of South East Asian Region countries towards achieving interim End TB strategy targets for TB incidence and mortality: a modelling study [Article]. *PUBLIC HEALTH*, *198*, 9-16. <https://doi.org/10.1016/j.puhe.2021.06.021>

Kshirsagar, D., Savalia, C., Kalyani, I., Kumar, R., & Nayak, D. (2013). Disease alerts and forecasting of zoonotic diseases: an overview. *Veterinary World*, *6*(11), 889.

Liberg, R. B. (2018). USING GEOGRAPHIC INFORMATION SYSTEMS IN RURAL EMERGENCY MEDICAL SERVICES: REDUCING RESPONSE TIMES BY REALLOCATING RESOURCES [Meeting Abstract]. *JOURNAL OF INVESTIGATIVE MEDICINE*, *66*(1), 104-104. <https://doi.org/10.1136/jim-2017-000663.94>

Liu, J., & Guo, M. *Infectious disease monitoring and pre-warning system for use in public health emergencies, has data storage center for storing and collecting data from hospital diagnosis monitoring module, drugstore medicine sale monitoring module, and infectious disease history analysis data* CN117174332-A).

Liu, J. X., Goryakin, Y., Maeda, A., Bruckner, T., & Scheffler, R. (2017). Global Health Workforce Labor Market Projections for 2030 [Article]. *HUMAN RESOURCES FOR HEALTH*, *15*, Article 11. <https://doi.org/10.1186/s12960-017-0187-2>

Lu, B., Li, N., Luo, C.-Y., Cai, J., Lu, M., Zhang, Y.-H., Chen, H.-D., & Dai, M. (2021). Colorectal cancer incidence and mortality: the current status, temporal trends and their attributable risk factors in 60 countries in 2000-2019 [Article]. *CHINESE MEDICAL JOURNAL*, *134*(16), 1941-1951. <https://doi.org/10.1097/CM9.0000000000001619>

Ma, J., Huang, Y., & Zheng, Z.-J. (2020). Leveraging the Public Health Emergency Operation Center (PHEOC) for pandemic response: opportunities and challenges [Journal Article

Review]. *Global health journal (Amsterdam, Netherlands)*, *4*(4), 118-120. <https://doi.org/10.1016/j.glohj.2020.11.004>

Mabaso, M. L. H., Zama, T. P., Mlangeni, L., Mbiza, S., & Mkhize-Kwitshana, Z. L. (2018). Association between the Human Development Index and Millennium Development Goals 6 Indicators in Sub-Saharan Africa from 2000 to 2014: Implications for the New Sustainable Development Goals [Article]. *JOURNAL OF EPIDEMIOLOGY AND GLOBAL HEALTH*, *8*(1-2), 77-81. <https://doi.org/10.2991/j.jegh.2018.09.001>

MacPherson, E. E., Mankhomwa, J., Dixon, J., Pongolani, R., Phiri, M., Feasey, N., Obyrne, T., Tolhurst, R., & MacPherson, P. (2023). *Location of households sampled for interview*. <https://doi.org/10.1371/journal.pgph.0001946.g001>

Maia, C., Conceicao, C., Pereira, A., Rocha, R., Ortuno, M., Munoz, C., Jumakanova, Z., Perez-Cutillas, P., Ozbel, Y., Toz, S., Baneth, G., Monge-Maillo, B., Gasimov, E., Van Der Stede, Y., Torres, G., Gossner, C. M., & Berriatua, E. (2023a). *Annual cumulative incidence per 100,000 population of autochthonous human cutaneous leishmaniasis in European countries between 2005 and 2020*. <https://doi.org/10.1371/journal.pntd.0011497.g003>

Maia, C., Conceicao, C., Pereira, A., Rocha, R., Ortuno, M., Munoz, C., Jumakanova, Z., Perez-Cutillas, P., Ozbel, Y., Toz, S., Baneth, G., Monge-Maillo, B., Gasimov, E., Van Der Stede, Y., Torres, G., Gossner, C. M., & Berriatua, E. (2023b). *Annual cumulative incidence per 100,000 population of human autochthonous visceral leishmaniasis in European countries between 2005 and 2020*. <https://doi.org/10.1371/journal.pntd.0011497.g002>

Maier, N. M., & Eisner, G. R. (2017). *Method for locating internet of things network devices e.g. baby monitors, during e.g. health event, involves displaying current physical geographic location for network device for desired emergency response agencies on graphical map* US2017238129-A1

US10511950-B2).

McGregor, J., Hanlon, N., Emmons, S., Voaklander, D., & Kelly, K. (2005). If all ambulances could fly: putting provincial standards of emergency care access to the test in Northern British Columbia [Journal Article

Research Support, Non-U.S. Gov't]. *Canadian journal of rural medicine : the official journal of the Society of Rural Physicians of Canada = Journal canadien de la medecine rurale : le journal officiel de la Societe de medecine rurale du Canada*, *10*(3), 163-168.

Metwally, A. M., Nassar, M. S., El-Din, E. M., Abdallah, A. M., Khadr, Z., Abouelnaga, M. W., Ashaat, E. A., El-Saied, M. M., Elwan, A. M., Bassiouni, R. I., Monir, Z. M., Badawy, H. Y., Dewdar, E. M., El-Hariri, H. M., Aboulghate, A., Hanna, C., Rabah, T. M., Mohsen, A., & Elabd, M. A. (2023). *Map of the 27 Egyptians governorates distributed within the four geographic regions (adapted using data from the Humanitarian Data Exchange under the CC BY-IGO license [18]*. <https://doi.org/10.1371/journal.pone.0287315.g001>

Moulds, S., Chan, A. C. H., Tetteh, J. D., Bixby, H., Owusu, G., Agyei-Mensah, S., Ezzati, M., Buytaert, W., & Templeton, M. R. (2022a). *Local Indicators of Spatial Association clusters for Ghana in 2010, 2013 and 2017*. <https://doi.org/10.1371/journal.pone.0265167.g006>

Moulds, S., Chan, A. C. H., Tetteh, J. D., Bixby, H., Owusu, G., Agyei-Mensah, S., Ezzati, M., Buytaert, W., & Templeton, M. R. (2022b). *Most common source of drinking water in Ghanas 170 districts*. <https://doi.org/10.1371/journal.pone.0265167.g004>

Moulds, S., Chan, A. C. H., Tetteh, J. D., Bixby, H., Owusu, G., Agyei-Mensah, S., Ezzati, M., Buytaert, W., & Templeton, M. R. (2022c). *Percentage of households drinking sachet water in Ghanas 170 districts*. <https://doi.org/10.1371/journal.pone.0265167.g005>

Mykhalovskiy, E., & Weir, L. (2006). The Global Public Health Intelligence Network and early warning outbreak detection: a Canadian contribution to global public health. *Canadian journal of public health*, *97*, 42-44.

Nazir, M. A., Al-Ansari, A., Abbasi, N., & Almas, K. (2019). Global Prevalence of Tobacco Use in Adolescents and Its Adverse Oral Health Consequences [Journal Article]. *Open access Macedonian journal of medical sciences*, *7*(21), 3659-3666. <https://doi.org/10.3889/oamjms.2019.542>

Nelson R. (2008). HealthMap: the future of infectious diseases surveillance? *The Lancet Infectious Diseases*, *8*(10), 596.

Nyabinwa, P., Kashongwe, O. B., Hirwa, C. D., & Bebe, B. O. (2020). *Additional file 1 of Perception of farmers about endometritis prevention and control measures for zero-grazed dairy cows on smallholder farms in Rwanda*. <https://doi.org/10.6084/m9.figshare.12441284.v1>

Nyakarahuka, L., Kyondo, J., Telford, C., Whitesell, A., Tumusiime, A., Mulei, S., Baluku, J., Cossaboom, C. M., Cannon, D. L., Montgomery, J. M., Lutwama, J. J., Nichol, S. T., Balinandi, S. K., Klena, J. D., & Shoemaker, T. R. (2023). *Sampled districts and their corresponding seroprevalence of Crimean-Congo hemorrhagic fever virus IgG antibodies in cattle, sheep and goats (Open-source shapefiles for Uganda district boundaries were downloaded from the Humanitarian Data Exchange (Humanitarian Data Exchange, 2020) and water bodies files from the World Bank website (The World Bank, 2022))*. <https://doi.org/10.1371/journal.pone.0288587.g001>

Onagbiye, S., Ricci, H., Bester, P., & Ricci, C. (2023). Sedentariness and overweight in relation to mortality in sub-Saharan Africa. A mediation analysis based on the World Health Organization-Global Health Observatory data repository [Article]. *JOURNAL OF PUBLIC HEALTH IN AFRICA*, *14*(4), Article 2155. <https://doi.org/10.4081/jphia.2023.2155>

Patel, H., Kielhorn, A., Yurgin, N., & Hernandez, A. F. (2015). Years of Life Lost Due to Heart Failure in the United States (US) [Meeting Abstract]. *CIRCULATION*, *132*.

Pundt, H., Spangenberg, T., & Weinkauf, R. (2010, 2010). *WEB-BASED AND CONTEXT-SENSITIVE, MOBILE GEO-TOOLS TO SUPPORT SPATIAL DECISION MAKING IN HEALTH AND EMERGENCY MANAGEMENT* [Proceedings Paper]. HEALTHINF 2010: PROCEEDINGS OF THE THIRD INTERNATIONAL CONFERENCE ON HEALTH INFORMATICS,

Rocha, C. M., Kruger, E., McGuire, S., & Tennant, M. (2013). The geographic distribution of patients seeking emergency dental care at the Royal Dental Hospital of Melbourne, Australia [Article]. *COMMUNITY DENTAL HEALTH*, *30*(3), 149-154. <https://doi.org/10.1922/CDH_3124Kruger06>

Rodi, P., Obermeyer, W., Pablos-Mendez, A., Gori, A., & Raviglione, M. C. (2022). *Overview of DAH, number of deaths, mortality rates, and health expenditures*. <https://doi.org/10.1371/journal.pmed.1003873.g001>

Rohloff, T., Utunen, H., Renz, J., Zhao, Y., Gamhewage, G., & Meinel, C. (2018). OpenWHO: Integrating Online Knowledge Transfer into Health Emergency Response. EC-TEL (Practitioner Proceedings),

Ross, Y. B., Hoque, M., Blanton, J. D., Kennedy, E. D., Rana, M. S., Tahmina, S., Bonaparte, S., Head, J. R., & Wallace, R. M. (2022). *Map of Bangladesh survey sites and the distribution of household surveys by survey site*. <https://doi.org/10.1371/journal.pntd.0010634.g001>

Schuler, F., Ma, M., & Perkins, J. (2022). *System for determining priority discrepancies between audio data and records data, has electronic computing device for generating geographical map that includes priority discrepancies, and is configured for display on display device* WO2022213023-A1

US2022318278-A1).

Shah, N. D., Cruz-Lemini, M., Stein, E., Abraldes, J., Altamirano, J., & Bataller, R. (2017). COLDER WEATHER AND FEWER SUNLIGHT HOURS INCREASE THE WEIGHT OF ALCOHOL AS A CAUSE OF CIRRHOSIS WORLDWIDE [Meeting Abstract]. *GASTROENTEROLOGY*, *152*(5), S942-S943. <https://doi.org/10.1016/S0016-5085(17)33209-2>

Sonricker, A. L., Freifeld, C. C., Keller, M., & Brownstein, J. S. (2010). HealthMap. In *Biosurveillance* (pp. 133-146). Chapman and Hall/CRC.

Stefko, R., Jencova, S., Litavcova, E., & Vasanicova, P. (2017). MANAGEMENT AND FUNDING OF THE HEALTHCARE SYSTEM [Article]. *POLISH JOURNAL OF MANAGEMENT STUDIES*, *16*(2), 266-277. <https://doi.org/10.17512/pjms.2017.16.2.23>

Su, Y.-F., Wu, C.-H., & Lee, T.-F. (2017). PUBLIC HEALTH EMERGENCY RESPONSE IN TAIWAN [Article]. *HEALTH SECURITY*, *15*(2), 137-143. <https://doi.org/10.1089/hs.2016.0108>

Sulaiman, N., Abid, S. K., Chan, S. W., Nazir, U., Mahmud, N. P. N., Latib, S., Hafidz, H., Shahlal, S., Sapuan, S., & Fernando, T. (2020). Geospatial dashboards for mapping and tracking of novel coronavirus pandemic. Proc. Int. Conf. Ind. Eng. Oper. Manag,

Swarnamali, H., Jayawardena, R., Chourdakis, M., & Ranasinghe, P. (2022). Is the proportion of per capita fat supply associated with the prevalence of overweight and obesity? an ecological analysis [Article]. *BMC NUTRITION*, *8*(1), Article 4. <https://doi.org/10.1186/s40795-021-00496-2>

Tao, Y., & Wu, P. *Emergency evacuation method for evacuating people urgently to ensure safety of life and property of people, based on geographic information system (GIS) in event type such as natural disaster type, involves sending emergency evacuation information to target mobile device* CN117082450-A).

Tekola, B., Myers, L., Lubroth, J., Plee, L., Calistri, P., & Pinto, J. (2017). International health threats and global early warning and response mechanisms [Article]. *REVUE SCIENTIFIQUE ET TECHNIQUE-OFFICE INTERNATIONAL DES EPIZOOTIES*, *36*(2), 657-670. <https://doi.org/10.20506/rst.36.2.2683>

Thakur, S. D. (2022). Early Warning Systems, Disease Management, and Biosecurity in Disasters. In *Management of Animals in Disasters* (pp. 25-37). Springer.

Tidman, R., Kanankege, K. S. T., Bangert, M., & Abela-Ridder, B. (2023). Global prevalence of 4 neglected foodborne trematodes targeted for control by WHO: A scoping review to highlight the gaps [Review]. *PLOS NEGLECTED TROPICAL DISEASES*, *17*(3), Article e0011073. <https://doi.org/10.1371/journal.pntd.0011073>

Tsai, M.-K., Lee, Y.-C., Lu, C.-H., Chen, M.-H., Chou, T.-Y., & Yau, N.-J. (2012). Integrating geographical information and augmented reality techniques for mobile escape guidelines on nuclear accident sites [Article]. *JOURNAL OF ENVIRONMENTAL RADIOACTIVITY*, *109*, 36-44. <https://doi.org/10.1016/j.jenvrad.2011.12.025>

Utunen, H., Ndiaye, N., Attias, M., Mattar, L., Tokar, A., & Gamhewage, G. (2022). Multilingual Approach to COVID-19 Online Learning Response on OpenWHO. org. *Informatics and Technology in Clinical Care and Public Health*, *289*, 192.

Utunen, H., Staubitz, T., George, R., Zhao, Y. U., Serth, S., & Tokar, A. (2023). Scale Up Multilingualism in Health Emergency Learning: Developing an Automated Transcription and Translation Tool. In *Caring is Sharing–Exploiting the Value in Data for Health and Innovation* (pp. 408-412). IOS Press.

Utunen, H., Tokar, A., Dancante, M., & Piroux, C. (2023). Online learning for WHO priority diseases with pandemic potential: evidence from existing courses and preparing for Disease X. *Archives of Public Health*, *81*(1), 61. <https://doi.org/10.1186/s13690-023-01080-9>

Vahidi, H., Taleai, M., Yan, W., & Shaw, R. (2021). Digital Citizen Science for Responding to COVID-19 Crisis: Experiences from Iran [Article]. *INTERNATIONAL JOURNAL OF ENVIRONMENTAL RESEARCH AND PUBLIC HEALTH*, *18*(18), Article 9666. <https://doi.org/10.3390/ijerph18189666>

van Rensburg, B. J., Kotze, C., Moxley, K., Subramaney, U., Zingela, Z., & Seedat, S. (2022). Profile of the current psychiatrist workforce in South Africa: establishing a baseline for human resource planning and strategy [Article]. *HEALTH POLICY AND PLANNING*, *37*(4), 492-504. <https://doi.org/10.1093/heapol/czab144>

Vardell, E. (2020). Global health observatory data repository. *Medical reference services quarterly*, *39*(1), 67-74.

Varshney, U. (2014). Mobile health: Four emerging themes of research. *Decision Support Systems*, *66*, 20-35.

Wang, M., Maimaitiming, M., Zhao, Y., Jin, Y., & Zheng, Z. J. (2024). Global trends in deaths and disability-adjusted life years of diabetes attributable to second-hand smoke and the association with smoke-free policies [Article]. *PUBLIC HEALTH*, *228*, 18-27. <https://doi.org/10.1016/j.puhe.2023.12.025>

Wang, Y., Li, B., & Liu, L. (2020). Telemedicine experience in China: our response to the pandemic and current challenges. *Frontiers in Public Health*, *8*, 549669.

Wang, Z., Li, H., Song, J., Gong, X., Chen, N., Song, C., & Lu, Y. *Public health event emergency medical facility addressing method based on point of interest data, involves establishing a file geographic information database by using a file geographic information system software* CN112232599-A).

World Health Organization. (2014). A systematic review of public health emergency operations centres (EOC): December 2013. <https://www.who.int/publications/i/item/WHO-HSE-GCR-2014.1>

Wu, T. J., & Reynolds, M. M. (2023). Trachoma, the world's leading infectious cause of blindness: The remaining gap in care and access to basic handwashing facilities [Article]. *EUROPEAN JOURNAL OF OPHTHALMOLOGY*, *33*(4), 1576-1582. <https://doi.org/10.1177/11206721231154295>

Xu, M., & Li, S.-X. (2015). Analysis of good practice of public health Emergency Operations Centers. *Asian Pacific journal of tropical medicine*, *8*(8), 677-682.

Yan, L. D., McNairy, M. L., Devieux, J. G., Pierre, J. L., Dade, E., Sufra, R., Gerber, L. M., Roberts, N., St Preux, S., Malebranche, R., Metz, M., Tymejczyk, O., Nash, D., Deschamps, M., Safford, M. M., Pape, J. W., & Rouzier, V. (2022). *Map of census blocks sampled in Haiti CVD cohort*. <https://doi.org/10.1371/journal.pgph.0000503.g001>

Yan, S. J., Chughtai, A. A., & Macintyre, C. R. (2017). Utility and potential of rapid epidemic intelligence from internet-based sources [Review]. *INTERNATIONAL JOURNAL OF INFECTIOUS DISEASES*, *63*, 77-87. <https://doi.org/10.1016/j.ijid.2017.07.020>

Ye, J. (2020). The role of health technology and informatics in a global public health emergency: practices and implications from the COVID-19 pandemic. *JMIR medical informatics*, *8*(7), e19866.

Yesilaydin, G., & Uslu, E. (2022). The relationship between life expectancy and mental health systems in European countries [Article]. *JOURNAL OF PSYCHIATRIC NURSING*, *13*(3), 199-204. <https://doi.org/10.14744/phd.2022.70456>

Yu, Z., & Liu, P. *Method for locating geographical position of graph convolution network model based on attention aggregate, involves inputting result obtained by training graph convolution network model to geographic position predictor of multilayer sensor* CN116166865-A).

Zhen, W., Li, C., Chen, R., Wei, Z., Chen, T., & Yan, J. *Multi-scene city emergency sampling site addressing optimization method for city public health safety technical field, involves obtaining mobile phone signaling data of target city, interest point, and basic geographic information data* CN115860213-A).
