# Supplementary material for: Knowledge attributes of public health management information systems used in health emergencies: a scoping review
Source: Front Public Health. 2025 Mar 20;12:1458867. doi: 10.3389/fpubh.2024.1458867 (PMC11969037; doi:10.3389/fpubh.2024.1458867)
Supplement: SUPPLEMENTARY DATA SHEET 3 — Supplementary Tables C1 to C9. [file Data_Sheet_3.zip › SupplementaryTables_C1_C9_KnowledgeAttributesPerHMIS/SupplementaryTable_C2_Source.docx]

**Supplementary table C2: Literary sources for knowledge attributes of HMIS reviewed in the study -Source.**

|  | **IMS** | **Formal and informal sources** | **Formal sources only** |
| --- | --- | --- | --- |
|  | TACIT Knowledge containing IMS | | |
|  | GPHIN | (Carter et al., 2020; Keller et al., 2009; Madoff & Li, 2014; Mykhalovskiy & Weir, 2006; Roberts & Elbe, 2017; Young et al., 2015) |  |
|  | GLEWS |  | (Arnoldi et al., 2004; Caceres, 2016; Jebara, 2004; Tekola et al., 2017) |
|  | HealthMap | (Ahmed et al., 2015; Bhatia et al., 2021; Brownstein et al., 2008; Majumder et al., 2016; Sonricker et al., 2010; Valentin et al., 2023) |  |
|  | OpenWHO | (George et al., 2022) |  |
|  | ProMED | (Hugh-Jones, 2001; Zeldenrust et al., 2008) |  |
|  | Telemedicine | (Wang et al., 2020) |  |
|  | mHealth | (Vahidi et al., 2021) |  |
|  | EXPLICIT Knowledge containing IMS | | |
|  | COVID-19 |  | (Ahmed et al., 2020) |
|  | EOC | (Becerra-Fernández et al., 2008; Callan, 2020) |  |
|  | HDX |  | (Paulus et al., 2018) |
|  | DHIS |  | (Dehnavieh et al., 2018; Odhiambo-Otieno, 2005) |
|  | GIS | (Melnick, 2002; Tzavella et al., 2018) |  |
|  | GHO |  | (Vardell, 2020) |

**References**

Ahmed, K., Bukhari, M. A., Mlanda, T., Kimenyi, J. P., Wallace, P., Lukoya, C. O., Hamblion, E. L., & Impouma, B. (2020). Novel approach to support rapid data collection, management, and visualization during the COVID-19 outbreak response in the world health organization African region: development of a data summarization and visualization tool. *JMIR Public Health and Surveillance*, *6*(4), e20355.

Ahmed, S. S., Oviedo-Orta, E., Mekaru, S. R., Freifeld, C. C., Tougas, G., & Brownstein, J. S. (2015). Surveillance for <i>Neisseria meningitidis</i> Disease Activity and Transmission Using Information Technology [Article]. *PLOS ONE*, *10*(5), Article e0127406. <https://doi.org/10.1371/journal.pone.0127406>

Arnoldi, J. M., David, M. J., Fernandez, P. J., Fischer, J. R., Frost, B., Lautner, E. A., Marsh, B. D., Taylor, M. A., Thiermann, A. B., & Torres, A. (2004). Report of the USAHA/AAVLD Committee on International Standards. PROCEEDINGS OF THE ANNUAL MEETING-UNITED STATES ANIMAL HEALTH ASSOCIATION,

Becerra-Fernández, I., Madey, G., Prietula, M., Rodríguez, D., Valerdi, R., & Wright, T. (2008). Design and development of a virtual emergency operations center for disaster management research, training, and discovery. Proceedings of the 41st Annual Hawaii International Conference on System Sciences (HICSS 2008),

Bhatia, S., Lassmann, B., Cohn, E., Desai, A. N., Carrion, M., Kraemer, M. U. G., Herringer, M., Brownstein, J., Madoff, L., Cori, A., & Nouvellet, P. (2021). Using digital surveillance tools for near real-time mapping of the risk of infectious disease spread [Article]. *NPJ DIGITAL MEDICINE*, *4*(1), Article 73. <https://doi.org/10.1038/s41746-021-00442-3>

Brownstein, J. S., Freifeld, C. C., Reis, B. Y., & Mandl, K. D. (2008). Surveillance Sans Frontières: Internet-Based Emerging Infectious Disease Intelligence and the HealthMap Project. *PLOS Medicine*, *5*(7), e151. <https://doi.org/10.1371/journal.pmed.0050151>

Caceres, P. (2016). Tracking activity to improve the sensitivity of the OIE's monitoring and early warning systems for human and animal diseases. *International Journal of Infectious Diseases*, *53*, 11. <https://doi.org/10.1016/j.ijid.2016.11.032>

Callan, T. (2020). Emergency operations centres: models and core principles [Article]. *REVUE SCIENTIFIQUE ET TECHNIQUE-OFFICE INTERNATIONAL DES EPIZOOTIES*, *39*(2), 399-405. <https://doi.org/10.20506/rst.39.2.3091>

Carter, D., Stojanovic, M., Hachey, P., Fournier, K., Rodier, S., Wang, Y., & de Bruijn, B. (2020, 2020). *Global Public Health Surveillance Using Media Reports: Redesigning GPHIN* [Proceedings Paper]. DIGITAL PERSONALIZED HEALTH AND MEDICINE,

Dehnavieh, R., Haghdoost, A., Khosravi, A., Hoseinabadi, F., Rahimi, H., Poursheikhali, A., Khajehpour, N., Khajeh, Z., Mirshekari, N., Hasani, M., Radmerikhi, S., Haghighi, H., Mehrolhassani, M. H., Kazemi, E., & Aghamohamadi, S. (2018). The District Health Information System (DHIS2): A literature review and meta-synthesis of its strengths and operational challenges based on the experiences of 11 countries. *Health Information Management Journal*, *48*(2), 62-75. <https://doi.org/10.1177/1833358318777713>

George, R., Utunen, H., Ndiaye, N., Tokar, A., Mattar, L., Piroux, C., & Gamhewage, G. (2022). Ensuring equity in access to online courses: Perspectives from the WHO health emergency learning response. *World Medical & Health Policy*, *14*(2), 413-427.

Hugh-Jones, M. (2001). Global awareness of disease outbreaks: the experience of ProMED-mail. *Public Health Reports*, *116*(Suppl 2), 27.

Jebara, K. B. (2004). Surveillance, detection and response: managing emerging diseases at national and international levels. *Rev Sci Tech*, *23*(2), 709-715.

Keller, M., Blench, M., Tolentino, H., Freifeld, C. C., Mandl, K. D., Mawudeku, A., Eysenbach, G., & Brownstein, J. S. (2009). Use of Unstructured Event-Based Reports for Global Infectious Disease Surveillance [Article]. *EMERGING INFECTIOUS DISEASES*, *15*(5), 689-695. <https://doi.org/10.3201/eid1505.081114>

Madoff, L. C., & Li, A. (2014). Web-Based Surveillance Systems for Human, Animal, and Plant Diseases [Journal Article

Review]. *Microbiology spectrum*, *2*(1), OH-0015-2012. <https://doi.org/10.1128/microbiolspec.OH-0015-2012>

Majumder, M. S., Santillana, M., Mekaru, S. R., McGinnis, D. P., Khan, K., & Brownstein, J. S. (2016). Utilizing Nontraditional Data Sources for Near Real-Time Estimation of Transmission Dynamics During the 2015-2016 Colombian Zika Virus Disease Outbreak [Journal Article]. *JMIR public health and surveillance*, *2*(1), e30-e30. <https://doi.org/10.2196/publichealth.5814>

Melnick, A. L. (2002). *Introduction to geographic information systems in public health*. Jones & Bartlett Learning.

Mykhalovskiy, E., & Weir, L. (2006). The Global Public Health Intelligence Network and early warning outbreak detection: a Canadian contribution to global public health. *Canadian journal of public health*, *97*, 42-44.

Odhiambo-Otieno, G. W. (2005). Evaluation criteria for district health management information systems: lessons from the Ministry of Health, Kenya. *International Journal of Medical Informatics*, *74*(1), 31-38. <https://doi.org/https://doi.org/10.1016/j.ijmedinf.2004.09.003>

Paulus, D., Meesters, K., & Van de Walle, B. A. (2018). Turning data into action: supporting humanitarian field workers with open data. Iscram,

Roberts, S. L., & Elbe, S. (2017). Catching the flu: Syndromic surveillance, algorithmic governmentality and global health security [Article]. *SECURITY DIALOGUE*, *48*(1), 46-62. <https://doi.org/10.1177/0967010616666443>

Sonricker, A. L., Freifeld, C. C., Keller, M., & Brownstein, J. S. (2010). HealthMap. In *Biosurveillance* (pp. 133-146). Chapman and Hall/CRC.

Tekola, B., Myers, L., Lubroth, J., Plee, L., Calistri, P., & Pinto, J. (2017). International health threats and global early warning and response mechanisms [Article]. *REVUE SCIENTIFIQUE ET TECHNIQUE-OFFICE INTERNATIONAL DES EPIZOOTIES*, *36*(2), 657-670. <https://doi.org/10.20506/rst.36.2.2683>

Tzavella, K., Fekete, A., & Fiedrich, F. (2018). Opportunities provided by geographic information systems and volunteered geographic information for a timely emergency response during flood events in Cologne, Germany. *Natural Hazards*, *91*, 29-57.

Vahidi, H., Taleai, M., Yan, W., & Shaw, R. (2021). Digital Citizen Science for Responding to COVID-19 Crisis: Experiences from Iran [Article]. *INTERNATIONAL JOURNAL OF ENVIRONMENTAL RESEARCH AND PUBLIC HEALTH*, *18*(18), Article 9666. <https://doi.org/10.3390/ijerph18189666>

Valentin, S., Boudoua, B., Sewalk, K., Arınık, N., Roche, M., Lancelot, R., & Arsevska, E. (2023). Dissemination of information in event-based surveillance, a case study of Avian Influenza. *PLOS ONE*, *18*(9), e0285341. <https://doi.org/10.1371/journal.pone.0285341>

Vardell, E. (2020). Global health observatory data repository. *Medical reference services quarterly*, *39*(1), 67-74.

Wang, Y., Li, B., & Liu, L. (2020). Telemedicine experience in China: our response to the pandemic and current challenges. *Frontiers in Public Health*, *8*, 549669.

Young, M. M., Dubeau, C., & Corazza, O. (2015). Detecting a signal in the noise: monitoring the global spread of novel psychoactive substances using media and other open-source information [Article]. *HUMAN PSYCHOPHARMACOLOGY-CLINICAL AND EXPERIMENTAL*, *30*(4), 319-326. <https://doi.org/10.1002/hup.2477>

Zeldenrust, M., Rahamat-Langendoen, J., Postma, M., & Van Vliet, J. (2008). The value of ProMED-mail for the Early Warning Committee in the Netherlands: more specific approach recommended. *Eurosurveillance*, *13*(6), 7-8.
