# Supplementary material for: Knowledge attributes of public health management information systems used in health emergencies: a scoping review
Source: Front Public Health. 2025 Mar 20;12:1458867. doi: 10.3389/fpubh.2024.1458867 (PMC11969037; doi:10.3389/fpubh.2024.1458867)
Supplement: SUPPLEMENTARY DATA SHEET 3 — Supplementary Tables C1 to C9. [file Data_Sheet_3.zip › SupplementaryTables_C1_C9_KnowledgeAttributesPerHMIS/SupplementaryTable_C3_Domain.docx]

**Supplementary table C3: Literary sources for knowledge attributes of HMIS reviewed in the study – domain.**

|  | **IMS** | **Animal and Human Health** | **Human health only** |
| --- | --- | --- | --- |
|  | TACIT Knowledge containing IMS | | |
|  | GPHIN | (Blench, 2007) (Madoff & Li, 2014; Mawudeku & Blench, 2005; Roberts & Elbe, 2017) |  |
|  | GLEWS | (Kshirsagar et al., 2013; Savelli et al., 2013; Tekola et al., 2017; Vandersmissen & Welburn, 2014) |  |
|  | HealthMap | (Freifeld et al., 2008; Nelson R, 2008) (Ahmed et al., 2015) |  |
|  | OpenWHO | (Utunen et al., 2023) |  |
|  | ProMED | (Carrion & Madoff, 2017; Yu & Madoff, 2004) |  |
|  | Telemed | (Wang et al., 2020) |  |
|  | mHealth | (Adeniyi et al., 2020; Albabtain et al., 2014; Littman-Quinn et al., 2011; Roncero et al., 2020; Tamrat & Kachnowski, 2012) |  |
|  | EXPLICIT Knowledge containing IMS | | |
|  | COVID-19 |  | (Ahmed et al., 2020) |
|  | EOC | (Callan, 2020) (Kayiwa et al., 2022) |  |
|  | HDX | (Abuoda et al., 2021) |  |
|  | DHIS | (DHIS2, nd; Farnham et al., 2020) (Manoj et al., 2013) |  |
|  | GIS | (Kaiser et al., 2003) |  |
|  | GHO | (Vardell, 2020) |  |

**References**

Abuoda, G., Hendrix, C., & Campo, S. (2021). Automatic Tag Recommendation for the UN Humanitarian Data Exchange. BIRDS+ WEPIR@ CHIIR,

Adeniyi, E. A., Awotunde, J. B., Ogundokun, R. O., Kolawole, P. O., Abiodun, M. K., & Adeniyi, A. A. (2020). Mobile health application and COVID-19: Opportunities and challenges. *Journal of Critical Reviews*, *7*(15), 3481-3488.

Ahmed, K., Bukhari, M. A., Mlanda, T., Kimenyi, J. P., Wallace, P., Lukoya, C. O., Hamblion, E. L., & Impouma, B. (2020). Novel approach to support rapid data collection, management, and visualization during the COVID-19 outbreak response in the world health organization African region: development of a data summarization and visualization tool. *JMIR Public Health and Surveillance*, *6*(4), e20355.

Ahmed, S. S., Oviedo-Orta, E., Mekaru, S. R., Freifeld, C. C., Tougas, G., & Brownstein, J. S. (2015). Surveillance for <i>Neisseria meningitidis</i> Disease Activity and Transmission Using Information Technology [Article]. *PLOS ONE*, *10*(5), Article e0127406. <https://doi.org/10.1371/journal.pone.0127406>

Albabtain, A. F., AlMulhim, D. A., Yunus, F., & Househ, M. S. (2014). The role of mobile health in the developing world: a review of current knowledge and future trends. *Journal of Selected Areas in Health Informatics*, *4*(2), 10-15.

Blench, M. (2007). Global public health intelligence network (GPHIN). Proceedings of Machine Translation Summit XI: Papers,

Callan, T. (2020). Emergency operations centres: models and core principles [Article]. *REVUE SCIENTIFIQUE ET TECHNIQUE-OFFICE INTERNATIONAL DES EPIZOOTIES*, *39*(2), 399-405. <https://doi.org/10.20506/rst.39.2.3091>

Carrion, M., & Madoff, L. C. (2017). ProMED-mail: 22 years of digital surveillance of emerging infectious diseases. *International Health*, *9*(3), 177-183. <https://doi.org/10.1093/inthealth/ihx014>

DHIS2. (nd). About DHIS. <https://dhis2.org/about/>

Farnham, A., Utzinger, J., Kulinkina, A. V., & Winkler, M. S. (2020). Using district health information to monitor sustainable development. *Bull World Health Organ*, *98*(1), 69-71. <https://doi.org/10.2471/blt.19.239970>

Freifeld, C. C., Mandl, K. D., Reis, B. Y., & Brownstein, J. S. (2008). HealthMap: global infectious disease monitoring through automated classification and visualization of Internet media reports. *Journal of the American Medical Informatics Association*, *15*(2), 150-157.

Kaiser, R., Spiegel, P. B., Henderson, A. K., & Gerber, M. L. (2003). The application of geographic information systems and global positioning systems in humanitarian emergencies: lessons learned, programme implications and future research. *Disasters*, *27*(2), 127-140.

Kayiwa, J., Homsy, J., Nelson, L. J., Ocom, F., Kasule, J. N., Wetaka, M. M., Kyazze, S., Mwanje, W., Kisakye, A., Nabunya, D., Nyirabakunzi, M., Aliddeki, D. M., Ojwang, J., Boore, A., Kasozi, S., Borchert, J., Shoemaker, T., Nabatanzi, S., Dahlke, M., . . . Makumbi, I. (2022). Establishing a Public Health Emergency Operations Center in an Outbreak-Prone Country: Lessons Learned in Uganda, January 2014 to December 2021 [Article

Early Access]. *HEALTH SECURITY*, *20*(5), 394-407. <https://doi.org/10.1089/hs.2022.0048>

Kshirsagar, D., Savalia, C., Kalyani, I., Kumar, R., & Nayak, D. (2013). Disease alerts and forecasting of zoonotic diseases: an overview. *Veterinary World*, *6*(11), 889.

Littman-Quinn, R., Chandra, A., Schwartz, A., Fadlelmola, F. M., Ghose, S., Luberti, A. A., Tatarsky, A., Chihanga, S., Ramogola-Masire, D., & Steenhoff, A. (2011). mHealth applications for telemedicine and public health intervention in Botswana. 2011 IST-Africa Conference Proceedings,

Madoff, L. C., & Li, A. (2014). Web-Based Surveillance Systems for Human, Animal, and Plant Diseases [Journal Article

Review]. *Microbiology spectrum*, *2*(1), OH-0015-2012. <https://doi.org/10.1128/microbiolspec.OH-0015-2012>

Manoj, S., Wijekoon, A., Dharmawardhana, M., Wijesooriya, D., Rodrigo, S., Hewapathirana, R., Siribaddana, P., Gunasekera, T., & Dissanayake, V. (2013). Implementation of District Health Information Software 2 (DHIS2) in Sri Lanka. *Sri Lanka Journal of Bio-Medical Informatics*, *Vol 3*, 109-114. <https://doi.org/10.4038/sljbmi.v3i4.5431>

Mawudeku, A., & Blench, M. (2005). Global public health intelligence network (GPHIN). Proceedings of Machine Translation Summit X: Invited papers,

Nelson R. (2008). HealthMap: the future of infectious diseases surveillance? *The Lancet Infectious Diseases*, *8*(10), 596.

Roberts, S. L., & Elbe, S. (2017). Catching the flu: Syndromic surveillance, algorithmic governmentality and global health security [Article]. *SECURITY DIALOGUE*, *48*(1), 46-62. <https://doi.org/10.1177/0967010616666443>

Roncero, A. P., Marques, G., Sainz-De-Abajo, B., Martín-Rodríguez, F., del Pozo Vegas, C., Garcia-Zapirain, B., & de la Torre-Diez, I. (2020). Mobile health apps for medical emergencies: systematic review. *JMIR mHealth and uHealth*, *8*(12), e18513.

Savelli, C. J., Abela-Ridder, B., & Miyagishima, K. (2013). Planning for rapid response to outbreaks of animal diseases transmissable to humans via food [Article]. *REVUE SCIENTIFIQUE ET TECHNIQUE-OFFICE INTERNATIONAL DES EPIZOOTIES*, *32*(2), 469-477.

Tamrat, T., & Kachnowski, S. (2012). Special delivery: an analysis of mHealth in maternal and newborn health programs and their outcomes around the world. *Maternal and child health journal*, *16*(5), 1092-1101.

Tekola, B., Myers, L., Lubroth, J., Plee, L., Calistri, P., & Pinto, J. (2017). International health threats and global early warning and response mechanisms [Article]. *REVUE SCIENTIFIQUE ET TECHNIQUE-OFFICE INTERNATIONAL DES EPIZOOTIES*, *36*(2), 657-670. <https://doi.org/10.20506/rst.36.2.2683>

Utunen, H., Tokar, A., Dancante, M., & Piroux, C. (2023). Online learning for WHO priority diseases with pandemic potential: evidence from existing courses and preparing for Disease X. *Archives of Public Health*, *81*(1), 61. <https://doi.org/10.1186/s13690-023-01080-9>

Vandersmissen, A., & Welburn, S. C. (2014). Current initiatives in One Health: consolidating the One Health Global Network [Article]. *REVUE SCIENTIFIQUE ET TECHNIQUE-OFFICE INTERNATIONAL DES EPIZOOTIES*, *33*(2), 421-432. <https://doi.org/10.20506/rst.33.2.2297>

Vardell, E. (2020). Global health observatory data repository. *Medical reference services quarterly*, *39*(1), 67-74.

Wang, Y., Li, B., & Liu, L. (2020). Telemedicine experience in China: our response to the pandemic and current challenges. *Frontiers in Public Health*, *8*, 549669.

Yu, V. L., & Madoff, L. C. (2004). ProMED-mail: An Early Warning System for Emerging Diseases. *Clinical Infectious Diseases*, *39*(2), 227-232. <https://doi.org/10.1086/422003>
