# Supplementary material for: Knowledge attributes of public health management information systems used in health emergencies: a scoping review
Source: Front Public Health. 2025 Mar 20;12:1458867. doi: 10.3389/fpubh.2024.1458867 (PMC11969037; doi:10.3389/fpubh.2024.1458867)
Supplement: SUPPLEMENTARY DATA SHEET 3 — Supplementary Tables C1 to C9. [file Data_Sheet_3.zip › SupplementaryTables_C1_C9_KnowledgeAttributesPerHMIS/SupplementaryTable_C4_Applicable.docx]

**Supplementary table C4: Literary sources for knowledge attributes of HMIS reviewed in the study – Applicability.**

|  | **IMS** | **Universal/Global** | **Local** |
| --- | --- | --- | --- |
|  | TACIT Knowledge containing IMS | | |
|  | GPHIN | (Blench, 2007; Carter et al., 2020; Keller et al., 2009; Mawudeku & Blench, 2005; Roberts & Elbe, 2017; Young et al., 2015) |  |
|  | GLEWS | (Thakur, 2022; World Organisation for Animal Health, nd.) |  |
|  | HealthMap | (Ahmed et al., 2015; Brownstein et al., 2008; Carita A, 2014; Freifeld et al., 2008; Nelson R, 2008) |  |
|  | OpenWHO | (Bonkoungou et al., 2023; George et al., 2022; Goldin et al., 2021; Rohloff et al., 2018; Utunen, 2021; Utunen et al., 2023; Utunen et al., 2022; Utunen et al., 2020; Utunen et al., 2021) |  |
|  | ProMED | (Al-Tawfiq et al., 2014; Carrion & Madoff, 2017; Cowen et al., 2006; Hugh-Jones, 2001; You et al., 2021) |  |
|  | Telemedicine |  | (Bashshur, 2001; Bashshur et al., 2002; Doraiswamy et al., 2020; Kilova & Uzunova, 2020; Pierce & Stevermer, 2020; Shanbehzadeh et al., 2021; Song et al., 2020) |
|  | mHealth | (Abaza & Marschollek, 2017; Albabtain et al., 2014; Kahn et al., 2010; Littman-Quinn et al., 2011; Sanjeev D & Anuradha D, 2014; Shahriar A & Pradeep R, 2010; Steinhubl et al., 2013; Tamrat & Kachnowski, 2012; Varshney, 2014) |  |
|  | EXPLICIT Knowledge containing IMS | | |
|  | COVID-19 | (Ahmed et al., 2020; Allan et al., 2022; Irwansyah et al., 2020) |  |
|  | EOC |  | (Callan, 2020; Ma et al., 2020; Su et al., 2017; World Health Organization, 2014) |
|  | HDX |  | (Kelly, 2020; Paulus et al., 2018) |
|  | DHIS |  | (DHIS2, nd; Farnham et al., 2020) |
|  | GIS | (Fuad et al., 2006; Kaiser et al., 2003; Tanser & Le Sueur, 2002) |  |
|  | GHO | (Ampofo & Boateng, 2020; Anderson et al., 2016; Cui et al., 2021; Huang et al., 2024; Huang et al., 2022; Huang et al., 2021; Jiang et al., 2023; Li et al., 2023; Liu et al., 2017; Lu et al., 2021; Nazir et al., 2019; Rodi et al., 2022; Shah et al., 2017; Swarnamali et al., 2022; Tidman et al., 2023; Wu & Reynolds, 2023; Zaveri et al., 2013) |  |

Abaza, H., & Marschollek, M. (2017). mHealth application areas and technology combinations. *Methods of information in medicine*, *56*(S 01), e105-e122.

Ahmed, K., Bukhari, M. A., Mlanda, T., Kimenyi, J. P., Wallace, P., Lukoya, C. O., Hamblion, E. L., & Impouma, B. (2020). Novel approach to support rapid data collection, management, and visualization during the COVID-19 outbreak response in the world health organization African region: development of a data summarization and visualization tool. *JMIR Public Health and Surveillance*, *6*(4), e20355.

Ahmed, S. S., Oviedo-Orta, E., Mekaru, S. R., Freifeld, C. C., Tougas, G., & Brownstein, J. S. (2015). Surveillance for <i>Neisseria meningitidis</i> Disease Activity and Transmission Using Information Technology [Article]. *PLOS ONE*, *10*(5), Article e0127406. <https://doi.org/10.1371/journal.pone.0127406>

Al-Tawfiq, J. A., Zumla, A., Gautret, P., Gray, G. C., Hui, D. S., Al-Rabeeah, A. A., & Memish, Z. A. (2014). Emerging respiratory tract infections 1 Surveillance for emerging respiratory viruses [Article]. *LANCET INFECTIOUS DISEASES*, *14*(10), 992-1000. <https://doi.org/10.1016/S1473-3099(14)70840-0>

Albabtain, A. F., AlMulhim, D. A., Yunus, F., & Househ, M. S. (2014). The role of mobile health in the developing world: a review of current knowledge and future trends. *Journal of Selected Areas in Health Informatics*, *4*(2), 10-15.

Allan, M., Lièvre, M., Laurenson-Schafer, H., de Barros, S., Jinnai, Y., Andrews, S., Stricker, T., Formigo, J. P., Schultz, C., & Perrocheau, A. (2022). The World Health Organization COVID-19 surveillance database. *International journal for equity in health*, *21*(Suppl 3), 167.

Ampofo, A. G., & Boateng, E. B. (2020). Beyond 2020: Modelling obesity and diabetes prevalence [Article]. *DIABETES RESEARCH AND CLINICAL PRACTICE*, *167*, Article 108362. <https://doi.org/10.1016/j.diabres.2020.108362>

Anderson, C. L., Becher, H., & Winkler, V. (2016). Tobacco Control Progress in Low and Middle Income Countries in Comparison to High Income Countries [Article]. *INTERNATIONAL JOURNAL OF ENVIRONMENTAL RESEARCH AND PUBLIC HEALTH*, *13*(10), Article 1039. <https://doi.org/10.3390/ijerph13101039>

Bashshur, R. L. (2001). Where we are in telemedicine/telehealth, and where we go from here [Editorial Material]. *TELEMEDICINE JOURNAL AND E-HEALTH*, *7*(4), 273-277. <https://doi.org/10.1089/15305620152814665>

Bashshur, R. L., Mandil, S. H., & Shannon, G. W. (2002). Executive summary [Editorial Material]. *TELEMEDICINE JOURNAL AND E-HEALTH*, *8*(1), 95-107. <https://doi.org/10.1089/15305620252933437>

Blench, M. (2007). Global public health intelligence network (GPHIN). Proceedings of Machine Translation Summit XI: Papers,

Bonkoungou, B., Utunen, H., Talisuna, A. O., O'Connell, G., Koua, E., Chamla, D. D., Arabi, E., Tokar, A., & Gueye, A. S. (2023). Online capacity building for the health workforce: the case of the Integrated Disease Surveillance and Response for the African region [Article]. *JOURNAL OF PUBLIC HEALTH IN AFRICA*, *14*(12), Article 2478. <https://doi.org/10.4081/jphia.2023.2478>

Brownstein, J. S., Freifeld, C. C., Reis, B. Y., & Mandl, K. D. (2008). Surveillance Sans Frontières: Internet-Based Emerging Infectious Disease Intelligence and the HealthMap Project. *PLOS Medicine*, *5*(7), e151. <https://doi.org/10.1371/journal.pmed.0050151>

Callan, T. (2020). Emergency operations centres: models and core principles [Article]. *REVUE SCIENTIFIQUE ET TECHNIQUE-OFFICE INTERNATIONAL DES EPIZOOTIES*, *39*(2), 399-405. <https://doi.org/10.20506/rst.39.2.3091>

Carita A. (2014). Healthmap. In *Reference Reviews* (Vol. 28, pp. 30-31). Emerald Group Publishing Limited. <https://doi.org/10.1108/RR-06-2013-0162>

Carrion, M., & Madoff, L. C. (2017). ProMED-mail: 22 years of digital surveillance of emerging infectious diseases. *International Health*, *9*(3), 177-183. <https://doi.org/10.1093/inthealth/ihx014>

Carter, D., Stojanovic, M., Hachey, P., Fournier, K., Rodier, S., Wang, Y., & de Bruijn, B. (2020, 2020). *Global Public Health Surveillance Using Media Reports: Redesigning GPHIN* [Proceedings Paper]. DIGITAL PERSONALIZED HEALTH AND MEDICINE,

Cowen, P., Garland, T., Hugh-Jones, M. E., Shimshony, A., Handysides, S., Kaye, D., Madoff, L. C., Pollack, M. P., & Woodall, J. (2006). Evaluation of ProMED-mail as an electronic early warning system for emerging animal diseases: 1996 to 2004. *Journal of the American Veterinary Medical Association*, *229*(7), 1090-1099.

Cui, C. L., Dornisch, A. M., Umlauf, A. E., Cuomo, R. E., Murphy, J. D., & Lopez, N. E. (2021). International Socioeconomic Predictors of Colon and Rectal Cancer Mortality: Is Colorectal Cancer a First World Problem? [Article]. *JCO GLOBAL ONCOLOGY*, *7*, 1659-1667. <https://doi.org/10.1200/GO.21.00307>

DHIS2. (nd). About DHIS. <https://dhis2.org/about/>

Doraiswamy, S., Abraham, A., Mamtani, R., & Cheema, S. (2020). *Use of telemedicine/ telehealth for geriatric care during the COVID-19 pandemic - A scoping review and evidence mapping*. <https://doi.org/10.17605/OSF.IO/26Z74>

Farnham, A., Utzinger, J., Kulinkina, A. V., & Winkler, M. S. (2020). Using district health information to monitor sustainable development. *Bull World Health Organ*, *98*(1), 69-71. <https://doi.org/10.2471/blt.19.239970>

Freifeld, C. C., Mandl, K. D., Reis, B. Y., & Brownstein, J. S. (2008). HealthMap: global infectious disease monitoring through automated classification and visualization of Internet media reports. *Journal of the American Medical Informatics Association*, *15*(2), 150-157.

Fuad, A., Kusnanto, H., Utarini, A., Dijk, J. V., & Groothoff, J. (2006). The use of geographic information systems (GIS) for rapid assessment of health facilities following a disaster: the case of the tsunami disaster in the province of Aceh. *APAMI 2006*.

George, R., Utunen, H., Ndiaye, N., Tokar, A., Mattar, L., Piroux, C., & Gamhewage, G. (2022). Ensuring equity in access to online courses: Perspectives from the WHO health emergency learning response. *World Medical & Health Policy*, *14*(2), 413-427.

Goldin, S., Kong, S. Y. J., Tokar, A., Utunen, H., Ndiaye, N., Bahl, J., Appuhamy, R., & Moen, A. (2021). Learning From a Massive Open Online COVID-19 Vaccination Training Experience: Survey Study [Article]. *JMIR PUBLIC HEALTH AND SURVEILLANCE*, *7*(12), Article e33455. <https://doi.org/10.2196/33455>

Huang, J., Chan, S. C., Pang, W. S., Liu, X., Zhang, L., Lucero-Prisno Iii, D. E., Xu, W., Zheng, Z.-J., Ng, A. C.-F., Necchi, A., Spiess, P. E., Teoh, J. Y.-C., Wong, M. C. S., & Global Soc Rare Genitourinary, T. (2024). Incidence, risk factors, and temporal trends of penile cancer: a global population-based study [Article]. *BJU INTERNATIONAL*, *133*(3), 314-323. <https://doi.org/10.1111/bju.16224>

Huang, J., Leung, D. K.-W., Chan, E. O.-T., Lok, V., Leung, S., Wong, I., Lao, X.-Q., Zheng, Z.-J., Chiu, P. K.-F., Ng, C.-F., Wong, J. H.-M., Volpe, A., Merseburger, A. S., Powles, T., Teoh, J. Y.-C., & Wong, M. C. S. (2022). A Global Trend Analysis of Kidney Cancer Incidence and Mortality and Their Associations with Smoking, Alcohol Consumption, and Metabolic Syndrome [Article]. *EUROPEAN UROLOGY FOCUS*, *8*(1), 200-209. <https://doi.org/10.1016/j.euf.2020.12.020>

Huang, J., Lok, V., Ngai, C. H., Zhang, L., Yuan, J., Lao, X. Q., Ng, K., Chong, C., Zheng, Z.-J., & Wong, M. C. S. (2021). Worldwide Burden of, Risk Factors for, and Trends in Pancreatic Cancer [Article]. *GASTROENTEROLOGY*, *160*(3), 744-754. <https://doi.org/10.1053/j.gastro.2020.10.007>

Hugh-Jones, M. (2001). Global awareness of disease outbreaks: the experience of ProMED-mail. *Public Health Reports*, *116*(Suppl 2), 27.

Irwansyah, E., Budiharto, W., Widhyatmoko, D., Istamar, A., & Panghurian, F. P. (2020). Monitoring Coronavirus COVID-19/SARS-CoV-2 Pandemic using GIS Dashboard: International and Indonesia Context. *Preprints* <https://doi.org/10.20944/preprints202008.0415.v1> I

Jiang, B., Wu, T., Liu, W., Liu, G., & Lu, P. (2023). Changing Trends in the Global Burden of Cataract Over the Past 30 Years: Retrospective Data Analysis of the Global Burden of Disease Study 2019 [Article]. *JMIR PUBLIC HEALTH AND SURVEILLANCE*, *9*, Article e47349. <https://doi.org/10.2196/47349>

Kahn, J. G., Yang, J. S., & Kahn, J. S. (2010). ‘Mobile’health needs and opportunities in developing countries. *Health affairs*, *29*(2), 252-258.

Kaiser, R., Spiegel, P. B., Henderson, A. K., & Gerber, M. L. (2003). The application of geographic information systems and global positioning systems in humanitarian emergencies: lessons learned, programme implications and future research. *Disasters*, *27*(2), 127-140.

Keller, M., Blench, M., Tolentino, H., Freifeld, C. C., Mandl, K. D., Mawudeku, A., Eysenbach, G., & Brownstein, J. S. (2009). Use of Unstructured Event-Based Reports for Global Infectious Disease Surveillance [Article]. *EMERGING INFECTIOUS DISEASES*, *15*(5), 689-695. <https://doi.org/10.3201/eid1505.081114>

Kelly, L. (2020). Humanitarian evidence summary No. 7.

Kilova, K., & Uzunova, S. (2020). Telemedicine in assistance to healthcare in the COVID-19 pandemic. *Acta Medica Bulgarica*, *47*(4), 63-68.

Li, Z., Cai, Z., & Yip, P. S. F. (2023). One stream, two channels? A parallel-process latent class growth model of homicide rates and suicide rates in 183 countries, between 2000 and 2019 [Article]. *SSM-POPULATION HEALTH*, *22*, Article 101376. <https://doi.org/10.1016/j.ssmph.2023.101376>

Littman-Quinn, R., Chandra, A., Schwartz, A., Fadlelmola, F. M., Ghose, S., Luberti, A. A., Tatarsky, A., Chihanga, S., Ramogola-Masire, D., & Steenhoff, A. (2011). mHealth applications for telemedicine and public health intervention in Botswana. 2011 IST-Africa Conference Proceedings,

Liu, J. X., Goryakin, Y., Maeda, A., Bruckner, T., & Scheffler, R. (2017). Global Health Workforce Labor Market Projections for 2030 [Article]. *HUMAN RESOURCES FOR HEALTH*, *15*, Article 11. <https://doi.org/10.1186/s12960-017-0187-2>

Lu, B., Li, N., Luo, C.-Y., Cai, J., Lu, M., Zhang, Y.-H., Chen, H.-D., & Dai, M. (2021). Colorectal cancer incidence and mortality: the current status, temporal trends and their attributable risk factors in 60 countries in 2000-2019 [Article]. *CHINESE MEDICAL JOURNAL*, *134*(16), 1941-1951. <https://doi.org/10.1097/CM9.0000000000001619>

Ma, J., Huang, Y., & Zheng, Z.-J. (2020). Leveraging the Public Health Emergency Operation Center (PHEOC) for pandemic response: opportunities and challenges [Journal Article

Review]. *Global health journal (Amsterdam, Netherlands)*, *4*(4), 118-120. <https://doi.org/10.1016/j.glohj.2020.11.004>

Mawudeku, A., & Blench, M. (2005). Global public health intelligence network (GPHIN). Proceedings of Machine Translation Summit X: Invited papers,

Nazir, M. A., Al-Ansari, A., Abbasi, N., & Almas, K. (2019). Global Prevalence of Tobacco Use in Adolescents and Its Adverse Oral Health Consequences [Journal Article]. *Open access Macedonian journal of medical sciences*, *7*(21), 3659-3666. <https://doi.org/10.3889/oamjms.2019.542>

Nelson R. (2008). HealthMap: the future of infectious diseases surveillance? *The Lancet Infectious Diseases*, *8*(10), 596.

Paulus, D., Meesters, K., & Van de Walle, B. A. (2018). Turning data into action: supporting humanitarian field workers with open data. Iscram,

Pierce, R. P., & Stevermer, J. J. (2020). Disparities in the use of telehealth at the onset of the COVID-19 public health emergency. *Journal of Telemedicine and Telecare*, *29*(1), 3-9. <https://doi.org/10.1177/1357633X20963893>

Roberts, S. L., & Elbe, S. (2017). Catching the flu: Syndromic surveillance, algorithmic governmentality and global health security [Article]. *SECURITY DIALOGUE*, *48*(1), 46-62. <https://doi.org/10.1177/0967010616666443>

Rodi, P., Obermeyer, W., Pablos-Mendez, A., Gori, A., & Raviglione, M. C. (2022). *Overview of DAH, number of deaths, mortality rates, and health expenditures*. <https://doi.org/10.1371/journal.pmed.1003873.g001>

Rohloff, T., Utunen, H., Renz, J., Zhao, Y., Gamhewage, G., & Meinel, C. (2018). OpenWHO: Integrating Online Knowledge Transfer into Health Emergency Response. EC-TEL (Practitioner Proceedings),

Sanjeev D, & Anuradha D. (2014). Mobile-health technology: Can it Strengthen and improve public health systems of other developing countries as per Indian strategies? A systematic review of the literature. *International Journal of medicine and public Health*, *4*(1).

Shah, N. D., Cruz-Lemini, M., Stein, E., Abraldes, J., Altamirano, J., & Bataller, R. (2017). COLDER WEATHER AND FEWER SUNLIGHT HOURS INCREASE THE WEIGHT OF ALCOHOL AS A CAUSE OF CIRRHOSIS WORLDWIDE [Meeting Abstract]. *GASTROENTEROLOGY*, *152*(5), S942-S943. <https://doi.org/10.1016/S0016-5085(17)33209-2>

Shahriar A, & Pradeep R. (2010). mHealth-an ultimate platform to serve the unserved. *Yearbook of medical informatics*, *19*(01), 94-100.

Shanbehzadeh, M., Kazemi-Arpanahi, H., Kalkhajeh, S. G., & Basati, G. (2021). Systematic review on telemedicine platforms in lockdown periods: Lessons learned from the COVID-19 pandemic. *Journal of Education and Health Promotion*, *10*.

Song, X., Liu, X., & Wang, C. (2020). The role of telemedicine during the COVID-19 epidemic in China—experience from Shandong province. *Critical Care*, *24*(1), 178. <https://doi.org/10.1186/s13054-020-02884-9>

Steinhubl, S. R., Muse, E. D., & Topol, E. J. (2013). Can mobile health technologies transform health care? *Jama*, *310*(22), 2395-2396.

Su, Y.-F., Wu, C.-H., & Lee, T.-F. (2017). PUBLIC HEALTH EMERGENCY RESPONSE IN TAIWAN [Article]. *HEALTH SECURITY*, *15*(2), 137-143. <https://doi.org/10.1089/hs.2016.0108>

Swarnamali, H., Jayawardena, R., Chourdakis, M., & Ranasinghe, P. (2022). Is the proportion of per capita fat supply associated with the prevalence of overweight and obesity? an ecological analysis [Article]. *BMC NUTRITION*, *8*(1), Article 4. <https://doi.org/10.1186/s40795-021-00496-2>

Tamrat, T., & Kachnowski, S. (2012). Special delivery: an analysis of mHealth in maternal and newborn health programs and their outcomes around the world. *Maternal and child health journal*, *16*(5), 1092-1101.

Tanser, F. C., & Le Sueur, D. (2002). The application of geographical information systems to important public health problems in Africa. *International journal of health geographics*, *1*, 1-9.

Thakur, S. D. (2022). Early Warning Systems, Disease Management, and Biosecurity in Disasters. In *Management of Animals in Disasters* (pp. 25-37). Springer.

Tidman, R., Kanankege, K. S. T., Bangert, M., & Abela-Ridder, B. (2023). Global prevalence of 4 neglected foodborne trematodes targeted for control by WHO: A scoping review to highlight the gaps [Review]. *PLOS NEGLECTED TROPICAL DISEASES*, *17*(3), Article e0011073. <https://doi.org/10.1371/journal.pntd.0011073>

Utunen, H. (2021). Transferring real-time knowledge free of charge through WHO’s online learning platform OpenWHO. org. *QScience Proceedings*, *2022*(1), 5.

Utunen, H., Appuhamy, R., Attias, M., Ndiaye, N., George, R., Arabi, E., & Tokar, A. (2023). Observations from three years of online pandemic learning response on OpenWHO. *The International Journal of Information and Learning Technology*, *40*(5), 527-540.

Utunen, H., Ndiaye, N., Attias, M., Mattar, L., Tokar, A., & Gamhewage, G. (2022). Multilingual Approach to COVID-19 Online Learning Response on OpenWHO. org. *Informatics and Technology in Clinical Care and Public Health*, *289*, 192.

Utunen, H., Ndiaye, N., Piroux, C., George, R., Attias, M., & Gamhewage, G. (2020). Global reach of an online COVID-19 course in multiple languages on OpenWHO in the first quarter of 2020: analysis of platform use data. *Journal of medical Internet research*, *22*(4), e19076.

Utunen, H., Van Kerkhove, M. D., Tokar, A., O'Connell, G., Gamhewage, G. M., & Fall, I. S. (2021). One year of pandemic learning response: benefits of massive online delivery of the World Health Organization’s technical guidance. *JMIR Public Health and Surveillance*, *7*(4), e28945.

Varshney, U. (2014). Mobile health: Four emerging themes of research. *Decision Support Systems*, *66*, 20-35.

World Health Organization. (2014). A systematic review of public health emergency operations centres (EOC): December 2013. <https://www.who.int/publications/i/item/WHO-HSE-GCR-2014.1>

World Organisation for Animal Health. (nd.). About GLEWS and GLEWS+. <http://www.glews.net/?page_id=1059>

Wu, T. J., & Reynolds, M. M. (2023). Trachoma, the world's leading infectious cause of blindness: The remaining gap in care and access to basic handwashing facilities [Article]. *EUROPEAN JOURNAL OF OPHTHALMOLOGY*, *33*(4), 1576-1582. <https://doi.org/10.1177/11206721231154295>

You, J., Expert, P., & Costelloe, C. (2021). Using text mining to track outbreak trends in global surveillance of emerging diseases: ProMED-mail. *Journal of the Royal Statistical Society Series A: Statistics in Society*, *184*(4), 1245-1259.

Young, M. M., Dubeau, C., & Corazza, O. (2015). Detecting a signal in the noise: monitoring the global spread of novel psychoactive substances using media and other open-source information [Article]. *HUMAN PSYCHOPHARMACOLOGY-CLINICAL AND EXPERIMENTAL*, *30*(4), 319-326. <https://doi.org/10.1002/hup.2477>

Zaveri, A., Lehmann, J., Auer, S., Hassan, M. M., Sherif, M. A., & Martin, M. (2013). Publishing and interlinking the global health observatory dataset. *Semantic Web*, *4*(3), 315-322.
