# Supplementary material for: Knowledge attributes of public health management information systems used in health emergencies: a scoping review
Source: Front Public Health. 2025 Mar 20;12:1458867. doi: 10.3389/fpubh.2024.1458867 (PMC11969037; doi:10.3389/fpubh.2024.1458867)
Supplement: SUPPLEMENTARY DATA SHEET 3 — Supplementary Tables C1 to C9. [file Data_Sheet_3.zip › SupplementaryTables_C1_C9_KnowledgeAttributesPerHMIS/SupplementaryTable_C5_Accessible.docx]

**Supplementary table C5: Literary sources for knowledge attributes of HMIS reviewed in the study -Accessibility.**

|  | **IMS** | **Public/Open source** | **Private / Close source** |
| --- | --- | --- | --- |
|  | TACIT Knowledge containing IMS | | |
|  | GPHIN |  | (Madoff & Li, 2014; Mykhalovskiy & Weir, 2006) |
|  | GLEWS | (Marvin et al., 2009; World Organisation for Animal Health, 2023) |  |
|  | HealthMap | (Ahmed et al., 2015; Brownstein & Freifeld, 2007; Chen et al., 2010; Freifeld et al., 2008; Nelson R, 2008) |  |
|  | OpenWHO | (George et al., 2022; Rohloff et al., 2018; Utunen, 2021; Utunen et al., 2022; Utunen, Staubitz, et al., 2023; Utunen, Tokar, et al., 2023; Utunen et al., 2021) |  |
|  | ProMED | (Carrion & Madoff, 2017; Madoff & Woodall, 2005; You et al., 2021) |  |
|  | Telemedicine |  | (Bashshur et al., 2002; Greiwe, 2022; Song et al., 2020) |
|  | mHealth |  | (Mohanty et al., 2019; Roncero et al., 2020; Vahidi et al., 2021; Varshney, 2014) |
|  | EXPLICIT Knowledge containing IMS | | |
|  | COVID-19 | (World Health Organization, 2023,) |  |
|  | EOC |  | (Chipman & Wuerfel, 2008) |
|  | HDX | (Telford, 2020; Weaver et al., 2019) |  |
|  | DHIS |  | (DHIS2, nd; Joseph et al., 2022; Manya et al., 2012; Thangasamy et al., 2016) |
|  | GIS |  | (Tanser & Le Sueur, 2002; Tomaszewski et al., 2015) (Melnick, 2002) |
|  | GHO | (Onagbiye et al., 2023; Vardell, 2020) |  |

Ahmed, S. S., Oviedo-Orta, E., Mekaru, S. R., Freifeld, C. C., Tougas, G., & Brownstein, J. S. (2015). Surveillance for <i>Neisseria meningitidis</i> Disease Activity and Transmission Using Information Technology [Article]. *PLOS ONE*, *10*(5), Article e0127406. <https://doi.org/10.1371/journal.pone.0127406>

Bashshur, R. L., Mandil, S. H., & Shannon, G. W. (2002). Executive summary [Editorial Material]. *TELEMEDICINE JOURNAL AND E-HEALTH*, *8*(1), 95-107. <https://doi.org/10.1089/15305620252933437>

Brownstein, J. S., & Freifeld, C. (2007). HealthMap: the development of automated real-time internet surveillance for epidemic intelligence. *Weekly releases (1997–2007)*, *12*(48), 3322.

Carrion, M., & Madoff, L. C. (2017). ProMED-mail: 22 years of digital surveillance of emerging infectious diseases. *International Health*, *9*(3), 177-183. <https://doi.org/10.1093/inthealth/ihx014>

Chen, H., Zeng, D., Yan, P., Chen, H., Zeng, D., & Yan, P. (2010). HealthMap. *Infectious Disease Informatics: Syndromic Surveillance for Public Health and BioDefense*, 183-186.

Chipman, R., & Wuerfel, R. (2008). Network based information sharing between emergency operations center. 2008 IEEE Conference on Technologies for Homeland Security,

DHIS2. (nd). About DHIS. <https://dhis2.org/about/>

Freifeld, C. C., Mandl, K. D., Reis, B. Y., & Brownstein, J. S. (2008). HealthMap: global infectious disease monitoring through automated classification and visualization of Internet media reports. *Journal of the American Medical Informatics Association*, *15*(2), 150-157.

George, R., Utunen, H., Ndiaye, N., Tokar, A., Mattar, L., Piroux, C., & Gamhewage, G. (2022). Ensuring equity in access to online courses: Perspectives from the WHO health emergency learning response. *World Medical & Health Policy*, *14*(2), 413-427.

Greiwe, J. (2022). Telemedicine Lessons Learned During the COVID-19 Pandemic. *Current Allergy and Asthma Reports*, *22*(1), 1-5. <https://doi.org/10.1007/s11882-022-01026-1>

Joseph, J. J., Mkali, H. R., Reaves, E. J., Mwaipape, O. S., Mohamed, A., Lazaro, S. N., Aaron, S., Chacky, F., Mahendeka, A., Rulagirwa, H. S., Mwenesi, M., Mwakapeje, E., Ally, A. Y., Kitojo, C., Serbantez, N., Nyinondi, S., Lalji, S. M., Wilillo, R., Al-mafazy, A.-w., . . . Ngondi, J. M. (2022). Improvements in malaria surveillance through the electronic Integrated Disease Surveillance and Response (eIDSR) system in mainland Tanzania, 2013–2021. *Malaria Journal*, *21*(1), 321. <https://doi.org/10.1186/s12936-022-04353-w>

Madoff, L. C., & Li, A. (2014). Web-Based Surveillance Systems for Human, Animal, and Plant Diseases [Journal Article

Review]. *Microbiology spectrum*, *2*(1), OH-0015-2012. <https://doi.org/10.1128/microbiolspec.OH-0015-2012>

Madoff, L. C., & Woodall, J. P. (2005). The Internet and the Global Monitoring of Emerging Diseases: Lessons from the First 10 Years of ProMED-mail. *Archives of Medical Research*, *36*(6), 724-730. <https://doi.org/https://doi.org/10.1016/j.arcmed.2005.06.005>

Manya, A., Braa, J., Øverland, L., Titlestad, O., Mumo, J., & Nzioka, C. (2012). *National Roll out of District Health Information Software (DHIS 2) in Kenya, 2011 – Central Server and Cloud based Infrastructure*.

Marvin, H., Kleter, G., Prandini, A., Dekkers, S., & Bolton, D. (2009). Early identification systems for emerging foodborne hazards. *Food and Chemical Toxicology*, *47*(5), 915-926.

Melnick, A. L. (2002). *Introduction to geographic information systems in public health*. Jones & Bartlett Learning.

Mohanty, B., Chughtai, A., & Rabhi, F. (2019). Use of Mobile Apps for epidemic surveillance and response–availability and gaps. *Global Biosecurity*, *1*(1).

Mykhalovskiy, E., & Weir, L. (2006). The Global Public Health Intelligence Network and early warning outbreak detection: a Canadian contribution to global public health. *Canadian journal of public health*, *97*, 42-44.

Nelson R. (2008). HealthMap: the future of infectious diseases surveillance? *The Lancet Infectious Diseases*, *8*(10), 596.

Onagbiye, S., Ricci, H., Bester, P., & Ricci, C. (2023). Sedentariness and overweight in relation to mortality in sub-Saharan Africa. A mediation analysis based on the World Health Organization-Global Health Observatory data repository [Article]. *JOURNAL OF PUBLIC HEALTH IN AFRICA*, *14*(4), Article 2155. <https://doi.org/10.4081/jphia.2023.2155>

Rohloff, T., Utunen, H., Renz, J., Zhao, Y., Gamhewage, G., & Meinel, C. (2018). OpenWHO: Integrating Online Knowledge Transfer into Health Emergency Response. EC-TEL (Practitioner Proceedings),

Roncero, A. P., Marques, G., Sainz-De-Abajo, B., Martín-Rodríguez, F., del Pozo Vegas, C., Garcia-Zapirain, B., & de la Torre-Diez, I. (2020). Mobile health apps for medical emergencies: systematic review. *JMIR mHealth and uHealth*, *8*(12), e18513.

Song, X., Liu, X., & Wang, C. (2020). The role of telemedicine during the COVID-19 epidemic in China—experience from Shandong province. *Critical Care*, *24*(1), 178. <https://doi.org/10.1186/s13054-020-02884-9>

Tanser, F. C., & Le Sueur, D. (2002). The application of geographical information systems to important public health problems in Africa. *International journal of health geographics*, *1*, 1-9.

Telford, S. (2020). Case Study-The Humanitarian Data Exchange: Critical Decisions, Key Results and The Road Ahead. <https://centre.humdata.org/wp-content/uploads/2020/09/hdxcasestudy.pdf>

Thangasamy, P., Gebremichael, M., Kebede, M., Sileshi, M., Elias, N., & Tesfaye, B. (2016). A pilot study on district health information software 2: challenges and lessons learned in a developing country: an experience from Ethiopia. *Int Res J Eng Technol*, *3*(5), 1646-1651.

Tomaszewski, B., Judex, M., Szarzynski, J., Radestock, C., & Wirkus, L. (2015). Geographic information systems for disaster response: A review. *Journal of Homeland Security and Emergency Management*, *12*(3), 571-602.

Utunen, H. (2021). Transferring real-time knowledge free of charge through WHO’s online learning platform OpenWHO. org. *QScience Proceedings*, *2022*(1), 5.

Utunen, H., Ndiaye, N., Attias, M., Mattar, L., Tokar, A., & Gamhewage, G. (2022). Multilingual Approach to COVID-19 Online Learning Response on OpenWHO. org. *Informatics and Technology in Clinical Care and Public Health*, *289*, 192.

Utunen, H., Staubitz, T., George, R., Zhao, Y. U., Serth, S., & Tokar, A. (2023). Scale Up Multilingualism in Health Emergency Learning: Developing an Automated Transcription and Translation Tool. In *Caring is Sharing–Exploiting the Value in Data for Health and Innovation* (pp. 408-412). IOS Press.

Utunen, H., Tokar, A., Dancante, M., & Piroux, C. (2023). Online learning for WHO priority diseases with pandemic potential: evidence from existing courses and preparing for Disease X. *Archives of Public Health*, *81*(1), 61. <https://doi.org/10.1186/s13690-023-01080-9>

Utunen, H., Van Kerkhove, M. D., Tokar, A., O'Connell, G., Gamhewage, G. M., & Fall, I. S. (2021). One year of pandemic learning response: benefits of massive online delivery of the World Health Organization’s technical guidance. *JMIR Public Health and Surveillance*, *7*(4), e28945.

Vahidi, H., Taleai, M., Yan, W., & Shaw, R. (2021). Digital Citizen Science for Responding to COVID-19 Crisis: Experiences from Iran [Article]. *INTERNATIONAL JOURNAL OF ENVIRONMENTAL RESEARCH AND PUBLIC HEALTH*, *18*(18), Article 9666. <https://doi.org/10.3390/ijerph18189666>

Vardell, E. (2020). Global health observatory data repository. *Medical reference services quarterly*, *39*(1), 67-74.

Varshney, U. (2014). Mobile health: Four emerging themes of research. *Decision Support Systems*, *66*, 20-35.

Weaver, C., Powell, J., & Leson, H. (2019). Development assistance and humanitarian action. In *The State of Open Data* (pp. 77).

World Health Organization. (2023,). WHO COVID-19 dashboard. <https://data.who.int/dashboards/covid19/about?n=c>

World Organisation for Animal Health. (2023). WAHIS: World Animal Health Information System. <https://wahis.woah.org/#/home>

You, J., Expert, P., & Costelloe, C. (2021). Using text mining to track outbreak trends in global surveillance of emerging diseases: ProMED-mail. *Journal of the Royal Statistical Society Series A: Statistics in Society*, *184*(4), 1245-1259.
