# Supplementary material for: Knowledge attributes of public health management information systems used in health emergencies: a scoping review
Source: Front Public Health. 2025 Mar 20;12:1458867. doi: 10.3389/fpubh.2024.1458867 (PMC11969037; doi:10.3389/fpubh.2024.1458867)
Supplement: SUPPLEMENTARY DATA SHEET 3 — Supplementary Tables C1 to C9. [file Data_Sheet_3.zip › SupplementaryTables_C1_C9_KnowledgeAttributesPerHMIS/SupplementaryTable_C6_Programm.docx]

**Supplementary table C6: Literary sources for knowledge attributes of HMIS reviewed in the study – Programmability.**

|  | **IMS** | **High** | **Moderate** |
| --- | --- | --- | --- |
|  | TACIT Knowledge containing IMS | | |
|  | GPHIN | (Blench, 2007; Carter et al., 2020; Mawudeku et al., 2013) |  |
|  | GLEWS | (Al-Hemoud et al., 2021; World Organisation for Animal Health, 2023) |  |
|  | HealthMap | (Ahmed et al., 2015; Carita A, 2014; Freifeld et al., 2008; Lyon et al., 2012) |  |
|  | OpenWHO | (Bonkoungou et al., 2023; George et al., 2022; Goldin et al., 2021; Rohloff et al., 2018; Utunen, 2021; Utunen, Attias, et al., 2022; Utunen, Ndiaye, et al., 2022; Utunen et al., 2023) |  |
|  | ProMED | (Hugh-Jones, 2001; Woodall, 2001; Yu & Madoff, 2004) |  |
|  | Telemed | (Bashshur et al., 2002; Litvak et al., 2022; Ye, 2020) |  |
|  | mHealth |  | (Albabtain et al., 2014; Burner et al., 2019) |
|  | EXPLICIT Knowledge containing IMS | | |
|  | COVID-19 | (Ahmed et al., 2020; Allan et al., 2022; Sulaiman et al., 2020) |  |
|  | EOC | (Davis, 2002; Ma et al., 2020) | (Davis, 2002; Ma et al., 2020) |
|  | HDX |  | (Abuoda et al., 2021; Paulus et al., 2018) |
|  | DHIS | (Asaduzzaman et al., 2024; Eggers et al., 2022; Kinkade et al., 2022) |  |
|  | GIS |  | (Gülden et al., 2004; Kaiser et al., 2003; Tanser & Le Sueur, 2002) |
|  | GHO |  | (Vardell, 2020; Zaveri et al., 2013) |

Abuoda, G., Hendrix, C., & Campo, S. (2021). Automatic Tag Recommendation for the UN Humanitarian Data Exchange. BIRDS+ WEPIR@ CHIIR,

Ahmed, K., Bukhari, M. A., Mlanda, T., Kimenyi, J. P., Wallace, P., Lukoya, C. O., Hamblion, E. L., & Impouma, B. (2020). Novel approach to support rapid data collection, management, and visualization during the COVID-19 outbreak response in the world health organization African region: development of a data summarization and visualization tool. *JMIR Public Health and Surveillance*, *6*(4), e20355.

Ahmed, S. S., Oviedo-Orta, E., Mekaru, S. R., Freifeld, C. C., Tougas, G., & Brownstein, J. S. (2015). Surveillance for <i>Neisseria meningitidis</i> Disease Activity and Transmission Using Information Technology [Article]. *PLOS ONE*, *10*(5), Article e0127406. <https://doi.org/10.1371/journal.pone.0127406>

Al-Hemoud, A., AlSaraf, M., Malak, M., Al-Shatti, M., Al-Jarba, M., Othman, A., Al-Shammari, H., & Al-Shatti, A. (2021). Analytical and Early Detection System of Infectious Diseases and Animal Health Status in Kuwait. *Frontiers in Veterinary Science*, *8*, 676661.

Albabtain, A. F., AlMulhim, D. A., Yunus, F., & Househ, M. S. (2014). The role of mobile health in the developing world: a review of current knowledge and future trends. *Journal of Selected Areas in Health Informatics*, *4*(2), 10-15.

Allan, M., Lièvre, M., Laurenson-Schafer, H., de Barros, S., Jinnai, Y., Andrews, S., Stricker, T., Formigo, J. P., Schultz, C., & Perrocheau, A. (2022). The World Health Organization COVID-19 surveillance database. *International journal for equity in health*, *21*(Suppl 3), 167.

Asaduzzaman, M., Mekonnen, Z., Rodland, E. K., Sahay, S., Winkler, A. S., & Gradmann, C. (2024). District health information system (DHIS2) as integrated antimicrobial resistance surveillance platform: An exploratory qualitative investigation of the one health stakeholders' viewpoints in Ethiopia [Article

Early Access]. *INTERNATIONAL JOURNAL OF MEDICAL INFORMATICS*, *181*, Article 105268. <https://doi.org/10.1016/j.ijmedinf.2023.105268>

Bashshur, R. L., Mandil, S. H., & Shannon, G. W. (2002). Executive summary [Editorial Material]. *TELEMEDICINE JOURNAL AND E-HEALTH*, *8*(1), 95-107. <https://doi.org/10.1089/15305620252933437>

Blench, M. (2007). Global public health intelligence network (GPHIN). Proceedings of Machine Translation Summit XI: Papers,

Bonkoungou, B., Utunen, H., Talisuna, A. O., O'Connell, G., Koua, E., Chamla, D. D., Arabi, E., Tokar, A., & Gueye, A. S. (2023). Online capacity building for the health workforce: the case of the Integrated Disease Surveillance and Response for the African region [Article]. *JOURNAL OF PUBLIC HEALTH IN AFRICA*, *14*(12), Article 2478. <https://doi.org/10.4081/jphia.2023.2478>

Burner, E., Mercado, J., Hernandez-Saenz, A., Peters, A., Baezconde-Garbanati, L., Arora, S., & Wu, S. (2019). Design and patient characteristics of the randomized controlled trial TExT-MED plus FANS A test of mHealth augmented social support added to a patient-focused text-messaging intervention for emergency department patients with poorly controlled diabetes [Article]. *CONTEMPORARY CLINICAL TRIALS*, *80*, 1-8. <https://doi.org/10.1016/j.cct.2019.03.003>

Carita A. (2014). Healthmap. In *Reference Reviews* (Vol. 28, pp. 30-31). Emerald Group Publishing Limited. <https://doi.org/10.1108/RR-06-2013-0162>

Carter, D., Stojanovic, M., Hachey, P., Fournier, K., Rodier, S., Wang, Y., & de Bruijn, B. (2020, 2020). *Global Public Health Surveillance Using Media Reports: Redesigning GPHIN* [Proceedings Paper]. DIGITAL PERSONALIZED HEALTH AND MEDICINE,

Davis, S. C. (2002). Virtual emergency operations centers. *Risk Management*, *49*(7), 46.

Eggers, C., Martel, L., Dismer, A., Kallay, R., Sayre, D., Choi, M., Corvil, S., Kaba, A., Keita, B., Diallo, L., Balde, M. M., Bah, M., Camara, S. M., Koivogui, E., Montgomery, J., & Keita, S. (2022). Implementing a DHIS2 Ebola virus disease module during the 2021 Guinea Ebola outbreak. *BMJ Glob Health*, *7*(5). <https://doi.org/10.1136/bmjgh-2022-009240>

Freifeld, C. C., Mandl, K. D., Reis, B. Y., & Brownstein, J. S. (2008). HealthMap: global infectious disease monitoring through automated classification and visualization of Internet media reports. *Journal of the American Medical Informatics Association*, *15*(2), 150-157.

George, R., Utunen, H., Ndiaye, N., Tokar, A., Mattar, L., Piroux, C., & Gamhewage, G. (2022). Ensuring equity in access to online courses: Perspectives from the WHO health emergency learning response. *World Medical & Health Policy*, *14*(2), 413-427.

Goldin, S., Kong, S. Y. J., Tokar, A., Utunen, H., Ndiaye, N., Bahl, J., Appuhamy, R., & Moen, A. (2021). Learning From a Massive Open Online COVID-19 Vaccination Training Experience: Survey Study [Article]. *JMIR PUBLIC HEALTH AND SURVEILLANCE*, *7*(12), Article e33455. <https://doi.org/10.2196/33455>

Gülden, B., Mumcuoglu, E., & Baykal, N. (2004, 2004). *A GIS system for ambulatory transportation* [Proceedings Paper]. Proceedings of the Second IASTED International Conference on Biomedical Engineering,

Hugh-Jones, M. (2001). Global awareness of disease outbreaks: the experience of ProMED-mail. *Public Health Reports*, *116*(Suppl 2), 27.

Kaiser, R., Spiegel, P. B., Henderson, A. K., & Gerber, M. L. (2003). The application of geographic information systems and global positioning systems in humanitarian emergencies: lessons learned, programme implications and future research. *Disasters*, *27*(2), 127-140.

Kinkade, C., Russpatrick, S., Potter, R., Saebo, J., Sloan, M., Odongo, G., Singh, T., & Gallagher, K. (2022). Extending and Strengthening Routine DHIS2 Surveillance Systems for COVID-19 Responses in Sierra Leone, Sri Lanka, and Uganda [Article]. *EMERGING INFECTIOUS DISEASES*, *28*, S42-S48. <https://doi.org/10.3201/eid2813.220711>

Litvak, M., Miller, K., Boyle, T., Bedenbaugh, R., Smith, C., Meguerdichian, D., Reisman, D., Biddinger, P., Licurse, A., & Goralnick, E. (2022). Telemedicine use in disasters: a scoping review. *Disaster medicine and public health preparedness*, *16*(2), 791-800.

Lyon, A., Nunn, M., Grossel, G., & Burgman, M. (2012). Comparison of Web-Based Biosecurity Intelligence Systems: BioCaster, EpiSPIDER and HealthMap. *Transboundary and Emerging Diseases*, *59*(3), 223-232. <https://doi.org/https://doi.org/10.1111/j.1865-1682.2011.01258.x>

Ma, J., Huang, Y., & Zheng, Z.-J. (2020). Leveraging the Public Health Emergency Operation Center (PHEOC) for pandemic response: opportunities and challenges [Journal Article

Review]. *Global health journal (Amsterdam, Netherlands)*, *4*(4), 118-120. <https://doi.org/10.1016/j.glohj.2020.11.004>

Mawudeku, A., Blench, M., Boily, L., St. John, R., Andraghetti, R., & Ruben, M. (2013). The global public health intelligence network. *Infectious disease surveillance*, 457-469.

Paulus, D., Meesters, K., & Van de Walle, B. A. (2018). Turning data into action: supporting humanitarian field workers with open data. Iscram,

Rohloff, T., Utunen, H., Renz, J., Zhao, Y., Gamhewage, G., & Meinel, C. (2018). OpenWHO: Integrating Online Knowledge Transfer into Health Emergency Response. EC-TEL (Practitioner Proceedings),

Sulaiman, N., Abid, S. K., Chan, S. W., Nazir, U., Mahmud, N. P. N., Latib, S., Hafidz, H., Shahlal, S., Sapuan, S., & Fernando, T. (2020). Geospatial dashboards for mapping and tracking of novel coronavirus pandemic. Proc. Int. Conf. Ind. Eng. Oper. Manag,

Tanser, F. C., & Le Sueur, D. (2002). The application of geographical information systems to important public health problems in Africa. *International journal of health geographics*, *1*, 1-9.

Utunen, H. (2021). Transferring real-time knowledge free of charge through WHO’s online learning platform OpenWHO. org. *QScience Proceedings*, *2022*(1), 5.

Utunen, H., Attias, M., George, R., O'Connell, G., & Tokar, A. (2022). Learning multiplier effect of OpenWHO. org: use of online learning materials beyond the platform/Effet multiplicateur d'apprentissage de OpenWHO. org: utilisation de materiels d'apprentissage en ligne audela de la plateforme. *Weekly Epidemiological Record*, *97*(1-2), 1-8.

Utunen, H., Ndiaye, N., Attias, M., Mattar, L., Tokar, A., & Gamhewage, G. (2022). Multilingual Approach to COVID-19 Online Learning Response on OpenWHO. org. *Informatics and Technology in Clinical Care and Public Health*, *289*, 192.

Utunen, H., Tokar, A., Dancante, M., & Piroux, C. (2023). Online learning for WHO priority diseases with pandemic potential: evidence from existing courses and preparing for Disease X. *Archives of Public Health*, *81*(1), 61. <https://doi.org/10.1186/s13690-023-01080-9>

Vardell, E. (2020). Global health observatory data repository. *Medical reference services quarterly*, *39*(1), 67-74.

Woodall, J. P. (2001). Global surveillance of emerging diseases: the ProMED-mail perspective. *Cadernos de saude publica*, *17*, S147-S154.

World Organisation for Animal Health. (2023). WAHIS: World Animal Health Information System. <https://wahis.woah.org/#/home>

Ye, J. (2020). The role of health technology and informatics in a global public health emergency: practices and implications from the COVID-19 pandemic. *JMIR medical informatics*, *8*(7), e19866.

Yu, V. L., & Madoff, L. C. (2004). ProMED-mail: an early warning system for emerging diseases. *Clinical Infectious Diseases*, *39*(2), 227-232.

Zaveri, A., Lehmann, J., Auer, S., Hassan, M. M., Sherif, M. A., & Martin, M. (2013). Publishing and interlinking the global health observatory dataset. *Semantic Web*, *4*(3), 315-322.
