# Supplementary material for: Knowledge attributes of public health management information systems used in health emergencies: a scoping review
Source: Front Public Health. 2025 Mar 20;12:1458867. doi: 10.3389/fpubh.2024.1458867 (PMC11969037; doi:10.3389/fpubh.2024.1458867)
Supplement: SUPPLEMENTARY DATA SHEET 3 — Supplementary Tables C1 to C9. [file Data_Sheet_3.zip › SupplementaryTables_C1_C9_KnowledgeAttributesPerHMIS/SupplementaryTable_C7_Immediacy.docx]

**Supplementary table C7: Literary sources for knowledge attributes of HMIS reviewed in the study – Immediacy**.

| **IMS** | **Currently actionable** | **Potentially actionable** |
| --- | --- | --- |
| TACIT Knowledge containing IMS | | |
| GPHIN | (Blench, 2007; Carter et al., 2020; Dion et al., 2015; Keller et al., 2009; Madoff & Li, 2014) |  |
| GLEWS | (Al-Hemoud et al., 2021; Arnoldi et al., 2004; Kshirsagar et al., 2013; Tekola et al., 2017) |  |
| HealthMap | (Ahmed et al., 2015; Bhatia et al., 2021; Hossain & Househ, 2016; Valentin et al., 2023) |  |
| OpenWHO | (Utunen et al., 2023) (Utunen et al., 2018)  (Bonkoungou et al., 2023; George et al., 2020; Samo et al., 2020; Utunen, Attias, et al., 2020; Utunen, George, et al., 2020; Utunen, George, et al., 2021; Utunen et al., 2022; Utunen, Ndiaye, et al., 2021) |  |
| ProMED | (Al-Tawfiq et al., 2014; Aslanov et al., 2017; Babalobi & Cowen, 2005; Carrion & Madoff, 2017; Chang et al., 2022; Madoff & Woodall, 2005; Pollack et al., 2013; Rolland et al., 2020; You et al., 2021; Yuill et al., 2013; Zeldenrust et al., 2008) |  |
| Telemedicine | (Bashshur, 2001; Bashshur et al., 2002; Brown, 2008; Doraiswamy et al., 2020; Khan et al., 2012; Wang et al., 2020; Wright et al., 2022; Ye, 2020) |  |
| mHealth | (Bhattacharya et al., 2018; Grijalva-Eternod et al., 2023; Pande et al., 2012; Rumsfeld et al., 2016; Speciale & Freytsis, 2013; Steinhubl et al., 2019; Vahidi et al., 2021; Varshney, 2014; Williams et al., 2020; Zhang et al., 2020) |  |
| EXPLICIT Knowledge containing IMS | | |
| COVID-19 |  | (Ahmed et al., 2020; Allan et al., 2022; Irwansyah et al., 2020; Ivanković et al., 2021) |
| EOC |  | (Chala et al., 2023; Harris, 2009; Ryan, 2013; World Health Organization, 2014) |
| HDX |  | (Chala et al., 2023; Harris, 2009; Ryan, 2013) |
| DHIS |  | (Bulage et al., 2022; Kadia et al., 2023)  (Dehnavieh et al., 2018) (Garrib et al., 2008)  (Farnham et al., 2020; Kiberu et al., 2014; Raeisi et al., 2013; Tchoualeu et al., 2021) |
| GIS | (Fradelos et al., 2014; Gülden et al., 2004; Jin et al.; Kaiser et al., 2003; Kisiala et al., 2022; Liberg, 2018; Liu & Guo; Maier & Eisner, 2017; McGregor et al., 2005; Pundt et al., 2010; Rocha et al., 2013; Schuler et al., 2022; Tao & Wu; Tsai et al., 2012; Tzavella et al., 2018; Wang et al.; Waring et al., 2005; Yu & Liu; Zhen et al.) |  |
| GHO |  | (Vardell, 2020) |

Ahmed, K., Bukhari, M. A., Mlanda, T., Kimenyi, J. P., Wallace, P., Lukoya, C. O., Hamblion, E. L., & Impouma, B. (2020). Novel approach to support rapid data collection, management, and visualization during the COVID-19 outbreak response in the world health organization African region: development of a data summarization and visualization tool. *JMIR Public Health and Surveillance*, *6*(4), e20355.

Ahmed, S. S., Oviedo-Orta, E., Mekaru, S. R., Freifeld, C. C., Tougas, G., & Brownstein, J. S. (2015). Surveillance for <i>Neisseria meningitidis</i> Disease Activity and Transmission Using Information Technology [Article]. *PLOS ONE*, *10*(5), Article e0127406. <https://doi.org/10.1371/journal.pone.0127406>

Al-Hemoud, A., AlSaraf, M., Malak, M., Al-Shatti, M., Al-Jarba, M., Othman, A., Al-Shammari, H., & Al-Shatti, A. (2021). Analytical and Early Detection System of Infectious Diseases and Animal Health Status in Kuwait. *Frontiers in Veterinary Science*, *8*, 676661.

Al-Tawfiq, J. A., Zumla, A., Gautret, P., Gray, G. C., Hui, D. S., Al-Rabeeah, A. A., & Memish, Z. A. (2014). Emerging respiratory tract infections 1 Surveillance for emerging respiratory viruses [Article]. *LANCET INFECTIOUS DISEASES*, *14*(10), 992-1000. <https://doi.org/10.1016/S1473-3099(14)70840-0>

Allan, M., Lièvre, M., Laurenson-Schafer, H., de Barros, S., Jinnai, Y., Andrews, S., Stricker, T., Formigo, J. P., Schultz, C., & Perrocheau, A. (2022). The World Health Organization COVID-19 surveillance database. *International journal for equity in health*, *21*(Suppl 3), 167.

Arnoldi, J. M., David, M. J., Fernandez, P. J., Fischer, J. R., Frost, B., Lautner, E. A., Marsh, B. D., Taylor, M. A., Thiermann, A. B., & Torres, A. (2004). Report of the USAHA/AAVLD Committee on International Standards. PROCEEDINGS OF THE ANNUAL MEETING-UNITED STATES ANIMAL HEALTH ASSOCIATION,

Aslanov, B., Pshenichnaya, N., Melnik, V., & Rakhmanova, N. (2017). Promed-mail: internet-based surveillance system for emerging infectious diseases. *Профилактическая и клиническая медицина*(2), 54-59.

Babalobi, O., & Cowen, P. (2005). PROMED–mail: an electronic mail disease-reporting: a case study.

Bashshur, R. L. (2001). Where we are in telemedicine/telehealth, and where we go from here [Editorial Material]. *TELEMEDICINE JOURNAL AND E-HEALTH*, *7*(4), 273-277. <https://doi.org/10.1089/15305620152814665>

Bashshur, R. L., Mandil, S. H., & Shannon, G. W. (2002). Executive summary [Editorial Material]. *TELEMEDICINE JOURNAL AND E-HEALTH*, *8*(1), 95-107. <https://doi.org/10.1089/15305620252933437>

Bhatia, S., Lassmann, B., Cohn, E., Desai, A. N., Carrion, M., Kraemer, M. U. G., Herringer, M., Brownstein, J., Madoff, L., Cori, A., & Nouvellet, P. (2021). Using digital surveillance tools for near real-time mapping of the risk of infectious disease spread [Article]. *NPJ DIGITAL MEDICINE*, *4*(1), Article 73. <https://doi.org/10.1038/s41746-021-00442-3>

Bhattacharya, S., Kumar, A., Kaushal, V., & Singh, A. (2018). Applications of m-Health and e-Health in Public Health Sector: the challenges and opportunities. *International Journal of medicine and public Health*, *8*(2).

Blench, M. (2007). Global public health intelligence network (GPHIN). Proceedings of Machine Translation Summit XI: Papers,

Bonkoungou, B., Utunen, H., Talisuna, A. O., O'Connell, G., Koua, E., Chamla, D. D., Arabi, E., Tokar, A., & Gueye, A. S. (2023). Online capacity building for the health workforce: the case of the Integrated Disease Surveillance and Response for the African region [Article]. *JOURNAL OF PUBLIC HEALTH IN AFRICA*, *14*(12), Article 2478. <https://doi.org/10.4081/jphia.2023.2478>

Brown, E. V. (2008). Robotic assistance remedy. The Michigan Stroke Network utilizes remote presence robots to bring needed specialists to stroke patients at remote hospitals [Journal Article]. *Health management technology*, *29*(7), 18-21.

Bulage, L., Kadobera, D., Kwesiga, B., Kabwama, S. N., Ario, A. R., & Harris, J. R. (2022). Delayed outbreak detection: a wake-up call to evaluate a surveillance system. *Pan Afr Med J*, *41*(Suppl 1), 1. <https://doi.org/10.11604/pamj.supp.2022.41.1.31161>

Carrion, M., & Madoff, L. C. (2017). ProMED-mail: 22 years of digital surveillance of emerging infectious diseases. *International Health*, *9*(3), 177-183. <https://doi.org/10.1093/inthealth/ihx014>

Carter, D., Stojanovic, M., Hachey, P., Fournier, K., Rodier, S., Wang, Y., & de Bruijn, B. (2020, 2020). *Global Public Health Surveillance Using Media Reports: Redesigning GPHIN* [Proceedings Paper]. DIGITAL PERSONALIZED HEALTH AND MEDICINE,

Chala, T. K., Abera, E. G., Tukeni, K. N., Didu, G. H., Abbagidi, F. A., Yesuf, E. A., Yilma, D., & Gudina, E. K. (2023). The Need to Establish and Sustain Public Health Emergency Operation Centers for Managing Infectious Disease Outbreaks: Lesson From Response to Louse-Borne Relapsing Fever Outbreak in Jimma, Ethiopia [Article]. *DISASTER MEDICINE AND PUBLIC HEALTH PREPAREDNESS*, *17*, Article e535. <https://doi.org/10.1017/dmp.2023.192>

Chang, Y. C., Chiu, Y. W., & Chuang, T. W. (2022). Linguistic Pattern-Infused Dual-Channel Bidirectional Long Short-term Memory With Attention for Dengue Case Summary Generation From the Program for Monitoring Emerging Diseases-Mail Database: Algorithm Development Study [Article]. *JMIR PUBLIC HEALTH AND SURVEILLANCE*, *8*(7), Article e34583. <https://doi.org/10.2196/34583>

Dehnavieh, R., Haghdoost, A., Khosravi, A., Hoseinabadi, F., Rahimi, H., Poursheikhali, A., Khajehpour, N., Khajeh, Z., Mirshekari, N., Hasani, M., Radmerikhi, S., Haghighi, H., Mehrolhassani, M. H., Kazemi, E., & Aghamohamadi, S. (2018). The District Health Information System (DHIS2): A literature review and meta-synthesis of its strengths and operational challenges based on the experiences of 11 countries. *Health Information Management Journal*, *48*(2), 62-75. <https://doi.org/10.1177/1833358318777713>

Dion, M., AbdelMalik, P., & Mawudeku, A. (2015). Big Data and the Global Public Health Intelligence Network (GPHIN). *Can Commun Dis Rep*, *41*(9), 209-214. <https://doi.org/10.14745/ccdr.v41i09a02>

Doraiswamy, S., Abraham, A., Mamtani, R., & Cheema, S. (2020). *Use of telemedicine/ telehealth for geriatric care during the COVID-19 pandemic - A scoping review and evidence mapping*. <https://doi.org/10.17605/OSF.IO/26Z74>

Farnham, A., Utzinger, J., Kulinkina, A. V., & Winkler, M. S. (2020). Using district health information to monitor sustainable development. *Bull World Health Organ*, *98*(1), 69-71. <https://doi.org/10.2471/blt.19.239970>

Fradelos, E. C., Papathanasiou, I. V., Mitsi, D., Tsaras, K., Kleisiaris, C. F., & Kourkouta, L. (2014). Health based geographic information systems (GIS) and their applications. *Acta Informatica Medica*, *22*(6), 402.

Garrib, A., Stoops, N., McKenzie, A., Dlamini, L., Govender, T., Rohde, J., & Herbst, K. (2008). An evaluation of the District Health Information System in rural South Africa. *S Afr Med J*, *98*(7), 549-552.

George, R., Utunen, H., Attias, M., Sy, A., Ndiaye, N., Piroux, C., & Gamhewage, G. (2020, 2020). *An Analysis of the Growth in Uptake of OpenWHO's Online Learning Resources for COVID-19* [Proceedings Paper]. IMPORTANCE OF HEALTH INFORMATICS IN PUBLIC HEALTH DURING A PANDEMIC,

Grijalva-Eternod, C. S., Jelle, M., Mohamed, H., Waller, K., Osman Hussein, B., Barasa, E., Solomon, A., Mehjabeen, S., Copas, A., Fottrell, E., & Seal, A. J. (2023). Evaluation of conditional cash transfers and mHealth audio messaging in reduction of risk factors for childhood malnutrition in internally displaced persons camps in Somalia: A 2 × 2 factorial cluster-randomised controlled trial. *PLOS Medicine*, *20*(2), e1004180. <https://doi.org/10.1371/journal.pmed.1004180>

Gülden, B., Mumcuoglu, E., & Baykal, N. (2004, 2004). *A GIS system for ambulatory transportation* [Proceedings Paper]. Proceedings of the Second IASTED International Conference on Biomedical Engineering,

Harris, E. A. (2009). *The use of networks to connect local emergency operations centers*. Oklahoma State University.

Hossain, N., & Househ, M. S. (2016). Using HealthMap to Analyse Middle East Respiratory Syndrome (MERS) Data. ICIMTH,

Irwansyah, E., Budiharto, W., Widhyatmoko, D., Istamar, A., & Panghurian, F. P. (2020). Monitoring Coronavirus COVID-19/SARS-CoV-2 Pandemic using GIS Dashboard: International and Indonesia Context. *Preprints* <https://doi.org/10.20944/preprints202008.0415.v1> I

Ivanković, D., Barbazza, E., Bos, V., Brito Fernandes, Ó., Jamieson Gilmore, K., Jansen, T., Kara, P., Larrain, N., Lu, S., & Meza-Torres, B. (2021). Features constituting actionable COVID-19 dashboards: descriptive assessment and expert appraisal of 158 public web-based COVID-19 dashboards. *Journal of medical Internet research*, *23*(2), e25682.

Jin, L., Li, Q., & Niu, Y. *Emergent public health event processing and displaying method, involves determining public sanitary event corresponding to burst response level, and determining burst public health event response level by electronic map display* CN110084730-A).

Kadia, R. S. M., Kadia, B. M., Dimala, C. A., & Collins, A. E. (2023). Usefulness of disease surveillance data in enhanced early warning of the cholera outbreak in Southwest Cameroon, 2018. *Conflict and Health*, *17*(1), 6. <https://doi.org/10.1186/s13031-023-00504-1>

Kaiser, R., Spiegel, P. B., Henderson, A. K., & Gerber, M. L. (2003). The application of geographic information systems and global positioning systems in humanitarian emergencies: lessons learned, programme implications and future research. *Disasters*, *27*(2), 127-140.

Keller, M., Blench, M., Tolentino, H., Freifeld, C. C., Mandl, K. D., Mawudeku, A., Eysenbach, G., & Brownstein, J. S. (2009). Use of Unstructured Event-Based Reports for Global Infectious Disease Surveillance [Article]. *EMERGING INFECTIOUS DISEASES*, *15*(5), 689-695. <https://doi.org/10.3201/eid1505.081114>

Khan, B., Hiratsuka, V. Y., Dillard, D., Robinson, R., & Mau, M. (2012). Availability and Deployment of Telemedicine/Telehealth Technologies in Rural Alaska [Journal Article]. *Federal practitioner : for the health care professionals of the VA, DoD, and PHS*, *29*(12), 19-21.

Kiberu, V. M., Matovu, J. K. B., Makumbi, F., Kyozira, C., Mukooyo, E., & Wanyenze, R. K. (2014). Strengthening district-based health reporting through the district health management information software system: the Ugandan experience. *BMC Medical Informatics and Decision Making*, *14*(1), 40. <https://doi.org/10.1186/1472-6947-14-40>

Kisiala, W., Racka, I., & Suszynska, K. (2022). Population Access to Hospital Emergency Departments: The Spatial Analysis in Public Health Research [Article]. *INTERNATIONAL JOURNAL OF ENVIRONMENTAL RESEARCH AND PUBLIC HEALTH*, *19*(3), Article 1437. <https://doi.org/10.3390/ijerph19031437>

Kshirsagar, D., Savalia, C., Kalyani, I., Kumar, R., & Nayak, D. (2013). Disease alerts and forecasting of zoonotic diseases: an overview. *Veterinary World*, *6*(11), 889.

Liberg, R. B. (2018). USING GEOGRAPHIC INFORMATION SYSTEMS IN RURAL EMERGENCY MEDICAL SERVICES: REDUCING RESPONSE TIMES BY REALLOCATING RESOURCES [Meeting Abstract]. *JOURNAL OF INVESTIGATIVE MEDICINE*, *66*(1), 104-104. <https://doi.org/10.1136/jim-2017-000663.94>

Liu, J., & Guo, M. *Infectious disease monitoring and pre-warning system for use in public health emergencies, has data storage center for storing and collecting data from hospital diagnosis monitoring module, drugstore medicine sale monitoring module, and infectious disease history analysis data* CN117174332-A).

Madoff, L. C., & Li, A. (2014). Web-Based Surveillance Systems for Human, Animal, and Plant Diseases [Journal Article

Review]. *Microbiology spectrum*, *2*(1), OH-0015-2012. <https://doi.org/10.1128/microbiolspec.OH-0015-2012>

Madoff, L. C., & Woodall, J. P. (2005). The Internet and the Global Monitoring of Emerging Diseases: Lessons from the First 10 Years of ProMED-mail. *Archives of Medical Research*, *36*(6), 724-730. <https://doi.org/https://doi.org/10.1016/j.arcmed.2005.06.005>

Maier, N. M., & Eisner, G. R. (2017). *Method for locating internet of things network devices e.g. baby monitors, during e.g. health event, involves displaying current physical geographic location for network device for desired emergency response agencies on graphical map* US2017238129-A1

US10511950-B2).

McGregor, J., Hanlon, N., Emmons, S., Voaklander, D., & Kelly, K. (2005). If all ambulances could fly: putting provincial standards of emergency care access to the test in Northern British Columbia [Journal Article

Research Support, Non-U.S. Gov't]. *Canadian journal of rural medicine : the official journal of the Society of Rural Physicians of Canada = Journal canadien de la medecine rurale : le journal officiel de la Societe de medecine rurale du Canada*, *10*(3), 163-168.

Pande, A., Kimbahune, S., Bondale, N., Shinde, R., Shanbhag, S., & Ramaswamy, S. (2012, 2012). *Distributed Processing and Internet Technology to Solve Challenges of Primary Healthcare in India* [Proceedings Paper]. DISTRIBUTED COMPUTING AND INTERNET TECHNOLOGY,

Pollack, M. P., Pringle, C., Madoff, L. C., & Memish, Z. A. (2013). Latest outbreak news from ProMED-mail: novel coronavirus–Middle East. *International Journal of Infectious Diseases*, *17*(2), e143-e144.

Pundt, H., Spangenberg, T., & Weinkauf, R. (2010, 2010). *WEB-BASED AND CONTEXT-SENSITIVE, MOBILE GEO-TOOLS TO SUPPORT SPATIAL DECISION MAKING IN HEALTH AND EMERGENCY MANAGEMENT* [Proceedings Paper]. HEALTHINF 2010: PROCEEDINGS OF THE THIRD INTERNATIONAL CONFERENCE ON HEALTH INFORMATICS,

Raeisi, A. R., Saghaeiannejad, S., Karimi, S., Ehteshami, A., & Kasaei, M. (2013). District health information system assessment: a case study in iran. *Acta Inform Med*, *21*(1), 30-35. <https://doi.org/10.5455/aim.2012.21.30-35>

Rocha, C. M., Kruger, E., McGuire, S., & Tennant, M. (2013). The geographic distribution of patients seeking emergency dental care at the Royal Dental Hospital of Melbourne, Australia [Article]. *COMMUNITY DENTAL HEALTH*, *30*(3), 149-154. <https://doi.org/10.1922/CDH_3124Kruger06>

Rolland, C., Lazarus, C., Giese, C., Monate, B., Travert, A. S., & Salomon, J. (2020). Early Detection of Public Health Emergencies of International Concern through Undiagnosed Disease Reports in ProMED-Mail. *Emerg Infect Dis*, *26*(2), 336-339. <https://doi.org/10.3201/eid2602.191043>

Rumsfeld, J. S., Brooks, S. C., Aufderheide, T. P., Leary, M., Bradley, S. M., Nkonde-Price, C., Schwamm, L. H., Jessup, M., Ferrer, J. M. E., Merchant, R. M., Amer Heart Assoc Emergency, C., Council Cardiopulm Critical Care, P., Council Quality Care Outcomes, R., Council Cardiovasc Stroke, N., & Council Epidemiology, P. (2016). Use of Mobile Devices, Social Media, and Crowdsourcing as Digital Strategies to Improve Emergency Cardiovascular Care A Scientific Statement From the American Heart Association [Article]. *CIRCULATION*, *134*(8), E87-E108. <https://doi.org/10.1161/CIR.0000000000000428>

Ryan, M. (2013). Planning in the emergency operations center. *Technological forecasting and social change*, *80*(9), 1725-1731.

Samo, G., Zhao, U. Y., & Gamhewage, G. (2020). Syntactic Complexity of Learning Content in Italian for COVID-19 Frontline Responders: A Study on WHO’s Emergency Learning Platform. *Verbum*, *11*.

Schuler, F., Ma, M., & Perkins, J. (2022). *System for determining priority discrepancies between audio data and records data, has electronic computing device for generating geographical map that includes priority discrepancies, and is configured for display on display device* WO2022213023-A1

US2022318278-A1).

Speciale, A. M., & Freytsis, M. (2013). mHealth for Midwives: A Call to Action [Review]. *JOURNAL OF MIDWIFERY & WOMENS HEALTH*, *58*(1), 76-82. <https://doi.org/10.1111/j.1542-2011.2012.00243.x>

Steinhubl, S. R., Edwards, A. M., Waalen, J., Zambon, R., Mehta, R., Ariniello, L., Ebner, G., Baca-Motes, K., Carter, C., Felicione, E., Sarich, T., & Topol, E. (2019). HEALTHCARE RESOURCE UTILIZATION ASSOCIATED WITH ELECTROCARDIOGRAPH (ECG) SENSOR PATCH SCREENING FOR ATRIAL FIBRILLATION (AF): RESULTS FROM THE MHEALTH SCREENING TO PREVENT STROKES (MSTOPS) TRIAL [Meeting Abstract]. *JOURNAL OF THE AMERICAN COLLEGE OF CARDIOLOGY*, *73*(9), 296-296. <https://doi.org/10.1016/S0735-1097(19)30904-0>

Tao, Y., & Wu, P. *Emergency evacuation method for evacuating people urgently to ensure safety of life and property of people, based on geographic information system (GIS) in event type such as natural disaster type, involves sending emergency evacuation information to target mobile device* CN117082450-A).

Tchoualeu, D. D., Elmousaad, H. E., Osadebe, L. U., Adegoke, O. J., Nnadi, C., Haladu, S. A., Jacenko, S. M., Davis, L. B., Bloland, P. B., & Sandhu, H. S. (2021). Use of a district health information system 2 routine immunization dashboard for immunization program monitoring and decision making, Kano State, Nigeria. *The Pan African Medical Journal*, *40*(Suppl 1).

Tekola, B., Myers, L., Lubroth, J., Plee, L., Calistri, P., & Pinto, J. (2017). International health threats and global early warning and response mechanisms [Article]. *REVUE SCIENTIFIQUE ET TECHNIQUE-OFFICE INTERNATIONAL DES EPIZOOTIES*, *36*(2), 657-670. <https://doi.org/10.20506/rst.36.2.2683>

Tsai, M.-K., Lee, Y.-C., Lu, C.-H., Chen, M.-H., Chou, T.-Y., & Yau, N.-J. (2012). Integrating geographical information and augmented reality techniques for mobile escape guidelines on nuclear accident sites [Article]. *JOURNAL OF ENVIRONMENTAL RADIOACTIVITY*, *109*, 36-44. <https://doi.org/10.1016/j.jenvrad.2011.12.025>

Tzavella, K., Fekete, A., & Fiedrich, F. (2018). Opportunities provided by geographic information systems and volunteered geographic information for a timely emergency response during flood events in Cologne, Germany. *Natural Hazards*, *91*, 29-57.

Utunen, H., Attias, M., George, R., Ndiaye, N., Piroux, C., Farzi, M. R., Sy, A., & Gamhewage, G. (2020, 2020). *Global Access to OpenWHO's Online Learning Resources for COVID-19* [Proceedings Paper]. IMPORTANCE OF HEALTH INFORMATICS IN PUBLIC HEALTH DURING A PANDEMIC,

Utunen, H., Christen, P., Gamhewage, G. M., Zhao, U., & Attias, M. (2018). Knowledge transfer for Ebola outbreak–production and use of OpenWHO. org online learning resources. 2018 14th International Conference on Wireless and Mobile Computing, Networking and Communications (WiMob),

Utunen, H., George, R., Ndiaye, N., Attias, M., Piroux, C., & Gamhewage, G. (2020). Responding to global learning needs during a pandemic: an analysis of the trends in platform use and incidence of COVID-19. *Education Sciences*, *10*(11), 345.

Utunen, H., George, R., Ndiaye, N., Tokar, A., Attias, M., & Gamhewage, G. (2021). Delivering WHO’s life-saving information in real-time during a pandemic through an online learning platform: evidence from global use. In *Public Health and Informatics* (pp. 969-973). IOS Press.

Utunen, H., Mattar, L., Piroux, C., Ndiaye, N., Christen, P., & Attias, M. (2022). Superusers of self-paced online learning on OpenWHO.

Utunen, H., Ndiaye, N., Mattar, L., Christen, P., Stucke, O., & Gamhewage, G. (2021). Changes in Users Trends Before and During the COVID-19 Pandemic on WHO's Online Learning Platform [Journal Article]. *Studies in health technology and informatics*, *287*, 163-164. <https://doi.org/10.3233/SHTI210838>

Utunen, H., Tokar, A., Dancante, M., & Piroux, C. (2023). Online learning for WHO priority diseases with pandemic potential: evidence from existing courses and preparing for Disease X. *Archives of Public Health*, *81*(1), 61. <https://doi.org/10.1186/s13690-023-01080-9>

Vahidi, H., Taleai, M., Yan, W., & Shaw, R. (2021). Digital Citizen Science for Responding to COVID-19 Crisis: Experiences from Iran [Article]. *INTERNATIONAL JOURNAL OF ENVIRONMENTAL RESEARCH AND PUBLIC HEALTH*, *18*(18), Article 9666. <https://doi.org/10.3390/ijerph18189666>

Valentin, S., Boudoua, B., Sewalk, K., Arınık, N., Roche, M., Lancelot, R., & Arsevska, E. (2023). Dissemination of information in event-based surveillance, a case study of Avian Influenza. *PLOS ONE*, *18*(9), e0285341. <https://doi.org/10.1371/journal.pone.0285341>

Vardell, E. (2020). Global health observatory data repository. *Medical reference services quarterly*, *39*(1), 67-74.

Varshney, U. (2014). Mobile health: Four emerging themes of research. *Decision Support Systems*, *66*, 20-35.

Wang, Y., Li, B., & Liu, L. (2020). Telemedicine experience in China: our response to the pandemic and current challenges. *Frontiers in Public Health*, *8*, 549669.

Wang, Z., Li, H., Song, J., Gong, X., Chen, N., Song, C., & Lu, Y. *Public health event emergency medical facility addressing method based on point of interest data, involves establishing a file geographic information database by using a file geographic information system software* CN112232599-A).

Waring, S., Zakos-Feliberti, A., Wood, R., Stone, M., Padgett, P., & Arafat, R. (2005). The utility of geographic information systems (GIS) in rapid epidemiological assessments following weather-related disasters: methodological issues based on the Tropical Storm Allison Experience. *International journal of hygiene and environmental health*, *208*(1-2), 109-116.

Williams, S. Y., Adeyemi, S. O., Eyitayo, J. O., Odeyemi, O. E., Dada, O. E., Adesina, M. A., & Akintayo, A. D. (2020). Mobile health technology (Mhealth) in combating COVID-19 pandemic: Use, challenges and recommendations. *Electronic Journal of Medical and Educational Technologies*, *13*(4), em2018.

World Health Organization. (2014). A systematic review of public health emergency operations centres (EOC): December 2013. <https://www.who.int/publications/i/item/WHO-HSE-GCR-2014.1>

Wright, S., Spaulding, R., & Henley, W. (2022). A Multipronged Digital Response to Increased Demand for Telehealth Support and Training During the COVID-19 Pandemic [Case Reports]. *Journal of technology in behavioral science*, *7*(1), 73-80. <https://doi.org/10.1007/s41347-021-00224-4>

Ye, J. (2020). The role of health technology and informatics in a global public health emergency: practices and implications from the COVID-19 pandemic. *JMIR medical informatics*, *8*(7), e19866.

You, J., Expert, P., & Costelloe, C. (2021). Using text mining to track outbreak trends in global surveillance of emerging diseases: ProMED-mail. *Journal of the Royal Statistical Society Series A: Statistics in Society*, *184*(4), 1245-1259.

Yu, Z., & Liu, P. *Method for locating geographical position of graph convolution network model based on attention aggregate, involves inputting result obtained by training graph convolution network model to geographic position predictor of multilayer sensor* CN116166865-A).

Yuill, T. M., Woodall, J. P., & Baekeland, S. (2013). Latest outbreak news from ProMED-mail. Yellow fever outbreak-Darfur Sudan and Chad [Review]. *INTERNATIONAL JOURNAL OF INFECTIOUS DISEASES*, *17*(7), E476-E478. <https://doi.org/10.1016/j.ijid.2013.03.009>

Zeldenrust, M., Rahamat-Langendoen, J., Postma, M., & Van Vliet, J. (2008). The value of ProMED-mail for the Early Warning Committee in the Netherlands: more specific approach recommended. *Eurosurveillance*, *13*(6), 7-8.

Zhang, Z., Zhang, L., Zheng, J., Xiao, H., & Li, Z. (2020). COVID-19-Related Disruptions and Increased mHealth Emergency Use Intention: Experience Sampling Method Study [Article]. *JMIR MHEALTH AND UHEALTH*, *8*(12), Article e20642. <https://doi.org/10.2196/20642>

Zhen, W., Li, C., Chen, R., Wei, Z., Chen, T., & Yan, J. *Multi-scene city emergency sampling site addressing optimization method for city public health safety technical field, involves obtaining mobile phone signaling data of target city, interest point, and basic geographic information data* CN115860213-A).
