# Supplementary material for: Knowledge attributes of public health management information systems used in health emergencies: a scoping review
Source: Front Public Health. 2025 Mar 20;12:1458867. doi: 10.3389/fpubh.2024.1458867 (PMC11969037; doi:10.3389/fpubh.2024.1458867)
Supplement: SUPPLEMENTARY DATA SHEET 3 — Supplementary Tables C1 to C9. [file Data_Sheet_3.zip › SupplementaryTables_C1_C9_KnowledgeAttributesPerHMIS/SupplementaryTable_C8_Practical.docx]

**Supplementary table C8: Literary sources for knowledge attributes of HMIS reviewed in the study – Practicability.**

| **IMS** | **Highly practical** | **Moderately practical** |
| --- | --- | --- |
| TACIT Knowledge containing IMS | | |
| GPHIN | (Mawudeku et al., 2013; Mykhalovskiy & Weir, 2006; Young et al., 2015) |  |
| GLEWS |  | (Lin et al., 2023; Tounkara et al., 2019) |
| HealthMap | (Ahmed et al., 2015; Bhatia et al., 2021; Ganser, 2020; Valentin et al., 2023) |  |
| OpenWHO | (George et al., 2022; Utunen, Appuhamy, et al., 2023; Utunen, Tokar, et al., 2023) (Bonkoungou et al., 2023) |  |
| ProMED | (Al-Tawfiq et al., 2014; Chang et al., 2022) |  |
| Telemedicine | (Bokolo, 2023; Greiwe, 2022; Kilova & Uzunova, 2020; Leite & Hodgkinson, 2021; Litvak et al., 2022; Loeb et al., 2020; Mahtta et al., 2021; Maleka & Matli, 2022; Nachiket et al., 2021; Rockwell & Gilroy, 2020) (Bashshur, 2001; Bashshur et al., 2002; Doarn & Merrell, 2019; Doraiswamy et al., 2020; Khan et al., 2012; Wright et al., 2022) |  |
| mHealth | (Eberle et al., 2021; Jabeen & Mohammad, 2023; Rumsfeld et al., 2016; Speciale & Freytsis, 2013; Steinhubl et al., 2019; Tamrat & Kachnowski, 2012; Vahidi et al., 2021) |  |
| EXPLICIT Knowledge containing IMS | | |
| COVID-19 | (Ahmed et al., 2020; Irwansyah et al., 2020) |  |
| EOC | (Chala et al., 2023; Kayiwa et al., 2022; Ma et al., 2020; Su et al., 2017) |  |
| HDX | (Telford, 2020) |  |
| DHIS |  | (Abajebel et al., 2011; Joseph et al., 2022; Lungo, 2008; Odei-Lartey et al., 2020; Odhiambo-Otieno, 2005; Wangdi et al., 2020) |
| GIS | (Gülden et al., 2004; Jin et al.; Kisiala et al., 2022; Li et al., 2013; Liberg, 2018; Liu & Guo; Maier & Eisner, 2017; McGregor et al., 2005; Nykiforuk & Flaman, 2011; Pundt et al., 2010; Rocha et al., 2013; Schuler et al., 2022; Shaw & McGuire, 2017; Tao & Wu; Tsai et al., 2012; Wang et al.; Yu & Liu; Zhen et al.) |  |
| GHO |  | (Vardell, 2020) |

Abajebel, S., Jira, C., & Beyene, W. (2011). Utilization of health information system at district level in jimma zone oromia regional state, South west ethiopia. *Ethiop J Health Sci*, *21*(Suppl 1), 65-76.

Ahmed, K., Bukhari, M. A., Mlanda, T., Kimenyi, J. P., Wallace, P., Lukoya, C. O., Hamblion, E. L., & Impouma, B. (2020). Novel approach to support rapid data collection, management, and visualization during the COVID-19 outbreak response in the world health organization African region: development of a data summarization and visualization tool. *JMIR Public Health and Surveillance*, *6*(4), e20355.

Ahmed, S. S., Oviedo-Orta, E., Mekaru, S. R., Freifeld, C. C., Tougas, G., & Brownstein, J. S. (2015). Surveillance for <i>Neisseria meningitidis</i> Disease Activity and Transmission Using Information Technology [Article]. *PLOS ONE*, *10*(5), Article e0127406. <https://doi.org/10.1371/journal.pone.0127406>

Al-Tawfiq, J. A., Zumla, A., Gautret, P., Gray, G. C., Hui, D. S., Al-Rabeeah, A. A., & Memish, Z. A. (2014). Emerging respiratory tract infections 1 Surveillance for emerging respiratory viruses [Article]. *LANCET INFECTIOUS DISEASES*, *14*(10), 992-1000. <https://doi.org/10.1016/S1473-3099(14)70840-0>

Bashshur, R. L. (2001). Where we are in telemedicine/telehealth, and where we go from here [Editorial Material]. *TELEMEDICINE JOURNAL AND E-HEALTH*, *7*(4), 273-277. <https://doi.org/10.1089/15305620152814665>

Bashshur, R. L., Mandil, S. H., & Shannon, G. W. (2002). Executive summary [Editorial Material]. *TELEMEDICINE JOURNAL AND E-HEALTH*, *8*(1), 95-107. <https://doi.org/10.1089/15305620252933437>

Bhatia, S., Lassmann, B., Cohn, E., Desai, A. N., Carrion, M., Kraemer, M. U. G., Herringer, M., Brownstein, J., Madoff, L., Cori, A., & Nouvellet, P. (2021). Using digital surveillance tools for near real-time mapping of the risk of infectious disease spread [Article]. *NPJ DIGITAL MEDICINE*, *4*(1), Article 73. <https://doi.org/10.1038/s41746-021-00442-3>

Bokolo, A. J. (2023). Investigating the implementation of telehealth and digital technologies during public health crisis: A qualitative review. *The International Journal of Health Planning and Management*, *38*(5), 1212-1227. <https://doi.org/https://doi.org/10.1002/hpm.3681>

Bonkoungou, B., Utunen, H., Talisuna, A. O., O'Connell, G., Koua, E., Chamla, D. D., Arabi, E., Tokar, A., & Gueye, A. S. (2023). Online capacity building for the health workforce: the case of the Integrated Disease Surveillance and Response for the African region [Article]. *JOURNAL OF PUBLIC HEALTH IN AFRICA*, *14*(12), Article 2478. <https://doi.org/10.4081/jphia.2023.2478>

Chala, T. K., Abera, E. G., Tukeni, K. N., Didu, G. H., Abbagidi, F. A., Yesuf, E. A., Yilma, D., & Gudina, E. K. (2023). The Need to Establish and Sustain Public Health Emergency Operation Centers for Managing Infectious Disease Outbreaks: Lesson From Response to Louse-Borne Relapsing Fever Outbreak in Jimma, Ethiopia [Article]. *DISASTER MEDICINE AND PUBLIC HEALTH PREPAREDNESS*, *17*, Article e535. <https://doi.org/10.1017/dmp.2023.192>

Chang, Y. C., Chiu, Y. W., & Chuang, T. W. (2022). Linguistic Pattern-Infused Dual-Channel Bidirectional Long Short-term Memory With Attention for Dengue Case Summary Generation From the Program for Monitoring Emerging Diseases-Mail Database: Algorithm Development Study [Article]. *JMIR PUBLIC HEALTH AND SURVEILLANCE*, *8*(7), Article e34583. <https://doi.org/10.2196/34583>

Doarn, C. R., & Merrell, R. C. (2019). Telemedicine, Telehealth, and the Public Health Good [Editorial Material]. *TELEMEDICINE AND E-HEALTH*, *25*(9), 773-774. <https://doi.org/10.1089/tmj.2019.29028.crd>

Doraiswamy, S., Abraham, A., Mamtani, R., & Cheema, S. (2020). *Use of telemedicine/ telehealth for geriatric care during the COVID-19 pandemic - A scoping review and evidence mapping*. <https://doi.org/10.17605/OSF.IO/26Z74>

Eberle, C., Loehnert, M., & Stichling, S. (2021). Effectivness of specific mobile health applications (mHealth-apps) in gestational diabtetes mellitus: a systematic review [Review]. *BMC PREGNANCY AND CHILDBIRTH*, *21*(1), Article 808. <https://doi.org/10.1186/s12884-021-04274-7>

Ganser, I. Z. (2020). *Evaluation of Event-Based Internet Biosurveillance for Multi-Regional Detection of Seasonal Influenza Onset* [Dissertation/Thesis].

George, R., Utunen, H., Ndiaye, N., Tokar, A., Mattar, L., Piroux, C., & Gamhewage, G. (2022). Ensuring equity in access to online courses: Perspectives from the WHO health emergency learning response. *World Medical & Health Policy*, *14*(2), 413-427.

Greiwe, J. (2022). Telemedicine Lessons Learned During the COVID-19 Pandemic. *Current Allergy and Asthma Reports*, *22*(1), 1-5. <https://doi.org/10.1007/s11882-022-01026-1>

Gülden, B., Mumcuoglu, E., & Baykal, N. (2004, 2004). *A GIS system for ambulatory transportation* [Proceedings Paper]. Proceedings of the Second IASTED International Conference on Biomedical Engineering,

Irwansyah, E., Budiharto, W., Widhyatmoko, D., Istamar, A., & Panghurian, F. P. (2020). Monitoring Coronavirus COVID-19/SARS-CoV-2 Pandemic using GIS Dashboard: International and Indonesia Context. *Preprints* <https://doi.org/10.20944/preprints202008.0415.v1> I

Jabeen, R., & Mohammad, A. (2023). Leverage of mHealth integration in Maternal and child health services and COVID-19 Pandemic [Article]. *JOURNAL OF THE PAKISTAN MEDICAL ASSOCIATION*, *73*(2), 370-373. <https://doi.org/10.47391/JPMA.5156>

Jin, L., Li, Q., & Niu, Y. *Emergent public health event processing and displaying method, involves determining public sanitary event corresponding to burst response level, and determining burst public health event response level by electronic map display* CN110084730-A).

Joseph, J. J., Mkali, H. R., Reaves, E. J., Mwaipape, O. S., Mohamed, A., Lazaro, S. N., Aaron, S., Chacky, F., Mahendeka, A., Rulagirwa, H. S., Mwenesi, M., Mwakapeje, E., Ally, A. Y., Kitojo, C., Serbantez, N., Nyinondi, S., Lalji, S. M., Wilillo, R., Al-mafazy, A.-w., . . . Ngondi, J. M. (2022). Improvements in malaria surveillance through the electronic Integrated Disease Surveillance and Response (eIDSR) system in mainland Tanzania, 2013–2021. *Malaria Journal*, *21*(1), 321. <https://doi.org/10.1186/s12936-022-04353-w>

Kayiwa, J., Homsy, J., Nelson, L. J., Ocom, F., Kasule, J. N., Wetaka, M. M., Kyazze, S., Mwanje, W., Kisakye, A., Nabunya, D., Nyirabakunzi, M., Aliddeki, D. M., Ojwang, J., Boore, A., Kasozi, S., Borchert, J., Shoemaker, T., Nabatanzi, S., Dahlke, M., . . . Makumbi, I. (2022). Establishing a Public Health Emergency Operations Center in an Outbreak-Prone Country: Lessons Learned in Uganda, January 2014 to December 2021 [Article

Early Access]. *HEALTH SECURITY*, *20*(5), 394-407. <https://doi.org/10.1089/hs.2022.0048>

Khan, B., Hiratsuka, V. Y., Dillard, D., Robinson, R., & Mau, M. (2012). Availability and Deployment of Telemedicine/Telehealth Technologies in Rural Alaska [Journal Article]. *Federal practitioner : for the health care professionals of the VA, DoD, and PHS*, *29*(12), 19-21.

Kilova, K., & Uzunova, S. (2020). Telemedicine in assistance to healthcare in the COVID-19 pandemic. *Acta Medica Bulgarica*, *47*(4), 63-68.

Kisiala, W., Racka, I., & Suszynska, K. (2022). Population Access to Hospital Emergency Departments: The Spatial Analysis in Public Health Research [Article]. *INTERNATIONAL JOURNAL OF ENVIRONMENTAL RESEARCH AND PUBLIC HEALTH*, *19*(3), Article 1437. <https://doi.org/10.3390/ijerph19031437>

Leite, H., & Hodgkinson, I. R. (2021). Telemedicine co‐design and value co‐creation in public health care. *Australian Journal of Public Administration*, *80*(2), 300-323.

Li, Y.-p., Fang, L.-q., Gao, S.-q., Wang, Z., Gao, H.-w., Liu, P., Wang, Z.-r., Li, Y.-l., Zhu, X.-g., & Li, X.-l. (2013). Decision support system for the response to infectious disease emergencies based on WebGIS and mobile services in China. *PLoS One*, *8*(1), e54842.

Liberg, R. B. (2018). USING GEOGRAPHIC INFORMATION SYSTEMS IN RURAL EMERGENCY MEDICAL SERVICES: REDUCING RESPONSE TIMES BY REALLOCATING RESOURCES [Meeting Abstract]. *JOURNAL OF INVESTIGATIVE MEDICINE*, *66*(1), 104-104. <https://doi.org/10.1136/jim-2017-000663.94>

Lin, S.-Y., Beltran-Alcrudo, D., Awada, L., Hamilton-West, C., Lavarello Schettini, A., Cáceres, P., Tizzani, P., Allepuz, A., & Casal, J. (2023). Analysing WAHIS Animal Health Immediate Notifications to Understand Global Reporting Trends and Measure Early Warning Capacities (2005–2021). *Transboundary and Emerging Diseases*, *2023*, 1-10. <https://doi.org/10.1155/2023/6666672>

Litvak, M., Miller, K., Boyle, T., Bedenbaugh, R., Smith, C., Meguerdichian, D., Reisman, D., Biddinger, P., Licurse, A., & Goralnick, E. (2022). Telemedicine use in disasters: a scoping review. *Disaster medicine and public health preparedness*, *16*(2), 791-800.

Liu, J., & Guo, M. *Infectious disease monitoring and pre-warning system for use in public health emergencies, has data storage center for storing and collecting data from hospital diagnosis monitoring module, drugstore medicine sale monitoring module, and infectious disease history analysis data* CN117174332-A).

Loeb, A. E., Rao, S. S., Ficke, J. R., Morris, C. D., Riley, L. H., 3rd, & Levin, A. S. (2020). Departmental Experience and Lessons Learned With Accelerated Introduction of Telemedicine During the COVID-19 Crisis. *J Am Acad Orthop Surg*, *28*(11), e469-e476. <https://doi.org/10.5435/jaaos-d-20-00380>

Lungo, J. H. (2008). The reliability and usability of district health information software: case studies from Tanzania. *Tanzania journal of health research*, *10*(1), 39-45.

Ma, J., Huang, Y., & Zheng, Z.-J. (2020). Leveraging the Public Health Emergency Operation Center (PHEOC) for pandemic response: opportunities and challenges [Journal Article

Review]. *Global health journal (Amsterdam, Netherlands)*, *4*(4), 118-120. <https://doi.org/10.1016/j.glohj.2020.11.004>

Mahtta, D., Daher, M., Lee, M. T., Sayani, S., Shishehbor, M., & Virani, S. S. (2021). Promise and Perils of Telehealth in the Current Era. *Current Cardiology Reports*, *23*(9), 115. <https://doi.org/10.1007/s11886-021-01544-w>

Maier, N. M., & Eisner, G. R. (2017). *Method for locating internet of things network devices e.g. baby monitors, during e.g. health event, involves displaying current physical geographic location for network device for desired emergency response agencies on graphical map* US2017238129-A1

US10511950-B2).

Maleka, N. H., & Matli, W. (2022). A review of telehealth during the COVID-19 emergency situation in the public health sector: challenges and opportunities. *Journal of Science and Technology Policy Management*.

Mawudeku, A., Blench, M., Boily, L., St. John, R., Andraghetti, R., & Ruben, M. (2013). The global public health intelligence network. *Infectious disease surveillance*, 457-469.

McGregor, J., Hanlon, N., Emmons, S., Voaklander, D., & Kelly, K. (2005). If all ambulances could fly: putting provincial standards of emergency care access to the test in Northern British Columbia [Journal Article

Research Support, Non-U.S. Gov't]. *Canadian journal of rural medicine : the official journal of the Society of Rural Physicians of Canada = Journal canadien de la medecine rurale : le journal officiel de la Societe de medecine rurale du Canada*, *10*(3), 163-168.

Mykhalovskiy, E., & Weir, L. (2006). The Global Public Health Intelligence Network and early warning outbreak detection: a Canadian contribution to global public health. *Canadian journal of public health*, *97*, 42-44.

Nachiket, G., Rahul, K., Oommen, J., Supten, S., & Mark, L. (2021). Telemedicine supported strengthening of primary care in WHO South East Asia region: lessons from the COVID-19 pandemic experiences. *BMJ Innovations*, *7*(3), 580. <https://doi.org/10.1136/bmjinnov-2021-000699>

Nykiforuk, C. I. J., & Flaman, L. M. (2011). Geographic Information Systems (GIS) for Health Promotion and Public Health: A Review. *Health Promotion Practice*, *12*(1), 63-73. <http://www.jstor.org.proxy.library.upenn.edu/stable/26739088>

Odei-Lartey, E. O., Prah, R. K. D., Anane, E. A., Danwonno, H., Gyaase, S., Oppong, F. B., Afenyadu, G., & Asante, K. P. (2020). Utilization of the national cluster of district health information system for health service decision-making at the district, sub-district and community levels in selected districts of the Brong Ahafo region in Ghana. *BMC Health Services Research*, *20*(1), 514. <https://doi.org/10.1186/s12913-020-05349-5>

Odhiambo-Otieno, G. W. (2005). Evaluation of existing district health management information systems a case study of the district health systems in Kenya. *Int J Med Inform*, *74*(9), 733-744. <https://doi.org/10.1016/j.ijmedinf.2005.05.007>

Pundt, H., Spangenberg, T., & Weinkauf, R. (2010, 2010). *WEB-BASED AND CONTEXT-SENSITIVE, MOBILE GEO-TOOLS TO SUPPORT SPATIAL DECISION MAKING IN HEALTH AND EMERGENCY MANAGEMENT* [Proceedings Paper]. HEALTHINF 2010: PROCEEDINGS OF THE THIRD INTERNATIONAL CONFERENCE ON HEALTH INFORMATICS,

Rocha, C. M., Kruger, E., McGuire, S., & Tennant, M. (2013). The geographic distribution of patients seeking emergency dental care at the Royal Dental Hospital of Melbourne, Australia [Article]. *COMMUNITY DENTAL HEALTH*, *30*(3), 149-154. <https://doi.org/10.1922/CDH_3124Kruger06>

Rockwell, K. L., & Gilroy, A. S. (2020). Incorporating telemedicine as part of COVID-19 outbreak response systems. *Am J Manag Care*, *26*(4), 147-148.

Rumsfeld, J. S., Brooks, S. C., Aufderheide, T. P., Leary, M., Bradley, S. M., Nkonde-Price, C., Schwamm, L. H., Jessup, M., Ferrer, J. M. E., Merchant, R. M., Amer Heart Assoc Emergency, C., Council Cardiopulm Critical Care, P., Council Quality Care Outcomes, R., Council Cardiovasc Stroke, N., & Council Epidemiology, P. (2016). Use of Mobile Devices, Social Media, and Crowdsourcing as Digital Strategies to Improve Emergency Cardiovascular Care A Scientific Statement From the American Heart Association [Article]. *CIRCULATION*, *134*(8), E87-E108. <https://doi.org/10.1161/CIR.0000000000000428>

Schuler, F., Ma, M., & Perkins, J. (2022). *System for determining priority discrepancies between audio data and records data, has electronic computing device for generating geographical map that includes priority discrepancies, and is configured for display on display device* WO2022213023-A1

US2022318278-A1).

Shaw, N., & McGuire, S. (2017). Understanding the use of geographical information systems (GISs) in health informatics research: a review. *BMJ Health & Care Informatics*, *24*(2).

Speciale, A. M., & Freytsis, M. (2013). mHealth for Midwives: A Call to Action [Review]. *JOURNAL OF MIDWIFERY & WOMENS HEALTH*, *58*(1), 76-82. <https://doi.org/10.1111/j.1542-2011.2012.00243.x>

Steinhubl, S. R., Edwards, A. M., Waalen, J., Zambon, R., Mehta, R., Ariniello, L., Ebner, G., Baca-Motes, K., Carter, C., Felicione, E., Sarich, T., & Topol, E. (2019). HEALTHCARE RESOURCE UTILIZATION ASSOCIATED WITH ELECTROCARDIOGRAPH (ECG) SENSOR PATCH SCREENING FOR ATRIAL FIBRILLATION (AF): RESULTS FROM THE MHEALTH SCREENING TO PREVENT STROKES (MSTOPS) TRIAL [Meeting Abstract]. *JOURNAL OF THE AMERICAN COLLEGE OF CARDIOLOGY*, *73*(9), 296-296. <https://doi.org/10.1016/S0735-1097(19)30904-0>

Su, Y.-F., Wu, C.-H., & Lee, T.-F. (2017). PUBLIC HEALTH EMERGENCY RESPONSE IN TAIWAN [Article]. *HEALTH SECURITY*, *15*(2), 137-143. <https://doi.org/10.1089/hs.2016.0108>

Tamrat, T., & Kachnowski, S. (2012). Special delivery: an analysis of mHealth in maternal and newborn health programs and their outcomes around the world. *Maternal and child health journal*, *16*(5), 1092-1101.

Tao, Y., & Wu, P. *Emergency evacuation method for evacuating people urgently to ensure safety of life and property of people, based on geographic information system (GIS) in event type such as natural disaster type, involves sending emergency evacuation information to target mobile device* CN117082450-A).

Telford, S. (2020). Case Study-The Humanitarian Data Exchange: Critical Decisions, Key Results and The Road Ahead. <https://centre.humdata.org/wp-content/uploads/2020/09/hdxcasestudy.pdf>

Tounkara, K., Couacy-Hymann, E., & Diall, O. (2019). Transboundary Animal Diseases (TADs) Surveillance and Control (Including National Veterinary Services, Regional Approach, Regional and International Organisations, GF-TAD). In M. Kardjadj, A. Diallo, & R. Lancelot (Eds.), *Transboundary Animal Diseases in Sahelian Africa and Connected Regions* (pp. 53-68). Springer International Publishing. <https://doi.org/10.1007/978-3-030-25385-1_4>

Tsai, M.-K., Lee, Y.-C., Lu, C.-H., Chen, M.-H., Chou, T.-Y., & Yau, N.-J. (2012). Integrating geographical information and augmented reality techniques for mobile escape guidelines on nuclear accident sites [Article]. *JOURNAL OF ENVIRONMENTAL RADIOACTIVITY*, *109*, 36-44. <https://doi.org/10.1016/j.jenvrad.2011.12.025>

Utunen, H., Appuhamy, R., Attias, M., Ndiaye, N., George, R., Arabi, E., & Tokar, A. (2023). Observations from three years of online pandemic learning response on OpenWHO. *The International Journal of Information and Learning Technology*, *40*(5), 527-540.

Utunen, H., Tokar, A., Dancante, M., & Piroux, C. (2023). Online learning for WHO priority diseases with pandemic potential: evidence from existing courses and preparing for Disease X. *Archives of Public Health*, *81*(1), 61. <https://doi.org/10.1186/s13690-023-01080-9>

Vahidi, H., Taleai, M., Yan, W., & Shaw, R. (2021). Digital Citizen Science for Responding to COVID-19 Crisis: Experiences from Iran [Article]. *INTERNATIONAL JOURNAL OF ENVIRONMENTAL RESEARCH AND PUBLIC HEALTH*, *18*(18), Article 9666. <https://doi.org/10.3390/ijerph18189666>

Valentin, S., Boudoua, B., Sewalk, K., Arınık, N., Roche, M., Lancelot, R., & Arsevska, E. (2023). Dissemination of information in event-based surveillance, a case study of Avian Influenza. *PLOS ONE*, *18*(9), e0285341. <https://doi.org/10.1371/journal.pone.0285341>

Vardell, E. (2020). Global Health Observatory Data Repository. *Med Ref Serv Q*, *39*(1), 67-74. <https://doi.org/10.1080/02763869.2019.1693231>

Wang, Z., Li, H., Song, J., Gong, X., Chen, N., Song, C., & Lu, Y. *Public health event emergency medical facility addressing method based on point of interest data, involves establishing a file geographic information database by using a file geographic information system software* CN112232599-A).

Wangdi, K., Sarma, H., Leaburi, J., McBryde, E., & Clements, A. C. A. (2020). Evaluation of the malaria reporting system supported by the District Health Information System 2 in Solomon Islands. *Malaria Journal*, *19*(1), 372. <https://doi.org/10.1186/s12936-020-03442-y>

Wright, S., Spaulding, R., & Henley, W. (2022). A Multipronged Digital Response to Increased Demand for Telehealth Support and Training During the COVID-19 Pandemic [Case Reports]. *Journal of technology in behavioral science*, *7*(1), 73-80. <https://doi.org/10.1007/s41347-021-00224-4>

Young, M. M., Dubeau, C., & Corazza, O. (2015). Detecting a signal in the noise: monitoring the global spread of novel psychoactive substances using media and other open-source information [Article]. *HUMAN PSYCHOPHARMACOLOGY-CLINICAL AND EXPERIMENTAL*, *30*(4), 319-326. <https://doi.org/10.1002/hup.2477>

Yu, Z., & Liu, P. *Method for locating geographical position of graph convolution network model based on attention aggregate, involves inputting result obtained by training graph convolution network model to geographic position predictor of multilayer sensor* CN116166865-A).

Zhen, W., Li, C., Chen, R., Wei, Z., Chen, T., & Yan, J. *Multi-scene city emergency sampling site addressing optimization method for city public health safety technical field, involves obtaining mobile phone signaling data of target city, interest point, and basic geographic information data* CN115860213-A).
