# Supplementary material for: Knowledge attributes of public health management information systems used in health emergencies: a scoping review
Source: Front Public Health. 2025 Mar 20;12:1458867. doi: 10.3389/fpubh.2024.1458867 (PMC11969037; doi:10.3389/fpubh.2024.1458867)
Supplement: SUPPLEMENTARY DATA SHEET 3 — Supplementary Tables C1 to C9. [file Data_Sheet_3.zip › SupplementaryTables_C1_C9_KnowledgeAttributesPerHMIS/SupplementaryTable_C9_UserFriendly.docx]

**Supplementary table C9: Literary sources for knowledge attributes of HMIS reviewed in the study – User-friendliness.**

| **IMS** | **Highly user-friendly** | **Moderately user-friendly** |
| --- | --- | --- |
| TACIT Knowledge containing IMS | | |
| GPHIN | (Carter et al., 2020; Keller et al., 2009; Roberts & Elbe, 2017; Young et al., 2015) |  |
| GLEWS | (Lin et al., 2023) |  |
| HealthMap | (Brownstein et al., 2008) |  |
| OpenWHO | (Attias et al., 2022; Bonkoungou et al., 2023; George et al., 2022; Utunen, 2021; Utunen, Appuhamy, et al., 2023; Utunen, George, et al., 2021; Utunen et al., 2022; Utunen, Tokar, et al., 2023; Utunen, Van Kerkhove, et al., 2021) |  |
| ProMED |  | (Chuang et al., 2022; Stewart & Denecke, 2010; Zeldenrust et al., 2008) |
| Telemedicine | (Doraiswamy et al., 2020) |  |
| mHealth | (Bhattacharya et al., 2018; EŞİYOK et al., 2023; Shahriar A & Pradeep R, 2010) |  |
| EXPLICIT Knowledge containing IMS | | |
| COVID-19 | (Irwansyah et al., 2020; Sulaiman et al., 2020) |  |
| EOC |  | (Chan et al., 2016; Shojaei et al., 2023) |
| HDX | (Telford, 2020) |  |
| DHIS | (Asaduzzaman et al., 2024; Ndlovu et al., 2022; Thangasamy et al., 2016) |  |
| GIS | (Kaiser et al., 2003) |  |
| GHO |  | (Vardell, 2020; Zaveri et al., 2013) |

Asaduzzaman, M., Mekonnen, Z., Rodland, E. K., Sahay, S., Winkler, A. S., & Gradmann, C. (2024). District health information system (DHIS2) as integrated antimicrobial resistance surveillance platform: An exploratory qualitative investigation of the one health stakeholders' viewpoints in Ethiopia [Article

Early Access]. *INTERNATIONAL JOURNAL OF MEDICAL INFORMATICS*, *181*, Article 105268. <https://doi.org/10.1016/j.ijmedinf.2023.105268>

Attias, M., Utunen, H., Ndiaye, N., & Mattar, L. (2022). Open-Access Learning as a Pathway to Equity During Health Emergencies. The Learning Ideas Conference,

Bhattacharya, S., Kumar, A., Kaushal, V., & Singh, A. (2018). Applications of m-Health and e-Health in Public Health Sector: the challenges and opportunities. *International Journal of medicine and public Health*, *8*(2).

Bonkoungou, B., Utunen, H., Talisuna, A. O., O'Connell, G., Koua, E., Chamla, D. D., Arabi, E., Tokar, A., & Gueye, A. S. (2023). Online capacity building for the health workforce: the case of the Integrated Disease Surveillance and Response for the African region [Article]. *JOURNAL OF PUBLIC HEALTH IN AFRICA*, *14*(12), Article 2478. <https://doi.org/10.4081/jphia.2023.2478>

Brownstein, J. S., Freifeld, C. C., Reis, B. Y., & Mandl, K. D. (2008). Surveillance Sans Frontières: Internet-Based Emerging Infectious Disease Intelligence and the HealthMap Project. *PLOS Medicine*, *5*(7), e151. <https://doi.org/10.1371/journal.pmed.0050151>

Carter, D., Stojanovic, M., Hachey, P., Fournier, K., Rodier, S., Wang, Y., & de Bruijn, B. (2020, 2020). *Global Public Health Surveillance Using Media Reports: Redesigning GPHIN* [Proceedings Paper]. DIGITAL PERSONALIZED HEALTH AND MEDICINE,

Chan, E., Anslow, C., Seyed, T., & Maurer, F. (2016). Envisioning the emergency operations centre of the future. *Collaboration Meets Interactive Spaces*, 349-372.

Chuang, T., Chiu, Y., & Chang, Y. (2022). Linguistic Pattern-infused Dual-channel BiLSTM with Attention to Generate Dengue Case Summaries from ProMED-mail database. *International Journal of Infectious Diseases*, *116*, S98-S99.

Doraiswamy, S., Abraham, A., Mamtani, R., & Cheema, S. (2020). *Use of telemedicine/ telehealth for geriatric care during the COVID-19 pandemic - A scoping review and evidence mapping*. <https://doi.org/10.17605/OSF.IO/26Z74>

EŞİYOK, A., DİVANOĞLU, S. U., & ÇELİK, R. (2023). Digitalization in Healthcare-Mobile Health (M-Health) Applications. *Aksaray Üniversitesi İktisadi ve İdari Bilimler Fakültesi Dergisi*, *15*(2), 165-174.

George, R., Utunen, H., Ndiaye, N., Tokar, A., Mattar, L., Piroux, C., & Gamhewage, G. (2022). Ensuring equity in access to online courses: Perspectives from the WHO health emergency learning response. *World Medical & Health Policy*, *14*(2), 413-427.

Irwansyah, E., Budiharto, W., Widhyatmoko, D., Istamar, A., & Panghurian, F. P. (2020). Monitoring Coronavirus COVID-19/SARS-CoV-2 Pandemic using GIS Dashboard: International and Indonesia Context. *Preprints* <https://doi.org/10.20944/preprints202008.0415.v1> I

Kaiser, R., Spiegel, P. B., Henderson, A. K., & Gerber, M. L. (2003). The application of geographic information systems and global positioning systems in humanitarian emergencies: lessons learned, programme implications and future research. *Disasters*, *27*(2), 127-140.

Keller, M., Blench, M., Tolentino, H., Freifeld, C. C., Mandl, K. D., Mawudeku, A., Eysenbach, G., & Brownstein, J. S. (2009). Use of Unstructured Event-Based Reports for Global Infectious Disease Surveillance [Article]. *EMERGING INFECTIOUS DISEASES*, *15*(5), 689-695. <https://doi.org/10.3201/eid1505.081114>

Lin, S.-Y., Beltran-Alcrudo, D., Awada, L., Hamilton-West, C., Lavarello Schettini, A., Cáceres, P., Tizzani, P., Allepuz, A., & Casal, J. (2023). Analysing WAHIS Animal Health Immediate Notifications to Understand Global Reporting Trends and Measure Early Warning Capacities (2005–2021). *Transboundary and Emerging Diseases*, *2023*, 1-10. <https://doi.org/10.1155/2023/6666672>

Ndlovu, K., Mauco, K. L., Keetile, M., Kadimo, K., Senyatso, R. Y., Ntebela, D., Valela, B., & Murambi, C. (2022). Acceptance of the District Health Information System Version 2 Platform for Malaria Case-Based Surveillance By Health Care Workers in Botswana: Web-Based Survey [Article]. *JMIR FORMATIVE RESEARCH*, *6*(3), Article e32722. <https://doi.org/10.2196/32722>

Roberts, S. L., & Elbe, S. (2017). Catching the flu: Syndromic surveillance, algorithmic governmentality and global health security [Article]. *SECURITY DIALOGUE*, *48*(1), 46-62. <https://doi.org/10.1177/0967010616666443>

Shahriar A, & Pradeep R. (2010). mHealth-an ultimate platform to serve the unserved. *Yearbook of medical informatics*, *19*(01), 94-100.

Shojaei, F., Qaraeian, P., Firoozbakht, A., Chhabra, D., & Jahangiri, K. (2023). The necessity for an integrated Emergency Operations Center (EOC) among first responders: Lesson learned from two Iranian railway accidents. *Heliyon*, *9*(5).

Stewart, A., & Denecke, K. (2010). Using ProMED-Mail and MedWorm Blogs for Cross-Domain Pattern Analysis in Epidemic Intelligence. In *MEDINFO 2010* (pp. 437-441). IOS Press.

Sulaiman, N., Abid, S. K., Chan, S. W., Nazir, U., Mahmud, N. P. N., Latib, S., Hafidz, H., Shahlal, S., Sapuan, S., & Fernando, T. (2020). Geospatial dashboards for mapping and tracking of novel coronavirus pandemic. Proc. Int. Conf. Ind. Eng. Oper. Manag,

Telford, S. (2020). Case Study-The Humanitarian Data Exchange: Critical Decisions, Key Results and The Road Ahead. <https://centre.humdata.org/wp-content/uploads/2020/09/hdxcasestudy.pdf>

Thangasamy, P., Gebremichael, M., Kebede, M., Sileshi, M., Elias, N., & Tesfaye, B. (2016). A pilot study on district health information software 2: challenges and lessons learned in a developing country: an experience from Ethiopia. *Int Res J Eng Technol*, *3*(5), 1646-1651.

Utunen, H. (2021). Transferring real-time knowledge free of charge through WHO’s online learning platform OpenWHO. org. *QScience Proceedings*, *2022*(1), 5.

Utunen, H., Appuhamy, R., Attias, M., Ndiaye, N., George, R., Arabi, E., & Tokar, A. (2023). Observations from three years of online pandemic learning response on OpenWHO. *The International Journal of Information and Learning Technology*, *40*(5), 527-540.

Utunen, H., George, R., Ndiaye, N., Tokar, A., Attias, M., & Gamhewage, G. (2021). Delivering WHO’s life-saving information in real-time during a pandemic through an online learning platform: evidence from global use. In *Public Health and Informatics* (pp. 969-973). IOS Press.

Utunen, H., Ndiaye, N., Attias, M., Mattar, L., Tokar, A., & Gamhewage, G. (2022). Multilingual Approach to COVID-19 Online Learning Response on OpenWHO. org. *Informatics and Technology in Clinical Care and Public Health*, *289*, 192.

Utunen, H., Tokar, A., Dancante, M., & Piroux, C. (2023). Online learning for WHO priority diseases with pandemic potential: evidence from existing courses and preparing for Disease X. *Archives of Public Health*, *81*(1), 61. <https://doi.org/10.1186/s13690-023-01080-9>

Utunen, H., Van Kerkhove, M. D., Tokar, A., O'Connell, G., Gamhewage, G. M., & Fall, I. S. (2021). One year of pandemic learning response: benefits of massive online delivery of the World Health Organization’s technical guidance. *JMIR Public Health and Surveillance*, *7*(4), e28945.

Vardell, E. (2020). Global health observatory data repository. *Medical reference services quarterly*, *39*(1), 67-74.

Young, M. M., Dubeau, C., & Corazza, O. (2015). Detecting a signal in the noise: monitoring the global spread of novel psychoactive substances using media and other open-source information [Article]. *HUMAN PSYCHOPHARMACOLOGY-CLINICAL AND EXPERIMENTAL*, *30*(4), 319-326. <https://doi.org/10.1002/hup.2477>

Zaveri, A., Lehmann, J., Auer, S., Hassan, M. M., Sherif, M. A., & Martin, M. (2013). Publishing and interlinking the global health observatory dataset. *Semantic Web*, *4*(3), 315-322.

Zeldenrust, M., Rahamat-Langendoen, J., Postma, M., & Van Vliet, J. (2008). The value of ProMED-mail for the Early Warning Committee in the Netherlands: more specific approach recommended. *Eurosurveillance*, *13*(6), 7-8.
