# Supplementary material for: Knowledge attributes of public health management information systems used in health emergencies: a scoping review
Source: Front Public Health. 2025 Mar 20;12:1458867. doi: 10.3389/fpubh.2024.1458867 (PMC11969037; doi:10.3389/fpubh.2024.1458867)
Supplement: SUPPLEMENTARY DATA SHEET 4 — Supplementary Tables D1 to D13. [file Data_Sheet_4.zip › SupplementaryTables_D1_D13_SettingsPerHMIS/SupplementaryTable_D10_ProMED.docx]

**Supplementary Table D10: Countries where ProMED has been used.**

| **Author** | **Year of publication** | **Countries** |
| --- | --- | --- |
| Al-Tawfiq,(1) | 2014 | Global with a mention of SARS in China in 2002 |
| Aslanov et al(2) | 2017 | United Kingdom, as well as the recent outbreaks of avian influenza in Europe, Southeast Asia, and the United States, Vietnam, Hong Kong, Germany, Canada |
| Babalobi & Cowen (3) | 2005 | Nigeria |
| Bijkerk et al (4) | 2017 | Netherlands |
| Breit (5) | 2016 | Global |
| Burki (6) | 2023 | China, Russia, Saudi Arabia, French Guinea, Nigeria, Iraq, Jordan, France, Scotland, America |
| Carrion & Madoff (7) | 2017 | Global |
| Chang (8) | 2022 | Southeast Asia |
| Chuang et al (9) | 2022 | na |
| Cowen et al (10) | 2006 | Global |
| Hugh-Jones (11) | 2001 | Global |
| Madoff & Woodall(12) | 2005 | Fiji, Sweden, Australia (3), USA (3), Russia (2), Costa Rica, Norway, Netherlands, Philippines, Malaysia (2), Chile, Switzerland, Japan, Africa, DRC, India (2), Sierra Leone, Nepal, Madagascar, Israel, Turkey, Singapore, Bolivia, El Salvador, Lesotho, Peru, Pakistan, China, Gabon, S. Africa (2), Brazile (4), |
| Mitchell (13) | 1997 | Na |
| Pollack et al (14) | 2013 | Saudi Arabia, Jordan, UK, Netherlands, Qatar |
| Rolland et al (15) | 2020 | Global |
| Stewart & Denecke (16) | 2010 | na |
| Woodall & Callisher (17) | 2001 | China, Gabon, |
| Woodall (18) | 2001 | Global |
| Yan (19) | 2017 | Not stated |
| Yuill (20) | 2013 | Sudan |
| You et al (21) | 2021 | Americas (Zika), West Africa (Ebola) |
| Yu & Madoff(22) | 2024 | Hong Kong, Germany, Canada, Vietnam |
| Zeldenrust et al (23) | 2008 | Netherlands |

**References**

1. Al-Tawfiq JA, Zumla A, Gautret P, Gray GC, Hui DS, Al-Rabeeah AA, et al. Emerging respiratory tract infections 1 Surveillance for emerging respiratory viruses. LANCET INFECTIOUS DISEASES. 2014;14(10):992-1000.

2. Aslanov B, Pshenichnaya N, Melnik V, Rakhmanova N. Promed-mail: internet-based surveillance system for emerging infectious diseases. Профилактическая и клиническая медицина. 2017(2):54-9.

3. Babalobi O, Cowen P. PROMED–mail: an electronic mail disease-reporting: a case study. 2005.

4. Bijkerk P, Monnier AA, Fanoy EB, Kardamanidis K, Friesema IH, Knol MJ. ECDC Round Table Report and ProMed-mail most useful international information sources for the Netherlands Early Warning Committee. Eurosurveillance. 2017;22(14):30502.

5. Breit NA, Allen T, Arnold B, Huff A, Madoff L, Pollack M. Evaluation of ProMED-mail global surveillance capability. INTERNATIONAL JOURNAL OF INFECTIOUS DISEASES. 2016;53:140-.

6. Burki T. "Unfettered flow": how ProMED-mail keeps the world alert. LANCET. 2023;401(10373):259-60.

7. Carrion M, Madoff LC. ProMED-mail: 22 years of digital surveillance of emerging infectious diseases. International Health. 2017;9(3):177-83.

8. Chang YC, Chiu YW, Chuang TW. Linguistic Pattern-Infused Dual-Channel Bidirectional Long Short-term Memory With Attention for Dengue Case Summary Generation From the Program for Monitoring Emerging Diseases-Mail Database: Algorithm Development Study. JMIR PUBLIC HEALTH AND SURVEILLANCE. 2022;8(7).

9. Chuang T, Chiu Y, Chang Y. Linguistic Pattern-infused Dual-channel BiLSTM with Attention to Generate Dengue Case Summaries from ProMED-mail database. International Journal of Infectious Diseases. 2022;116:S98-S9.

10. Cowen P, Garland T, Hugh-Jones ME, Shimshony A, Handysides S, Kaye D, et al. Evaluation of ProMED-mail as an electronic early warning system for emerging animal diseases: 1996 to 2004. Journal of the American Veterinary Medical Association. 2006;229(7):1090-9.

11. Hugh-Jones M. Global awareness of disease outbreaks: the experience of ProMED-mail. Public Health Reports. 2001;116(Suppl 2):27.

12. Madoff LC, Woodall JP. The Internet and the Global Monitoring of Emerging Diseases: Lessons from the First 10 Years of ProMED-mail. Archives of Medical Research. 2005;36(6):724-30.

13. Mitchell P. ProMED-mail: outbreak intelligence or rash reporting? LANCET. 1997;350(9091):1610-.

14. Pollack MP, Pringle C, Madoff LC, Memish ZA. Latest outbreak news from ProMED-mail: novel coronavirus–Middle East. International Journal of Infectious Diseases. 2013;17(2):e143-e4.

15. Rolland C, Lazarus C, Giese C, Monate B, Travert AS, Salomon J. Early Detection of Public Health Emergencies of International Concern through Undiagnosed Disease Reports in ProMED-Mail. Emerg Infect Dis. 2020;26(2):336-9.

16. Stewart A, Denecke K. Using ProMED-Mail and MedWorm Blogs for Cross-Domain Pattern Analysis in Epidemic Intelligence. MEDINFO 2010: IOS Press; 2010. p. 437-41.

17. Woodall J, Calisher CH. ProMED-mail: background and purpose. Emerging infectious diseases. 2001;7(3 Suppl):563.

18. Woodall JP. Global surveillance of emerging diseases: the ProMED-mail perspective. Cadernos de saude publica. 2001;17:S147-S54.

19. Yan SJ, Chughtai AA, Macintyre CR. Utility and potential of rapid epidemic intelligence from internet-based sources. INTERNATIONAL JOURNAL OF INFECTIOUS DISEASES. 2017;63:77-87.

20. Yuill TM, Woodall JP, Baekeland S. Latest outbreak news from ProMED-mail. Yellow fever outbreak-Darfur Sudan and Chad. INTERNATIONAL JOURNAL OF INFECTIOUS DISEASES. 2013;17(7):E476-E8.

21. You J, Expert P, Costelloe C. Using text mining to track outbreak trends in global surveillance of emerging diseases: ProMED-mail. Journal of the Royal Statistical Society Series A: Statistics in Society. 2021;184(4):1245-59.

22. Yu VL, Madoff LC. ProMED-mail: an early warning system for emerging diseases. Clinical infectious diseases. 2004;39(2):227-32.

23. Zeldenrust M, Rahamat-Langendoen J, Postma M, Van Vliet J. The value of ProMED-mail for the Early Warning Committee in the Netherlands: more specific approach recommended. Eurosurveillance. 2008;13(6):7-8.
