# Supplementary material for: Knowledge attributes of public health management information systems used in health emergencies: a scoping review
Source: Front Public Health. 2025 Mar 20;12:1458867. doi: 10.3389/fpubh.2024.1458867 (PMC11969037; doi:10.3389/fpubh.2024.1458867)
Supplement: SUPPLEMENTARY DATA SHEET 4 — Supplementary Tables D1 to D13. [file Data_Sheet_4.zip › SupplementaryTables_D1_D13_SettingsPerHMIS/SupplementaryTable_D11_Telemed.docx]

**Supplementary Table D11: Countries where Telemedicine platforms have been used.**

| **Author** | **Year of publication** | **Countries** |
| --- | --- | --- |
| Bashshur (1) | 2001 | USA |
| Bashshur (2) | 2001 | USA, Malaysia, Australia, South Africa |
| Bokolo (3) | 2023 | Not stated |
| Bokolo (4) | 2021 | USA, China, Taiwan, South Korea, Israel, Australia |
| Brown (5) | 2008 | USA |
| Doarn (6) | 2019 | USA, Austria |
| Doraiswamy (7) | 2020 | USA, Canada, Italy, Spain, UK |
| Greiwe(8) | 2022 | USA |
| Hosseinzadeh(9) | 2023 | Bangladesh |
| Khan (10) | 2002 | Alaska, USA |
| Kilova & Uzunova (11) | 2020 | Not stated |
| Leite & Hodgkinson (12) | 2021 | Not stated |
| Litvak et al (13) | 2022 | Not stated |
| Loeb et al (14) | 2020 | USA |
| Mahtta et al (15) | 2021 | Not stated |
| Maleka & Matli (16) | 2022 | Not stated |
| Nachiket et al (17) | 2021 | Bhutan, Sri Lanka, Indonesia, India, Myanmar, Nepal |
| Pierce & Stevermer (18) | 2020 | USA |
| Rockwell & Gilroy (19) | 2020 | USA, Europe |
| Shanbehzadeh et al (20) | 2021 | UK, USA, Germany, China, Spain, Denmark, India, France, Brazil |
| Song et al (21) | 2020 | China |
| Wang et al (22) | 2020 | China |
| Wright (23) | 2020 | USA |
| Ye (24) | 2020 | Not stated |

**References**

1. Bashshur RL. Where we are in telemedicine/telehealth, and where we go from here. TELEMEDICINE JOURNAL AND E-HEALTH. 2001;7(4):273-7.

2. Bashshur RL, Mandil SH, Shannon GW. Executive summary. TELEMEDICINE JOURNAL AND E-HEALTH. 2002;8(1):95-107.

3. Bokolo AJ. Application of telemedicine and eHealth technology for clinical services in response to COVID‑19 pandemic. Health and technology. 2021;11(2):359-66.

4. Bokolo AJ. Investigating the implementation of telehealth and digital technologies during public health crisis: A qualitative review. The International Journal of Health Planning and Management. 2023;38(5):1212-27.

5. Brown EV. Robotic assistance remedy. The Michigan Stroke Network utilizes remote presence robots to bring needed specialists to stroke patients at remote hospitals. Health management technology. 2008;29(7):18-21.

6. Doarn CR, Merrell RC. Telemedicine, Telehealth, and the Public Health Good. TELEMEDICINE AND E-HEALTH. 2019;25(9):773-4.

7. Doraiswamy S, Abraham A, Mamtani R, Cheema S. Use of telemedicine/ telehealth for geriatric care during the COVID-19 pandemic - A scoping review and evidence mapping. Open Science Framework2020.

8. Greiwe J. Telemedicine Lessons Learned During the COVID-19 Pandemic. Current Allergy and Asthma Reports. 2022;22(1):1-5.

9. Hosseinzadeh H, Ratan ZA, Nahar K, Dadich A, Al-Mamun A, Ali S, et al. Telemedicine Use and the Perceived Risk of COVID-19: Patient Experience. Int J Environ Res Public Health. 2023;20(4).

10. Khan B, Hiratsuka VY, Dillard D, Robinson R, Mau M. Availability and Deployment of Telemedicine/Telehealth Technologies in Rural Alaska. Federal practitioner : for the health care professionals of the VA, DoD, and PHS. 2012;29(12):19-21.

11. Kilova K, Uzunova S. Telemedicine in assistance to healthcare in the COVID-19 pandemic. Acta Medica Bulgarica. 2020;47(4):63-8.

12. Leite H, Hodgkinson IR. Telemedicine co‐design and value co‐creation in public health care. Australian Journal of Public Administration. 2021;80(2):300-23.

13. Litvak M, Miller K, Boyle T, Bedenbaugh R, Smith C, Meguerdichian D, et al. Telemedicine use in disasters: a scoping review. Disaster medicine and public health preparedness. 2022;16(2):791-800.

14. Loeb AE, Rao SS, Ficke JR, Morris CD, Riley LH, 3rd, Levin AS. Departmental Experience and Lessons Learned With Accelerated Introduction of Telemedicine During the COVID-19 Crisis. J Am Acad Orthop Surg. 2020;28(11):e469-e76.

15. Mahtta D, Daher M, Lee MT, Sayani S, Shishehbor M, Virani SS. Promise and Perils of Telehealth in the Current Era. Current Cardiology Reports. 2021;23(9):115.

16. Maleka NH, Matli W. A review of telehealth during the COVID-19 emergency situation in the public health sector: challenges and opportunities. Journal of Science and Technology Policy Management. 2022.

17. Nachiket G, Rahul K, Oommen J, Supten S, Mark L. Telemedicine supported strengthening of primary care in WHO South East Asia region: lessons from the COVID-19 pandemic experiences. BMJ Innovations. 2021;7(3):580.

18. Pierce RP, Stevermer JJ. Disparities in the use of telehealth at the onset of the COVID-19 public health emergency. Journal of Telemedicine and Telecare. 2020;29(1):3-9.

19. Rockwell KL, Gilroy AS. Incorporating telemedicine as part of COVID-19 outbreak response systems. Am J Manag Care. 2020;26(4):147-8.

20. Shanbehzadeh M, Kazemi-Arpanahi H, Kalkhajeh SG, Basati G. Systematic review on telemedicine platforms in lockdown periods: Lessons learned from the COVID-19 pandemic. Journal of Education and Health Promotion. 2021;10.

21. Song X, Liu X, Wang C. The role of telemedicine during the COVID-19 epidemic in China—experience from Shandong province. Critical Care. 2020;24(1):178.

22. Wang Y, Li B, Liu L. Telemedicine experience in China: our response to the pandemic and current challenges. Frontiers in Public Health. 2020;8:549669.

23. Wright S, Spaulding R, Henley W. A Multipronged Digital Response to Increased Demand for Telehealth Support and Training During the COVID-19 Pandemic. Journal of technology in behavioral science. 2022;7(1):73-80.

24. Ye J. The role of health technology and informatics in a global public health emergency: practices and implications from the COVID-19 pandemic. JMIR medical informatics. 2020;8(7):e19866.
