# Supplementary material for: Knowledge attributes of public health management information systems used in health emergencies: a scoping review
Source: Front Public Health. 2025 Mar 20;12:1458867. doi: 10.3389/fpubh.2024.1458867 (PMC11969037; doi:10.3389/fpubh.2024.1458867)
Supplement: SUPPLEMENTARY DATA SHEET 4 — Supplementary Tables D1 to D13. [file Data_Sheet_4.zip › SupplementaryTables_D1_D13_SettingsPerHMIS/SupplementaryTable_D13_COVID19.docx]

**Supplementary Table D13: Countries where WHO COVID-19 dashboard has been used.**

| **Author** | **Year of publication** | **Countries** |
| --- | --- | --- |
| Ahmed et al (1) | 2020 | African region |
| Maya et al (2) | 2022 | Global |
| Iwansyah et al(3) | 2020 | Indonesia |
| Ivanković et al (4) | 2021 | Global |
| Sulaiman et al (5) | 2020 | Not stated |
| WHO (6) | 2023 | na |

**References**

1. Ahmed K, Bukhari MA, Mlanda T, Kimenyi JP, Wallace P, Lukoya CO, et al. Novel approach to support rapid data collection, management, and visualization during the COVID-19 outbreak response in the world health organization African region: development of a data summarization and visualization tool. JMIR Public Health and Surveillance. 2020;6(4):e20355.

2. Allan M, Lièvre M, Laurenson-Schafer H, de Barros S, Jinnai Y, Andrews S, et al. The World Health Organization COVID-19 surveillance database. International journal for equity in health. 2022;21(Suppl 3):167.

3. Irwansyah E, Budiharto W, Widhyatmoko D, Istamar A, Panghurian FP. Monitoring Coronavirus COVID-19/SARS-CoV-2 Pandemic using GIS Dashboard: International and Indonesia Context. Preprints 2020.

4. Ivanković D, Barbazza E, Bos V, Brito Fernandes Ó, Jamieson Gilmore K, Jansen T, et al. Features constituting actionable COVID-19 dashboards: descriptive assessment and expert appraisal of 158 public web-based COVID-19 dashboards. Journal of medical Internet research. 2021;23(2):e25682.

5. Sulaiman N, Abid SK, Chan SW, Nazir U, Mahmud NPN, Latib S, et al., editors. Geospatial dashboards for mapping and tracking of novel coronavirus pandemic. Proc Int Conf Ind Eng Oper Manag; 2020.

6. World Health Organization. WHO COVID-19 dashboard2023, . Available from: <https://data.who.int/dashboards/covid19/about?n=c>.
