# Supplementary material for: Knowledge attributes of public health management information systems used in health emergencies: a scoping review
Source: Front Public Health. 2025 Mar 20;12:1458867. doi: 10.3389/fpubh.2024.1458867 (PMC11969037; doi:10.3389/fpubh.2024.1458867)
Supplement: SUPPLEMENTARY DATA SHEET 4 — Supplementary Tables D1 to D13. [file Data_Sheet_4.zip › SupplementaryTables_D1_D13_SettingsPerHMIS/SupplementaryTable_D3_GIS.docx]

**Supplementary Table D3: Countries where GIS has been used.**

| **Author** | **Year of publication** | **Countries** |
| --- | --- | --- |
| Ahmadi et al (1) | 2017 | Iran |
| Boulos et al (2) | 2017 | na |
| Davenhall & Kinabrew(3) | 2012 | Global |
| Du et al (4) | nd | na |
| Ebener et al (5) | 2019 | LIC and MIC |
| Ferguson et al (6) | 2012 | Vietnam, EMRO region, USA, Indonesia, Singapore, UAE, Greece, Italy, China, Africa, |
| Fradelos et al (7) | 2014 | na |
| Fuad et al (8) | 2006 | Indonesia |
| Gülden et al (9) | 2004 | Turkey |
| Jin et al (10) | nd | Sierra Leone, Iraq, Ethiopia, Guinea, DRC, East Timor, Europe |
| Kaiser et al (11) | 2003 | Poland |
| Kisiala et al (12) | 2022 | na |
| Li et al (13) | 2013 | China |
| Liberg (14) | 2018 | Not stated |
| Liu & Guo (15) | nd | Not stated |
| Maier & Eisner (16) | 2020 | Not sated |
| McGregor et al (17) | 2005 | Canada |
| Melnick (18) | 2002 | na |
| Nykiforuk & Flaman (19) | 2011 | na |
| Pundt et al (20) | 2010 | Germany |
| Rocha et al (21) | 2013 | Australia |
| Schuler et al (22) | 2022 | Not stated |
| Shaw & Guire (23) | 2017 | Japan, Australia, USA, Belgium, |
| Tanser & Sueur(24) | 2002 | Africa |
| Tao & Wu (25) | nd | na |
| Tomaszewski et al (26) | 2015 | USA, Mexico, Indonesia, Sri Lanka, India, Maldives, Thailand, E.Africa, Haiti, Kenya, Libya, Syria, Philippines |
| Tsai et al (27) | 2012 | Not stated |
| Tzavella et al (28) | 2018 | Brazil, India, China, |
| Wang et al (29) | nd | Not stated |
| Waring et al (30) | 2005 | USA |
| Yu & Liu (31) | nd | Not stated |
| Zhen et al (32) | 2010 | China, India, Brazil |
| Zhen et al (33) | nd | Na |

**References**

1. Ahmadi M, Valinejadi A, Goodarzi A, Safari A, Hemmat M, Majdabadi HA, et al. Geographic Information System (GIS) capabilities in traffic accident information management: a qualitative approach. Electron Physician. 2017;9(6):4533-40.

2. Boulos MNK, Lu Z, Guerrero P, Jennett C, Steed A. From urban planning and emergency training to Pokemon Go: applications of virtual reality GIS (VRGIS) and augmented reality GIS (ARGIS) in personal, public and environmental health. INTERNATIONAL JOURNAL OF HEALTH GEOGRAPHICS. 2017;16.

3. Davenhall WF, Kinabrew C. GIS in health and human services. Springer handbook of geographic information. 2012:557-78.

4. Du J, Du X, Mao J, Meng H, Shi H, Sun J, et al., inventorsNuclear, chemical and biological medical rescue expert consultation information system, has geographic information sub-system equipped with object rapid positioning unit, harm evaluation unit and geographical information system databases patent CN101661531-A CN101661531-B.

5. Ebener S, Stenberg K, Brun M, Monet J-P, Ray N, Lawrence Sobel H, et al. Proposing standardised geographical indicators of physical access to emergency obstetric and newborn care in low-income and middle-income countries. BMJ GLOBAL HEALTH. 2019;4.

6. Ferguson WJ, Louie RF, Tang CS, Vy JH, Wallace AP, Peng LS, et al. Geographic Information Systems Can Enhance Crisis Standards of Care During Complex Emergencies and Disasters: A Strategy for Global Positioning System–Tracked, H: 2: Fuel Cell–Powered, and Knowledge-Optimized Point-of-Care Medical Intelligence. Point of Care. 2012;11(4):184-90.

7. Fradelos EC, Papathanasiou IV, Mitsi D, Tsaras K, Kleisiaris CF, Kourkouta L. Health based geographic information systems (GIS) and their applications. Acta Informatica Medica. 2014;22(6):402.

8. Fuad A, Kusnanto H, Utarini A, Dijk JV, Groothoff J. The use of geographic information systems (GIS) for rapid assessment of health facilities following a disaster: the case of the tsunami disaster in the province of Aceh. APAMI 2006. 2006.

9. Gülden B, Mumcuoglu E, Baykal N. A GIS system for ambulatory transportation. Proceedings of the Second IASTED International Conference on Biomedical Engineering; 20042004. p. 431-5.

10. Jin L, Li Q, Niu Y, inventorsEmergent public health event processing and displaying method, involves determining public sanitary event corresponding to burst response level, and determining burst public health event response level by electronic map display patent CN110084730-A.

11. Kaiser R, Spiegel PB, Henderson AK, Gerber ML. The application of geographic information systems and global positioning systems in humanitarian emergencies: lessons learned, programme implications and future research. Disasters. 2003;27(2):127-40.

12. Kisiala W, Racka I, Suszynska K. Population Access to Hospital Emergency Departments: The Spatial Analysis in Public Health Research. INTERNATIONAL JOURNAL OF ENVIRONMENTAL RESEARCH AND PUBLIC HEALTH. 2022;19(3).

13. Li Y-p, Fang L-q, Gao S-q, Wang Z, Gao H-w, Liu P, et al. Decision support system for the response to infectious disease emergencies based on WebGIS and mobile services in China. PLoS One. 2013;8(1):e54842.

14. Liberg RB. USING GEOGRAPHIC INFORMATION SYSTEMS IN RURAL EMERGENCY MEDICAL SERVICES: REDUCING RESPONSE TIMES BY REALLOCATING RESOURCES. JOURNAL OF INVESTIGATIVE MEDICINE. 2018;66(1):104-.

15. Liu J, Guo M, inventorsInfectious disease monitoring and pre-warning system for use in public health emergencies, has data storage center for storing and collecting data from hospital diagnosis monitoring module, drugstore medicine sale monitoring module, and infectious disease history analysis data patent CN117174332-A.

16. Maier NM, Eisner GR, inventorsMethod for locating internet of things network devices e.g. baby monitors, during e.g. health event, involves displaying current physical geographic location for network device for desired emergency response agencies on graphical map patent US2017238129-A1

US10511950-B2. 2017.

17. McGregor J, Hanlon N, Emmons S, Voaklander D, Kelly K. If all ambulances could fly: putting provincial standards of emergency care access to the test in Northern British Columbia. Canadian journal of rural medicine : the official journal of the Society of Rural Physicians of Canada = Journal canadien de la medecine rurale : le journal officiel de la Societe de medecine rurale du Canada. 2005;10(3):163-8.

18. Melnick AL. Introduction to geographic information systems in public health: Jones & Bartlett Learning; 2002.

19. Nykiforuk CIJ, Flaman LM. Geographic Information Systems (GIS) for Health Promotion and Public Health: A Review. Health Promotion Practice. 2011;12(1):63-73.

20. Pundt H, Spangenberg T, Weinkauf R. WEB-BASED AND CONTEXT-SENSITIVE, MOBILE GEO-TOOLS TO SUPPORT SPATIAL DECISION MAKING IN HEALTH AND EMERGENCY MANAGEMENT. HEALTHINF 2010: PROCEEDINGS OF THE THIRD INTERNATIONAL CONFERENCE ON HEALTH INFORMATICS; 20102010. p. 469-+.

21. Rocha CM, Kruger E, McGuire S, Tennant M. The geographic distribution of patients seeking emergency dental care at the Royal Dental Hospital of Melbourne, Australia. COMMUNITY DENTAL HEALTH. 2013;30(3):149-54.

22. Schuler F, Ma M, Perkins J, inventorsSystem for determining priority discrepancies between audio data and records data, has electronic computing device for generating geographical map that includes priority discrepancies, and is configured for display on display device patent WO2022213023-A1

US2022318278-A1. 2022.

23. Shaw N, McGuire S. Understanding the use of geographical information systems (GISs) in health informatics research: a review. BMJ Health & Care Informatics. 2017;24(2).

24. Tanser FC, Le Sueur D. The application of geographical information systems to important public health problems in Africa. International journal of health geographics. 2002;1:1-9.

25. Tao Y, Wu P, inventorsEmergency evacuation method for evacuating people urgently to ensure safety of life and property of people, based on geographic information system (GIS) in event type such as natural disaster type, involves sending emergency evacuation information to target mobile device patent CN117082450-A.

26. Tomaszewski B, Judex M, Szarzynski J, Radestock C, Wirkus L. Geographic information systems for disaster response: A review. Journal of Homeland Security and Emergency Management. 2015;12(3):571-602.

27. Tsai M-K, Lee Y-C, Lu C-H, Chen M-H, Chou T-Y, Yau N-J. Integrating geographical information and augmented reality techniques for mobile escape guidelines on nuclear accident sites. JOURNAL OF ENVIRONMENTAL RADIOACTIVITY. 2012;109:36-44.

28. Tzavella K, Fekete A, Fiedrich F. Opportunities provided by geographic information systems and volunteered geographic information for a timely emergency response during flood events in Cologne, Germany. Natural Hazards. 2018;91:29-57.

29. Wang Z, Li H, Song J, Gong X, Chen N, Song C, et al., inventorsPublic health event emergency medical facility addressing method based on point of interest data, involves establishing a file geographic information database by using a file geographic information system software patent CN112232599-A.

30. Waring S, Zakos-Feliberti A, Wood R, Stone M, Padgett P, Arafat R. The utility of geographic information systems (GIS) in rapid epidemiological assessments following weather-related disasters: methodological issues based on the Tropical Storm Allison Experience. International journal of hygiene and environmental health. 2005;208(1-2):109-16.

31. Yu Z, Liu P, inventorsMethod for locating geographical position of graph convolution network model based on attention aggregate, involves inputting result obtained by training graph convolution network model to geographic position predictor of multilayer sensor patent CN116166865-A.

32. Zhen Z, Jing-min J, Liu F, editors. The application of geographic information system (GIS) in the field of public health. 2010 Second IITA International Conference on Geoscience and Remote Sensing; 2010: IEEE.

33. Zhen W, Li C, Chen R, Wei Z, Chen T, Yan J, inventorsMulti-scene city emergency sampling site addressing optimization method for city public health safety technical field, involves obtaining mobile phone signaling data of target city, interest point, and basic geographic information data patent CN115860213-A.
