# Supplementary material for: Knowledge attributes of public health management information systems used in health emergencies: a scoping review
Source: Front Public Health. 2025 Mar 20;12:1458867. doi: 10.3389/fpubh.2024.1458867 (PMC11969037; doi:10.3389/fpubh.2024.1458867)
Supplement: SUPPLEMENTARY DATA SHEET 4 — Supplementary Tables D1 to D13. [file Data_Sheet_4.zip › SupplementaryTables_D1_D13_SettingsPerHMIS/SupplementaryTable_D5_GPHIN.docx]

**Supplementary Table D5: Countries where GPHIN has been used.**

| **Author** | **Year of publication** | **Countries** | **Purpose** |
| --- | --- | --- | --- |
| Al-Imam et al (1) | 2017 | Middle East | Captagon: use and trade in the Middle East |
| Blench (2) | 2007 | na | Global public health intelligence network (GPHIN) |
| Carter et al (3) | 2020 | na | Redesigning GPHIN |
| Dion et al (4) | 2015 | China, West Africa, Jordan, H1N1 pandemic in Mexico | Bid data and GPHIN |
| Keller et al (5) | 2009 | Na | Unstructured Event-Based Reports for Disease Surveillance |
| Madoff & Li (6) | 2015 | Na | Web-Based Surveillance Systems for Human, Animal, & Plant dses |
| Mawudeku & Blench (7) | 2005 | na | Global public health intelligence network (GPHIN) |
| Mawudeku et al (8) | 2013 | na | The global public health intelligence network |
| Mykhalovskiy & Weir (9) | 2006 | na | GPHIN and early warning outbreak detection |
| Roberts & Elbe (10) | 2017 | na | Syndromic surveillance, algorithmic governmentality&health security |
| Wark (11) | 2021 | na | better global health security early-warning system post-COVID |
| Young et al (12) | 2015 | Global | monitoring the global spread of novel psychoactive substances |

**References**

1. Al-Imam A, Santacroce R, Roman-Urrestarazu A, Chilcott R, Bersani G, Martinotti G, et al. Captagon: use and trade in the Middle East. HUMAN PSYCHOPHARMACOLOGY-CLINICAL AND EXPERIMENTAL. 2017;32(3).

2. Blench M, editor Global public health intelligence network (GPHIN). Proceedings of Machine Translation Summit XI: Papers; 2007.

3. Carter D, Stojanovic M, Hachey P, Fournier K, Rodier S, Wang Y, et al. Global Public Health Surveillance Using Media Reports: Redesigning GPHIN. DIGITAL PERSONALIZED HEALTH AND MEDICINE; 20202020. p. 843-7.

4. Dion M, AbdelMalik P, Mawudeku A. Big Data and the Global Public Health Intelligence Network (GPHIN). Can Commun Dis Rep. 2015;41(9):209-14.

5. Keller M, Blench M, Tolentino H, Freifeld CC, Mandl KD, Mawudeku A, et al. Use of Unstructured Event-Based Reports for Global Infectious Disease Surveillance. EMERGING INFECTIOUS DISEASES. 2009;15(5):689-95.

6. Madoff LC, Li A. Web-Based Surveillance Systems for Human, Animal, and Plant Diseases. Microbiology spectrum. 2014;2(1):OH-0015-2012.

7. Mawudeku A, Blench M, editors. Global public health intelligence network (GPHIN). Proceedings of Machine Translation Summit X: Invited papers; 2005.

8. Mawudeku A, Blench M, Boily L, St. John R, Andraghetti R, Ruben M. The global public health intelligence network. Infectious disease surveillance. 2013:457-69.

9. Mykhalovskiy E, Weir L. The Global Public Health Intelligence Network and early warning outbreak detection: a Canadian contribution to global public health. Canadian journal of public health. 2006;97:42-4.

10. Roberts SL, Elbe S. Catching the flu: Syndromic surveillance, algorithmic governmentality and global health security. SECURITY DIALOGUE. 2017;48(1):46-62.

11. Wark W. Building a better global health security early-warning system post-COVID: The view from Canada. INTERNATIONAL JOURNAL. 2021;76(1):55-67.

12. Young MM, Dubeau C, Corazza O. Detecting a signal in the noise: monitoring the global spread of novel psychoactive substances using media and other open-source information. HUMAN PSYCHOPHARMACOLOGY-CLINICAL AND EXPERIMENTAL. 2015;30(4):319-26.
