# Supplementary material for: Knowledge attributes of public health management information systems used in health emergencies: a scoping review
Source: Front Public Health. 2025 Mar 20;12:1458867. doi: 10.3389/fpubh.2024.1458867 (PMC11969037; doi:10.3389/fpubh.2024.1458867)
Supplement: SUPPLEMENTARY DATA SHEET 4 — Supplementary Tables D1 to D13. [file Data_Sheet_4.zip › SupplementaryTables_D1_D13_SettingsPerHMIS/SupplementaryTable_D6_HealthMap.docx]

**Supplementary Table D6: Countries where HealthMap has been used.**

| **Author** | **Year of publication** | **Countries** |
| --- | --- | --- |
| Ahmed et al (1) | 2015 | USA |
| Bhatia et al (2) | 2021 | Guinea, Liberia, and Sierra Leone |
| Brownstein & Freifeld (3) | 2007 | na |
| Brownstein et al (4) | 2008 | Global |
| Carita (5) | 2014 | Na |
| Chen et al (6) | 2010 | na |
| Freifeld et al (7) | 2008 | Global (87 countries) |
| Ganser (8) | 2020 | Twenty-four countries from 15 influenza transmission zones were chosen to evaluate the performance of event based surveillance (EBS) on a global scale: Argentina, Australia, Brazil, Bulgaria, China, Costa Rica, Ecuador, Egypt, France, Germany, Greece, India, Iran, Mexico, Nigeria, Russia, Saudi Arabia, South Africa, Sweden, Thailand, United Kingdom, United States, Uruguay, and Vietnam. |
| Hossain & Househ(9) | 2016 | Arab world and North Africa |
| Lyon et al (10) | 2012 | Global |
| Majumder, et al (11) | 2016 | Colombia |
| Nelson (12) | 2008 | na |
| Schwind et al (13) | 2014 | Uganda, Tanzania, Cameroon, Bolivia, Bangladesh |
| Sonricker et al (14) | 2010 | na |
| Valentin et al (15) | 2023 | Not specified |

**References**

1. Ahmed SS, Oviedo-Orta E, Mekaru SR, Freifeld CC, Tougas G, Brownstein JS. Surveillance for <i>Neisseria meningitidis</i> Disease Activity and Transmission Using Information Technology. PLOS ONE. 2015;10(5).

2. Bhatia S, Lassmann B, Cohn E, Desai AN, Carrion M, Kraemer MUG, et al. Using digital surveillance tools for near real-time mapping of the risk of infectious disease spread. NPJ DIGITAL MEDICINE. 2021;4(1).

3. Brownstein JS, Freifeld C. HealthMap: the development of automated real-time internet surveillance for epidemic intelligence. Weekly releases (1997–2007). 2007;12(48):3322.

4. Brownstein JS, Freifeld CC, Reis BY, Mandl KD. Surveillance Sans Frontières: Internet-Based Emerging Infectious Disease Intelligence and the HealthMap Project. PLOS Medicine. 2008;5(7):e151.

5. Carita A. Healthmap. Reference Reviews. 28: Emerald Group Publishing Limited; 2014. p. 30-1.

6. Chen H, Zeng D, Yan P, Chen H, Zeng D, Yan P. HealthMap. Infectious Disease Informatics: Syndromic Surveillance for Public Health and BioDefense. 2010:183-6.

7. Freifeld CC, Mandl KD, Reis BY, Brownstein JS. HealthMap: global infectious disease monitoring through automated classification and visualization of Internet media reports. Journal of the American Medical Informatics Association. 2008;15(2):150-7.

8. Ganser IZ. Evaluation of Event-Based Internet Biosurveillance for Multi-Regional Detection of Seasonal Influenza Onset2020 2020.

9. Hossain N, Househ MS, editors. Using HealthMap to Analyse Middle East Respiratory Syndrome (MERS) Data. ICIMTH; 2016.

10. Lyon A, Nunn M, Grossel G, Burgman M. Comparison of Web-Based Biosecurity Intelligence Systems: BioCaster, EpiSPIDER and HealthMap. Transboundary and Emerging Diseases. 2012;59(3):223-32.

11. Majumder MS, Santillana M, Mekaru SR, McGinnis DP, Khan K, Brownstein JS. Utilizing Nontraditional Data Sources for Near Real-Time Estimation of Transmission Dynamics During the 2015-2016 Colombian Zika Virus Disease Outbreak. JMIR public health and surveillance. 2016;2(1):e30-e.

12. Nelson R. HealthMap: the future of infectious diseases surveillance? The Lancet Infectious Diseases. 2008;8(10):596.

13. Schwind JS, Wolking DJ, Brownstein JS, Mazet JAK, Smith WA, Consortium P. Evaluation of Local Media Surveillance for Improved Disease Recognition and Monitoring in Global Hotspot Regions. PLOS ONE. 2014;9(10).

14. Sonricker AL, Freifeld CC, Keller M, Brownstein JS. HealthMap. Biosurveillance: Chapman and Hall/CRC; 2010. p. 133-46.

15. Valentin S, Boudoua B, Sewalk K, Arınık N, Roche M, Lancelot R, et al. Dissemination of information in event-based surveillance, a case study of Avian Influenza. PLoS One. 2023;18(9):e0285341.
