# Supplementary material for: Knowledge attributes of public health management information systems used in health emergencies: a scoping review
Source: Front Public Health. 2025 Mar 20;12:1458867. doi: 10.3389/fpubh.2024.1458867 (PMC11969037; doi:10.3389/fpubh.2024.1458867)
Supplement: SUPPLEMENTARY DATA SHEET 4 — Supplementary Tables D1 to D13. [file Data_Sheet_4.zip › SupplementaryTables_D1_D13_SettingsPerHMIS/SupplementaryTable_D7_HDX.docx]

**Supplementary Table D7: Countries where HDX has been used.**

| **Author** | **Year of publication** | **Countries** |
| --- | --- | --- |
| Abuoda et al (1) | 2021 | na |
| Balsari et al (2) | 2022 | USA |
| Berens et al (3) | 2016 | na |
| Charniga et al (4) | 2021 | Columbia |
| Charniga et al (5) | 2021 | Columbia |
| Gao et al (6) | 2022 | Varied regions |
| Gibbs et tal (7) | 2022 | Ghana |
| Hierink et al (8) | 2022 | Philippines |
| Kelly(9) | 2020 | UK |
| MacPherson et al (10) | 2023 | Malawi |
| Metwally et al (11) | 2023 | Egypt |
| Moulds et al (12) | 2022a | Ghana |
| Moulds et al (12) | 2022b | Ghana |
| Moulds et al (13) | 2022c | Ghana |
| Nyabinwa et al (14) | 2020 | Rwanda |
| Nyakarahuka et al (15) | 2023 | Uganda |
| Paulus et al (16) | 2016 | Nepal |
| Ross(17) | 2018 | USA |
| Ross et al (18) | 2022 | Bangladesh |
| Telford (19) | 2020 | na |
| Weaver et al (20) | 2019 | Global |
| Yan et al (21) | 2022 | Haiti |

**References**

1. Abuoda G, Hendrix C, Campo S, editors. Automatic Tag Recommendation for the UN Humanitarian Data Exchange. BIRDS+ WEPIR@ CHIIR; 2021.

2. Balsari S, Buckee C, Chan J, Schroeder A. The Use of Human Mobility Data in Public Health Emergencies. 2022.

3. Berens J, Raymond N, Shimshon G, Verhulst S, Bernholz L. The humanitarian data ecosystem: The case for collective responsibility. Stanford Center on Philanthropy and Civil Society. <https://pacscenter> …; 2016.

4. Charniga K, Cucunuba ZM, Walteros DM, Mercado M, Prieto F, Ospina M, et al. Maps of ZVD incidence in Colombia. Figshare2021.

5. Charniga K, Cucunuba ZM, Walteros DM, Mercado M, Prieto F, Ospina M, et al. Maps of ZIKV-associated neurological complications by department in Colombia. Figshare2021.

6. Gao W, Sanna M, Tsai MK, Wen CP. Percentage of COVID-19 infected healthcare workers in severe conditions in different geographic locations and over three ten-day periods, based on symptom onset dates. Figshare2020.

7. Gibbs H, Liu Y, Abbott S, Baffoe-Nyarko I, Laryea DO, Akyereko E, et al. Estimates of Rt in individual districts. Figshare2022.

8. Hierink F, Margutti J, Van Den Homberg M, Ray N. Overview of all results. Figshare2022.

9. Kelly L. Humanitarian evidence summary No. 7. 2020.

10. MacPherson EE, Mankhomwa J, Dixon J, Pongolani R, Phiri M, Feasey N, et al. Location of households sampled for interview. Figshare2023.

11. Metwally AM, Nassar MS, El-Din EM, Abdallah AM, Khadr Z, Abouelnaga MW, et al. Map of the 27 Egyptians governorates distributed within the four geographic regions (adapted using data from the Humanitarian Data Exchange under the CC BY-IGO license [18]. Figshare2023.

12. Moulds S, Chan ACH, Tetteh JD, Bixby H, Owusu G, Agyei-Mensah S, et al. Percentage of households drinking sachet water in Ghanas 170 districts. Figshare2022.

13. Moulds S, Chan ACH, Tetteh JD, Bixby H, Owusu G, Agyei-Mensah S, et al. Most common source of drinking water in Ghanas 170 districts. Figshare2022.

14. Nyabinwa P, Kashongwe OB, Hirwa CD, Bebe BO. Additional file 1 of Perception of farmers about endometritis prevention and control measures for zero-grazed dairy cows on smallholder farms in Rwanda. Figshare2020.

15. Nyakarahuka L, Kyondo J, Telford C, Whitesell A, Tumusiime A, Mulei S, et al. Sampled districts and their corresponding seroprevalence of Crimean-Congo hemorrhagic fever virus IgG antibodies in cattle, sheep and goats (Open-source shapefiles for Uganda district boundaries were downloaded from the Humanitarian Data Exchange (Humanitarian Data Exchange, 2020) and water bodies files from the World Bank website (The World Bank, 2022)). Figshare2023.

16. Paulus D, Meesters K, Van de Walle BA, editors. Turning data into action: supporting humanitarian field workers with open data. Iscram; 2018.

17. Ross DW. Research Tools In The Phase Zero Digital Toolbox2018. Available from: <https://www.jstor.org/stable/pdf/resrep24319.4.pdf>.

18. Ross YB, Hoque M, Blanton JD, Kennedy ED, Rana MS, Tahmina S, et al. Map of Bangladesh survey sites and the distribution of household surveys by survey site. Figshare2022.

19. Telford S. Case Study-The Humanitarian Data Exchange: Critical Decisions, Key Results and The Road Ahead2020. Available from: <https://centre.humdata.org/wp-content/uploads/2020/09/hdxcasestudy.pdf>.

20. Weaver C, Powell J, Leson H. Development assistance and humanitarian action. 2019. In: The State of Open Data [Internet]. [77].

21. Yan LD, McNairy ML, Devieux JG, Pierre JL, Dade E, Sufra R, et al. Map of census blocks sampled in Haiti CVD cohort. Figshare2022.
