# Supplementary material for: Knowledge attributes of public health management information systems used in health emergencies: a scoping review
Source: Front Public Health. 2025 Mar 20;12:1458867. doi: 10.3389/fpubh.2024.1458867 (PMC11969037; doi:10.3389/fpubh.2024.1458867)
Supplement: SUPPLEMENTARY DATA SHEET 4 — Supplementary Tables D1 to D13. [file Data_Sheet_4.zip › SupplementaryTables_D1_D13_SettingsPerHMIS/SupplementaryTable_D8_mHealth.docx]

**Supplementary Table D8: Countries where mHealth has been used.**

| **Author** | **Year of publication** | **Countries** |
| --- | --- | --- |
| Abaza & Marshcholleck (1) | 2017 | North America had the highest number of applications (n=74) followed by Europe (n=50), Asia (n=44), Africa (n=25), and Australia (n=9) |
| Adeniyi et al (2) | 2020 | Not stated |
| Albabtain et al (3) | 2014 | Developing world |
| Bhattacharya et al (4) | 2018 | India |
| Brinkel et al (5) | 2014 | Sub Saharan Africa |
| Burmer et al (6) | 2019 | USA |
| Dehling et al (7) | 2015 | na |
| Erbele et al (8) | 2021 | na |
| Esiyok et al (9) | 2023 | Turkey |
| Grijalva-Eternod et al (10) | 2023 | Somalia |
| Jabeen et al (11) | 2023 | Pakistan |
| Kahn et al (12) | 2010 | Low - & middle-income countries |
| Kallander et al (13) | 2013 | Developing countries |
| Littman-Quinn et al (14) | 2011 | Botswana |
| Mohanty et al (15) | 2019 | Global |
| Pande et al (16) | 2012 | India |
| Roncero et al (17) | 2020 | Not stated |
| Rumsfeld et al (18) | 2016 | USA |
| Sanjeev & Anuradha (19) | 2014 | Developing countries |
| Shahriar & Pradeep(20) | 2010 | Kenya, Uganda and Zambia (+ 20 countries of sub-Saharan Africa) Mexico, Vietnam, South Africa, Thailand, Philippines Uganda, Caribbean, & Guatemala |
| Singh et al (21) | 2020 | China, France, Germany, Iran, Italy, Russia, Spain, Turkey, the United Kingdom, and the United States , Hong Kong, Iceland, India, Indonesia, Malaysia, Poland, Singapore, South Korea, and Taiwan. |
| Steinbul et al (22) | 2013 | Not stated |
| Speciale & Freytsis (23) | 2013 | na |
| Steinhubl et al (24) | 2019 | USA |
| Tamrat & Kachnowski (25) | 2012 | Rural Bangladesh, Ghana, The Gambia, Uganda, India, Pakistan, Indonesia, remote locations in Pakistan, Serbia |
| Vahidi et al (26) | 2021 | Iran |
| Varshney (27) | 2014 | Not stated |
| Williams et al (28) | 2020 | Not stated |
| Zhang et al (29) | 2020 | China |

**References**

1. Abaza H, Marschollek M. mHealth application areas and technology combinations. Methods of information in medicine. 2017;56(S 01):e105-e22.

2. Adeniyi EA, Awotunde JB, Ogundokun RO, Kolawole PO, Abiodun MK, Adeniyi AA. Mobile health application and COVID-19: Opportunities and challenges. Journal of Critical Reviews. 2020;7(15):3481-8.

3. Albabtain AF, AlMulhim DA, Yunus F, Househ MS. The role of mobile health in the developing world: a review of current knowledge and future trends. Journal of Selected Areas in Health Informatics. 2014;4(2):10-5.

4. Bhattacharya S, Kumar A, Kaushal V, Singh A. Applications of m-Health and e-Health in Public Health Sector: the challenges and opportunities. International Journal of Medicine and Public Health. 2018;8(2).

5. Brinkel J, Krämer A, Krumkamp R, May J, Fobil J. Mobile phone-based mHealth approaches for public health surveillance in sub-Saharan Africa: a systematic review. International journal of environmental research and public health. 2014;11(11):11559-82.

6. Burner E, Mercado J, Hernandez-Saenz A, Peters A, Baezconde-Garbanati L, Arora S, et al. Design and patient characteristics of the randomized controlled trial TExT-MED plus FANS A test of mHealth augmented social support added to a patient-focused text-messaging intervention for emergency department patients with poorly controlled diabetes. CONTEMPORARY CLINICAL TRIALS. 2019;80:1-8.

7. Dehling T, Gao F, Schneider S, Sunyaev A. Exploring the far side of mobile health: information security and privacy of mobile health apps on iOS and Android. JMIR mHealth and uHealth. 2015;3(1):e3672.

8. Eberle C, Loehnert M, Stichling S. Effectivness of specific mobile health applications (mHealth-apps) in gestational diabtetes mellitus: a systematic review. BMC PREGNANCY AND CHILDBIRTH. 2021;21(1).

9. EŞİYOK A, DİVANOĞLU SU, ÇELİK R. Digitalization in Healthcare-Mobile Health (M-Health) Applications. Aksaray Üniversitesi İktisadi ve İdari Bilimler Fakültesi Dergisi. 2023;15(2):165-74.

10. Grijalva-Eternod CS, Jelle M, Mohamed H, Waller K, Osman Hussein B, Barasa E, et al. Evaluation of conditional cash transfers and mHealth audio messaging in reduction of risk factors for childhood malnutrition in internally displaced persons camps in Somalia: A 2 × 2 factorial cluster-randomised controlled trial. PLOS Medicine. 2023;20(2):e1004180.

11. Jabeen R, Mohammad A. Leverage of mHealth integration in Maternal and child health services and COVID-19 Pandemic. JOURNAL OF THE PAKISTAN MEDICAL ASSOCIATION. 2023;73(2):370-3.

12. Kahn JG, Yang JS, Kahn JS. ‘Mobile’health needs and opportunities in developing countries. Health affairs. 2010;29(2):252-8.

13. Källander K, Tibenderana JK, Akpogheneta OJ, Strachan DL, Hill Z, ten Asbroek AH, et al. Mobile health (mHealth) approaches and lessons for increased performance and retention of community health workers in low-and middle-income countries: a review. Journal of medical Internet research. 2013;15(1):e17.

14. Littman-Quinn R, Chandra A, Schwartz A, Fadlelmola FM, Ghose S, Luberti AA, et al., editors. mHealth applications for telemedicine and public health intervention in Botswana. 2011 IST-Africa Conference Proceedings; 2011: IEEE.

15. Mohanty B, Chughtai A, Rabhi F. Use of Mobile Apps for epidemic surveillance and response–availability and gaps. Global Biosecurity. 2019;1(1).

16. Pande A, Kimbahune S, Bondale N, Shinde R, Shanbhag S, Ramaswamy S. Distributed Processing and Internet Technology to Solve Challenges of Primary Healthcare in India. DISTRIBUTED COMPUTING AND INTERNET TECHNOLOGY; 20122012. p. 188-+.

17. Roncero AP, Marques G, Sainz-De-Abajo B, Martín-Rodríguez F, del Pozo Vegas C, Garcia-Zapirain B, et al. Mobile health apps for medical emergencies: systematic review. JMIR mHealth and uHealth. 2020;8(12):e18513.

18. Rumsfeld JS, Brooks SC, Aufderheide TP, Leary M, Bradley SM, Nkonde-Price C, et al. Use of Mobile Devices, Social Media, and Crowdsourcing as Digital Strategies to Improve Emergency Cardiovascular Care A Scientific Statement From the American Heart Association. CIRCULATION. 2016;134(8):E87-E108.

19. Sanjeev D, Anuradha D. Mobile-health technology: Can it Strengthen and improve public health systems of other developing countries as per Indian strategies? A systematic review of the literature. International Journal of medicine and public Health. 2014;4(1).

20. Shahriar A, Pradeep R. mHealth-an ultimate platform to serve the unserved. Yearbook of medical informatics. 2010;19(01):94-100.

21. Singh HJL, Couch D, Yap K. Mobile health apps that help with COVID-19 management: scoping review. JMIR nursing. 2020;3(1):e20596.

22. Steinhubl SR, Muse ED, Topol EJ. Can mobile health technologies transform health care? Jama. 2013;310(22):2395-6.

23. Speciale AM, Freytsis M. mHealth for Midwives: A Call to Action. JOURNAL OF MIDWIFERY & WOMENS HEALTH. 2013;58(1):76-82.

24. Steinhubl SR, Edwards AM, Waalen J, Zambon R, Mehta R, Ariniello L, et al. HEALTHCARE RESOURCE UTILIZATION ASSOCIATED WITH ELECTROCARDIOGRAPH (ECG) SENSOR PATCH SCREENING FOR ATRIAL FIBRILLATION (AF): RESULTS FROM THE MHEALTH SCREENING TO PREVENT STROKES (MSTOPS) TRIAL. JOURNAL OF THE AMERICAN COLLEGE OF CARDIOLOGY. 2019;73(9):296-.

25. Tamrat T, Kachnowski S. Special delivery: an analysis of mHealth in maternal and newborn health programs and their outcomes around the world. Maternal and child health journal. 2012;16(5):1092-101.

26. Vahidi H, Taleai M, Yan W, Shaw R. Digital Citizen Science for Responding to COVID-19 Crisis: Experiences from Iran. INTERNATIONAL JOURNAL OF ENVIRONMENTAL RESEARCH AND PUBLIC HEALTH. 2021;18(18).

27. Varshney U. Mobile health: Four emerging themes of research. Decision Support Systems. 2014;66:20-35.

28. Williams SY, Adeyemi SO, Eyitayo JO, Odeyemi OE, Dada OE, Adesina MA, et al. Mobile health technology (Mhealth) in combating COVID-19 pandemic: Use, challenges and recommendations. Electronic Journal of Medical and Educational Technologies. 2020;13(4):em2018.

29. Zhang Z, Zhang L, Zheng J, Xiao H, Li Z. COVID-19-Related Disruptions and Increased mHealth Emergency Use Intention: Experience Sampling Method Study. JMIR MHEALTH AND UHEALTH. 2020;8(12).
