# Supplementary material for: Knowledge attributes of public health management information systems used in health emergencies: a scoping review
Source: Front Public Health. 2025 Mar 20;12:1458867. doi: 10.3389/fpubh.2024.1458867 (PMC11969037; doi:10.3389/fpubh.2024.1458867)
Supplement: SUPPLEMENTARY DATA SHEET 5 — Supplementary Table E. [file Data_Sheet_5.docx]

**Supplementary table E: Other IMS used in public health emergencies.**

| **Name of IMs** | **Description & Website (s)** |
| --- | --- |
| Strategic Partnership for Health Security and Emergency Preparedness (SPH) Portal | An interactive digital platform that facilitates sharing and exchanging the latest information on multisectoral health security investments, activities, and capacities on a national, regional, and global scale  <https://extranet.who.int/sph/> |
| EPICENTRE | An initiative of Médecins Sans Frontières (Doctors Without Borders), this system conducts field epidemiology activities, research, training, and provides technical support in response to outbreaks.  <https://epicentre.msf.org/> |
| Health Emergencies and Disaster Risk Management (Health EDRM) Research and Knowledge Hub | WHO Centre for Health Development (WHO Kobe Centre, WKC) is a global research centre and has conducted research projects on Health EDRM  <https://extranet.who.int/kobe_centre/en/what_we_do/health-emergencies> |
| Epi Info™ | A public domain software package developed by the Centers for Disease Control and Prevention (CDC) for epidemiologists to manage data and visualize and analyze outbreaks.  <https://www.cdc.gov/epiinfo/index.html> |
| Event Management System for International Health Regulations (IHR) | A web-based platform used to share and manage public health events as per the International Health Regulations.  <https://www.who.int/news-room/events> |
| Electronic Health Records (EHRs) | These can be adapted for emergency scenarios to quickly retrieve patient medical histories, allergies, and other crucial information that could affect treatment decisions during emergencies.  <https://www.healthit.gov/faq/what-electronic-health-record-ehr>  <https://www.cms.gov/priorities/key-initiatives/e-health/records> |
| Electronic Health Records (EHR) with Epidemic Modules | Many modern EHR systems now come with modules specifically designed to monitor, track, and report epidemic or pandemic cases.  <https://www.ucl.ac.uk/health-informatics/study/health-data-science/modules-health-data-science/extra-module/principles-epidemiology-applied> |
| GOARN (Global Outbreak Alert and Response Network) | An early warning tool that uses machine learning to scan internet media to detect and report potential disease outbreaks.  <https://goarn.who.int/> |
| World Health Organization (WHO) Global Health Emergency Dashboard | This is an online platform that provides up-to-date information about ongoing global health emergencies, such as epidemics and natural disasters.  <https://extranet.who.int/publicemergency> |
| Centers for Disease Control and Prevention (CDC) Emergency Management Tools | The CDC provides various tools and training materials for emergency preparedness and response, including the Emergency Management Program, which supports the management of emergency response operations.  [https://www.cdc.gov/reproductivehealth]/emergency/tools.html](https://www.cdc.gov/reproductivehealth%5d/emergency/tools.html)  <https://www.cdc.gov/infectioncontrol/tools/> |
| Online Training Platforms | Websites and platforms that provide online courses, webinars, and resources related to emergency preparedness, outbreak response, and more. Examples include Coursera's courses on global health crises and disease outbreaks.  <https://www.coursera.org/>  <https://agora.unicef.org/>  <https://kayaconnect.org/>  <https://www.disasterready.org/> |
| Knowledge Repositories | Databases or digital libraries containing studies, guidelines, best practices, and other resources specifically related to health emergencies.  <https://www.who.int/europe/emergencies/resources/for-health-professionals> |
| Communication Platforms | Systems like WhatsApp, Telegram, or custom-built platforms for health workers to rapidly share updates, ask questions, and collaborate on problem-solving during emergencies.  <https://doi.org/10.1016/j.ijinfomgt.2015.07.001>  <https://www.ncbi.nlm.nih.gov/pmc/articles/PMC4029126/> |
| Crisis Resource Management Systems | These platforms manage resources such as beds, ventilators, and other critical supplies, ensuring they are optimally allocated during a health crisis.  <https://go-arc.com/solutions/critical-event-management/> |
